# Investigating the diastereoselective synthesis of a macrocycle under Curtin–Hammett control†

**DOI:** 10.1039/d3sc05715a

**Published:** 2024-03-05

**Authors:** Angus Yeung, Martijn A. Zwijnenburg, Georgia R. F. Orton, Jennifer H. Robertson, Timothy A. Barendt

**Affiliations:** a School of Chemistry, University of Birmingham Edgbaston Birmingham B15 2TT UK t.a.barendt@bham.ac.uk; b Department of Chemistry, University College London 20 Gordon Street London WC1H 0AJ UK

## Abstract

This work sheds new light on the stereoselective synthesis of chiral macrocycles containing twisted aromatic units, valuable π-conjugated materials for recognition, sensing, and optoelectronics. For the first time, we use the Curtin–Hammett principle to investigate a chiral macrocyclisation reaction, revealing the potential for supramolecular π–π interactions to direct the outcome of a dynamic kinetic resolution, favouring the opposite macrocyclic product to that expected under reversible, thermodynamically controlled conditions. Specifically, a dynamic, racemic perylene diimide dye (1 : 1 *P* : *M*) is strapped with an enantiopure (*S*)-1,1′-bi-2-naphthol group (*P*-BINOL) to form two diastereomeric macrocyclic products, the homochiral macrocycle (*PP*) and the heterochiral species (*PM*). We find there is notable selectivity for the *PM* macrocycle (dr = 4 : 1), which is rationalised by kinetic templation from intramolecular aromatic non-covalent interactions between the *P*-BINOL π-donor and the *M*-PDI π-acceptor during the macrocyclisation reaction.

## Abstract 2

The Curtin–Hammett principle is used to understand the diastereoselective synthesis of a heterochiral over a homochiral macrocycle, both of which are configurationally stable chiral macrocycles.

## Graphical abstract

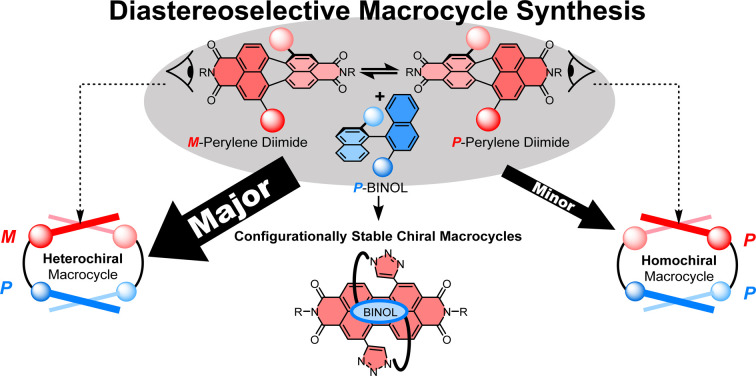

## Introduction

The balance between thermodynamics and kinetics determines the distribution of products in a chemical reaction. An understanding of these fundamental principles in the context of chiral molecules is essential for designing new and efficient stereoselective syntheses, including for pharmaceuticals,^1^ chemical sensing,^2^ and chiroptical materials,^3^ as well as unravelling the origins of single handedness in nature.^4^ Here, supramolecular chemistry plays a privileged role because the tuning of non-covalent interactions between molecules can be used to direct the stereochemical outcome of a chemical reaction^5^ or the self-assembly of a chiral material.^6^ In the latter case, chiral self-assembly mainly operates under reversible conditions (*i.e.*, thermodynamic control),^7^ which means the selection for homochiral or heterochiral products is dictated by their relative energies.^8^ However, a deeper understanding of the role of supramolecular chemistry in a dynamic kinetic resolution is essential for directing the stereochemical outcome of a reaction between interacting chiral molecules under irreversible conditions.

**Fig. 1:**
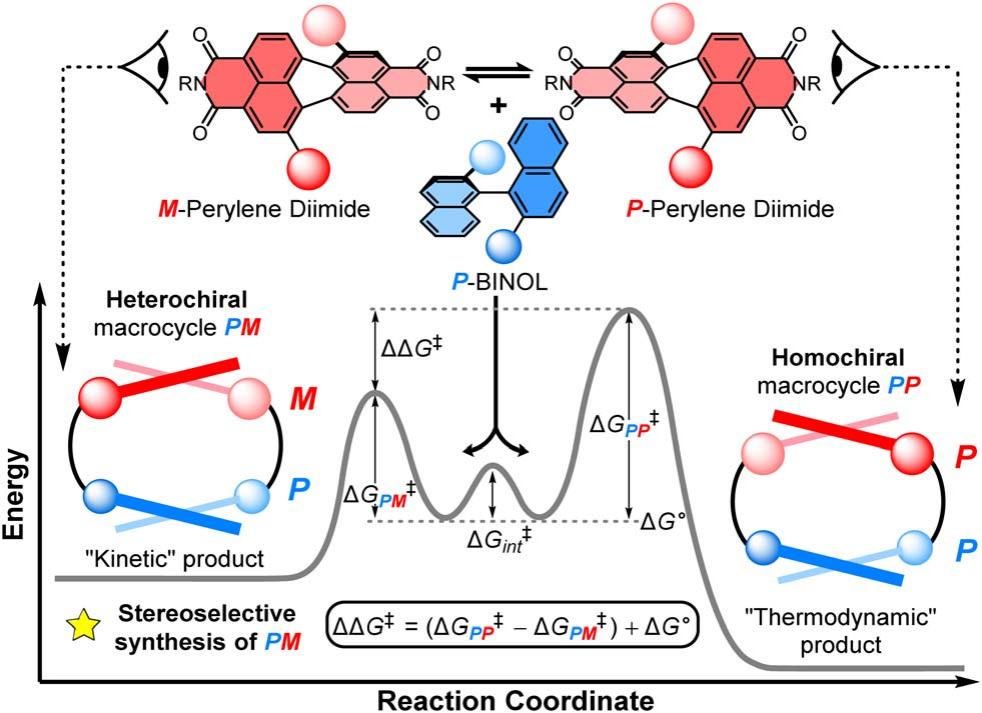
The diastereoselective synthesis of a heterochiral (*PM*) over a homochiral (*PP*) macrocycle under Curtin–Hammett control. Here, ΔΔ*G*^‡^ = (Δ*G*^‡^_*PP*_ − Δ*G*^‡^_*PM*_) + Δ*G*°; Δ*G*^‡^_*PP*/*PM*_ = the activation energy of *PP*/*MM* macrocycles; Δ*G*° = the relative free energy of the interconverting precursors; and Δ*G*^‡^_int_ = the activation energy of this interconversion.

Non-covalent kinetic templates can favour the formation of a particular product under irreversible reaction conditions,^9^ including the synthesis of macrocycles which have huge value across supramolecular chemistry.^10^ While often being challenging to design, the impact of templating interactions in reactions under kinetic control can be interpreted through the Curtin–Hammett principle, which states that the outcome of a dynamic kinetic resolution is dependent on the relative free energy of the interconverting precursors (*i.e.*, Δ*G*° in Fig. 1) and the relative activation energies (*i.e.*, Δ*G*^‡^_*PP*_ − Δ*G*^‡^_*PM*_).^11^ Indeed, the Curtin–Hammett principle explains the distribution of products in irreversible chemical transformations, including stereoselective reactions on dynamic heterocycles,^12^ as well as understanding the interactions between conformationally flexible macrocycles and proteins^13^ and, more recently, the operation of Brownian ratches in molecular machines.^14^ However, to the best of our knowledge, an investigation of Curtin–Hammett control in the context of a chiral macrocyclisation reaction (*i.e.*, the ring-closing step itself) is unprecedented, with such a development having important consequences for the templated stereoselective synthesis of chiral materials under kinetic control.

Perylene diimides (PDIs) are a class of functional organic dyes and outstanding building blocks for studying chiral molecular systems.^15^ This is because PDIs may be twisted through substitution at their bay positions (1, 6, 7, 12) to generate atropisomers (*P* or *M*)‡‡Throughout, we use the chiral nomenclature of *P*/*M* for PDIs, which is based on the Cahn–Ingold–Prelog priority rules for molecules with axial chirality.^23,46^ This ensures clear comparisons against the axial chirality of BINOL (*P*/*M*). with distinct chiroptical spectra,^16^ while their potential for π–π aromatic stacking interactions,^17^ including with complementary π-donors,^18^ gives rise to an extensive supramolecular chemistry.^15^ Alongside functional complexes,^19^ cages,^20^ and polymers,^21^ macrocycles containing twisted PDIs have been developed to explore homo- and heterochirality.^22^ We have reported a bis-PDI macrocycle that is predominantly homochiral (diastereomeric ratio [dr] > 88 : 12 *MM*/*PP* : *PM*) due to complementary π–π stacking.^23^ However, in this system, as for most bis-PDI macrocycles,^22*a*–*c*^ the PDI units are not configurationally stable (*i.e.*, their atropisomers can freely interconvert), hence the macrocycle's chiral conformation is under thermodynamic control, which makes them unsuitable for investigating kinetic templation under Curtin–Hammett control.

Herein, we report the first stereoselective synthesis of a configurationally stable PDI macrocycle (Fig. 1). We impose configurational stability on a racemic 1,7-disubstituted PDI dye (1 : 1 *P* : *M*) by strapping it with a (*S*)-1,1′-bi-2-naphthol group (*P*-BINOL), enabling us to isolate concurrent heterochiral (*PM*) and homochiral (*PP*) macrocycles. The contrast between BINOL, which is always chirally locked, and PDI, whose atropisomers rapidly interconvert prior to, but not after, macrocylisation, is critical to a diastereoselective macrocycle synthesis under Curtin–Hammett control (Fig. 1). We discover that the use of *P*-BINOL impacts the chirality of the PDI during the ring-closing reaction, affording diastereoselectivity for the heterochiral macrocycle (dr = 4 : 1 *PM* : *PP*). This is the opposite outcome to that expected from a reversible, thermodynamically controlled synthesis, since the homochiral macrocycle is predicted, by calculations, to be significantly lower in energy. While BINOL has been integrated into macrocycles,^24^ including for the dynamic kinetic resolution of 2,2′-bipyridine *N*,*N*′-dioxide atropisomers,^25^ it has never been combined with a twisted PDI dye, which here enables us to explore the possibility of supramolecular templation as an origin of stereoselectivity. These investigations point towards the importance of aromatic non-covalent interactions between the *P*-BINOL π-donor and *M*-PDI π-acceptor, demonstrating the potential for π–π templation to be exploited in the stereoselective synthesis of π-conjugated atropisomeric materials under irreversible conditions.^26^

## Results and discussion

### Synthesis and characterisation

**Fig. 2:**
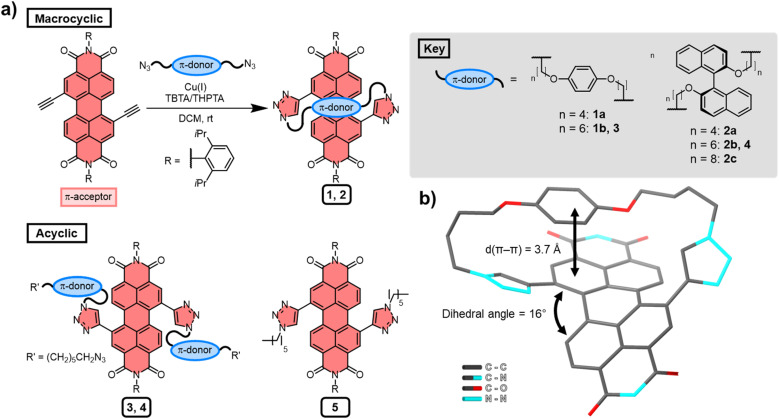
(a) The synthesis of macrocyclic and acyclic compounds 1–5 and (b) X-ray crystal structure of macrocycle 1a. For clarity, hydrogen atoms and the 2,6-diisopropylphenyl imide substituents have been removed from the structure. The Cu(i) coordinating ligands are tris((1-benzyl-4-triazolyl)methyl)amine (TBTA) or tris(3-hydroxypropyltriazolylmethyl)amine (THPTA).

Several macrocyclic (1, 2) and acyclic (3–5) compounds were prepared using multi step syntheses, the final step of which uses copper(i)-catalysed azide–alkyne cycloaddition (CuAAC) “click” chemistry (Fig. 2a) to connect the PDI π-acceptor to a planar (hydroquinone) or twisted (*P*-BINOL) π-donor. The macrocycles were synthesised under high dilution conditions to favour macrocyclisation, using stoichiometric amounts of a PDI bis-alkyne and bis-azide *n*-alkyl linker (Fig. 2a). The desired [1 + 1] macrocycles were formed in good yields (up to 53%), with major side products being larger [2 + 2] and [3 + 3] macrocyclic analogues. All the synthetic procedures and complete characterisation data are provided in the ESI (Section 2†).

All the derivatives of 1 and 2 were characterised by ^1^H and ^13^C NMR spectroscopy, which, alongside high-resolution mass spectrometry, confirmed they are [1 + 1] macrocycles. From this compound library, PDI-hydroquinone macrocycle 1a yielded single crystals that were suitable for X-ray diffraction (ESI, Section 7†). Importantly, the crystal structure of 1a shows the PDI is twisted (16°), with one half of the π-electron deficient unit forming a π–π interaction (*d*(π–π) = 3.7 Å)^27^ with the π-electron rich hydroquinone group (Fig. 2b). We note that, in contrast to our previous bis-triazole PDI-based macrocycle,^23^ the crystal structure of 1a shows that the triazole heterocycles are directed towards the macrocyclic cavity, most likely because this reduces ring strain in the shorter cyclic framework.

**Fig. 3:**
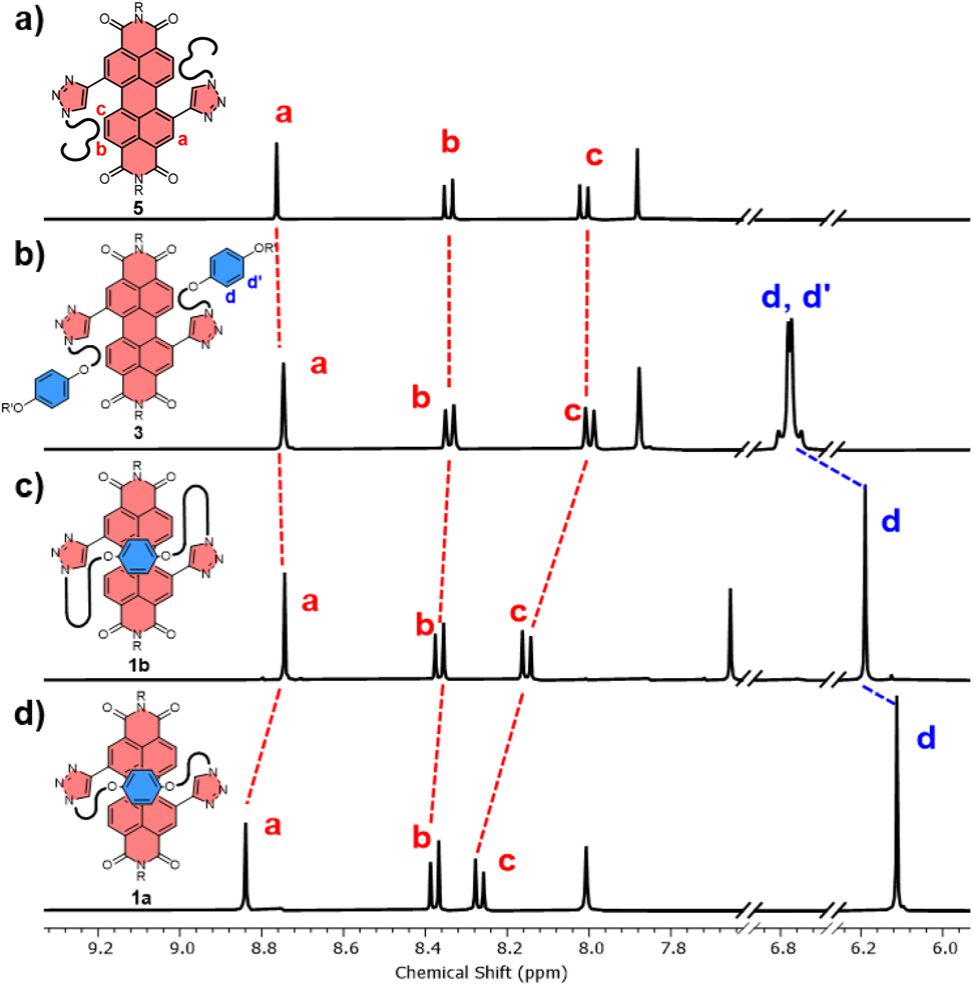
Stacked ^1^H NMR spectra of (a) acyclic bis-triazole PDI 5, (b) acyclic bis-hydroquinone PDI 3, (c) hydroquinone-PDI macrocycles 1b and, (d) 1a (CDCl_3_, 298 K, 400 MHz).

**Fig. 4:**
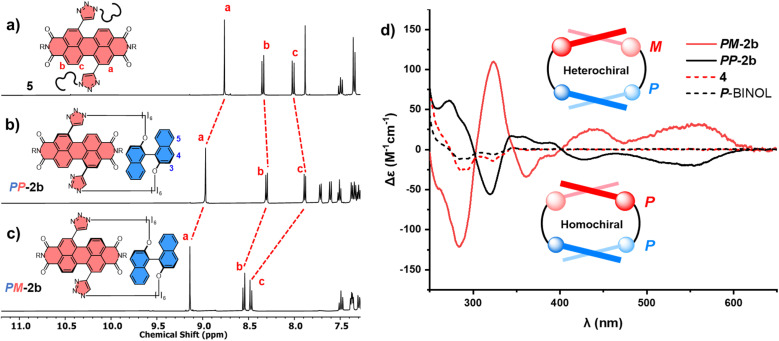
Stacked ^1^H NMR spectra of (a) acyclic bis-triazole PDI 5, (b) homochiral PDI–*P*-BINOL macrocycle *PP*-2b, and (c) heterochiral PDI–*P*-BINOL macrocycle *PM*-2b (CDCl_3_, 298 K, 400 MHz); and (d) the circular dichroism (CD) spectra of *PP*-2b and *PM*-2b which are opposite in the PDI-only region (*λ* = 350–600 nm), where neither *P*-BINOL nor acyclic control 4 exhibits a CD spectrum in this region (CHCl_3_, 298 K).

The significant (up) downfield shifts of protons (H_d,d′_) H_a–c_ in the ^1^H NMR spectrum of macrocycles 1a (Fig. 3d) and 1b (Fig. 3c), relative to acyclic analogues 3 and 5 (Fig. 3a and b), indicates the macrocyclic framework preorganises the π-conjugated units in solution, providing the potential for intramolecular aromatic stacking interactions (*vide infra*). This is also true for macrocycles containing the twisted *P*-BINOL unit since 2a–c exhibit the same pattern of proton shifts (Fig. 4 and S11†). Moreover, NOESY NMR spectroscopy shows cross-peaks between PDI and *P*-BINOL protons (Fig. S1†), confirming the proximity of these units in 2 and with *P*-BINOL adopting a transoid conformer (ESI, Section 8†). This overall conformation is also consistent with that obtained from conformer searches using the CREST code^28^ and the GFN2-xTB tight-binding DFT method^29^ for 2a, b (ESI, Section 8†). This approach is supported by the fact that the predicted lowest energy conformer of macrocycle 1a (Fig. S26†), for which we can obtain an X-ray crystal structure, agrees with that in the experimental X-ray crystal structure (Fig. 2b).

### Macrocycle chirality

Our next goal was to assess the impact of the twisted BINOL π-donor on the chirality of the PDI π-acceptor, focussing initially on macrocycle 2b. Importantly, while *P*-BINOL is configurationally stable at room temperature (Δ*G*^‡^ = 158 kJ mol^−1^),^30^ the twisted bis-alkyne PDI starting material is conformationally unstable (*i.e.*, chirality is dynamic with Δ*G*^‡^ < 39 kJ mol^−1^)^16,31^ and is racemic (1 : 1 *P* : *M*).^31*a*,*b*^ This provides the potential for two diastereomeric [1 + 1] macrocyclic products, homochiral *PP*-2b and heterochiral *PM*-2b. Indeed, upon analysis of the 2b crude mixture, we identified two *P*-BINOL–PDI macrocycles, formed in different yields. Since the relationship between *PP*-2b and *PM*-2b is diastereomeric, these compounds were separable by silica gel chromatography, exhibiting distinct ^1^H NMR spectra (Fig. 4b and c) and a single peak by chiral high-performance liquid chromatography (HPLC) (Fig. S23†). Therefore, our synthetic strategy enables us to obtain both *P* and *M* PDI atropisomers in high enantiopurity (>99%), without the need for chiral HPLC.^32^ By contrast, there is no stereoselectivity when the planar hydroquinone π-donor is used: macrocycle 1b is isolated as a racemic mixture and requires a chiral stationary phase to resolve the enantiomers *P*-1b and *M*-1b (Fig. S18 and S24†). Of course, upon swapping *P*-BINOL for *M*-BINOL in the macrocyclisation reaction, we obtain homo- and heterochiral macrocycles in the same diastereomeric ratio, with *MM*-2b and *MP*-2b having identical ^1^H NMR spectra to *PP*-2b and *PM*-2b respectively, due to their enantiomeric relationship (Fig. S2†).

To assign the chirality of the two *P*-BINOL–PDI macrocycle diastereomers, we used a combination of CD spectroscopy and (time-dependent) density functional theory ((TD)-DFT) calculations. In the latter we took the results of the above mentioned conformer search for the macrocycles using the GFN2-xTB tight-binding DFT method, and optimised the lowest-energy conformers with DFT using the B97-3c functional^33^ before calculating their UV-vis and CD spectra with (TD)-DFT using the ωB97x functional^34^ (ESI, Section 8†). Examination of the PDI-only region of the CD spectra (*λ* = 350–600 nm) reveals *PP*-2b and *PM*-2b exhibit opposite CD spectra, indicative of the enantiomeric relationship between the PDI units (Fig. 4d). With the aid of TD-DFT (Table S2†) we assign the negative signals in the visible part of the CD spectrum (*λ* > 400 nm) to the *P* atropisomer of PDI (*i.e.*, *PP*-2b), while the positive CD signals are from *M*-PDI (*i.e.*, *PM*-2b). Therefore, the higher yielding product is the heterochiral macrocycle (*PM*) and the lower yielding is homochiral (*PP*), giving a diastereomeric ratio of 4 : 1 (Fig. S5†). This is intriguing because previous work on diastereomeric PDI-based macrocycles has always found the major species to be homochiral.^22*a*–*c*,23^ Indeed, calculations suggests that *PP*-2b is the lowest energy macrocycle. A full GFN2-xTB tight-binding DFT conformer search performed using the heterochiral macrocycle *PM*-2b as the starting point returned only low-energy homochiral macrocycle conformers, thus *PM*-2b must lie at least 20–30 kJ mol^−1^ above that of *PP*-2b (ESI, Section 8†). As such, *PP*-2b is akin to the “thermodynamic product” of the macrocyclisation reaction while *PM*-2b, which is formed in the highest yield, is akin to the “kinetic product” (Fig. 1).

Interestingly, electronic circular dichroism is stronger throughout the PDI-only region for the heterochiral macrocycle relative to the homochiral macrocycle (Fig. 4d). This is indicative of a larger PDI twist angle^32*d*^ in *PM*-2b compared to *PP*-2b, a factor that will contribute to the latter being lower in energy. Indeed, inspection of the DFT optimised structures reveals that the PDI twist angle increases from 23° to 25° on moving from the homochiral (*PP*-2b) to the heterochiral (*PM*-2b) macrocycle structure and upon decreasing the linker length from hexyl (2b) to butyl (2a), which in both cases leads to an amplification of electronic circular dichroism (Fig. 4d, S15 and S29†).^32*d*^ Furthermore, the DFT optimised structure of macrocycle *PP*-2b shows an additional intramolecular CH–O hydrogen bond (*d* = 2.5 Å) between the triazole unit and the BINOL's O atom compared to *PM*-2b, which will also contribute to the former being lower in energy (Fig. S28†).§§Since this hydrogen bond involves a triazole heterocycle which is formed during the ring-closing of the macrocycle it is less likely to contribute towards differences in stability of the macrocyclisation transition state (Fig. S28†).

The reason that the heterochiral macrocycle does not convert to its lower energy homochiral analogue is because the CuAAC ring-closing reaction is irreversible, and, upon macrocyclisation, the PDI becomes configurationally stable (like *P*-BINOL). As such, there is no change to the ^1^H NMR or CD spectra of macrocycle 2 upon heating up to 180 °C, which is just below the isomerisation temperature of BINOL^35^ (*i.e.*, Δ*G*^‡^ > 158 kJ mol^−1^, Fig. S3 and S19†). The interconversion of macrocycle stereoisomers is inhibited because the macrocyclic cavities of 1 and 2 are too small for the PDI dye to somersault through,^22*b*,*d*^ therefore enabling us to isolate a configurationally stable heterochiral mono-PDI macrocycle for the first time. The stereoisomers of acyclic compounds 3–5 could not be resolved at room temperature because, as for all current acyclic disubstituted PDIs,^16,36^ the interconversion of bis-triazole PDI atropisomers is too fast (Δ*G*^‡^_int_ < 39 kJ mol^−1^, rate *k*_int_ > 6 × 10^5^ s^−1^, ESI Section 3.7†), making them “Class 1” atropsiomers under the LaPlante classification system.^31*c*^

### Curtin–Hammett analysis

**Fig. 5:**
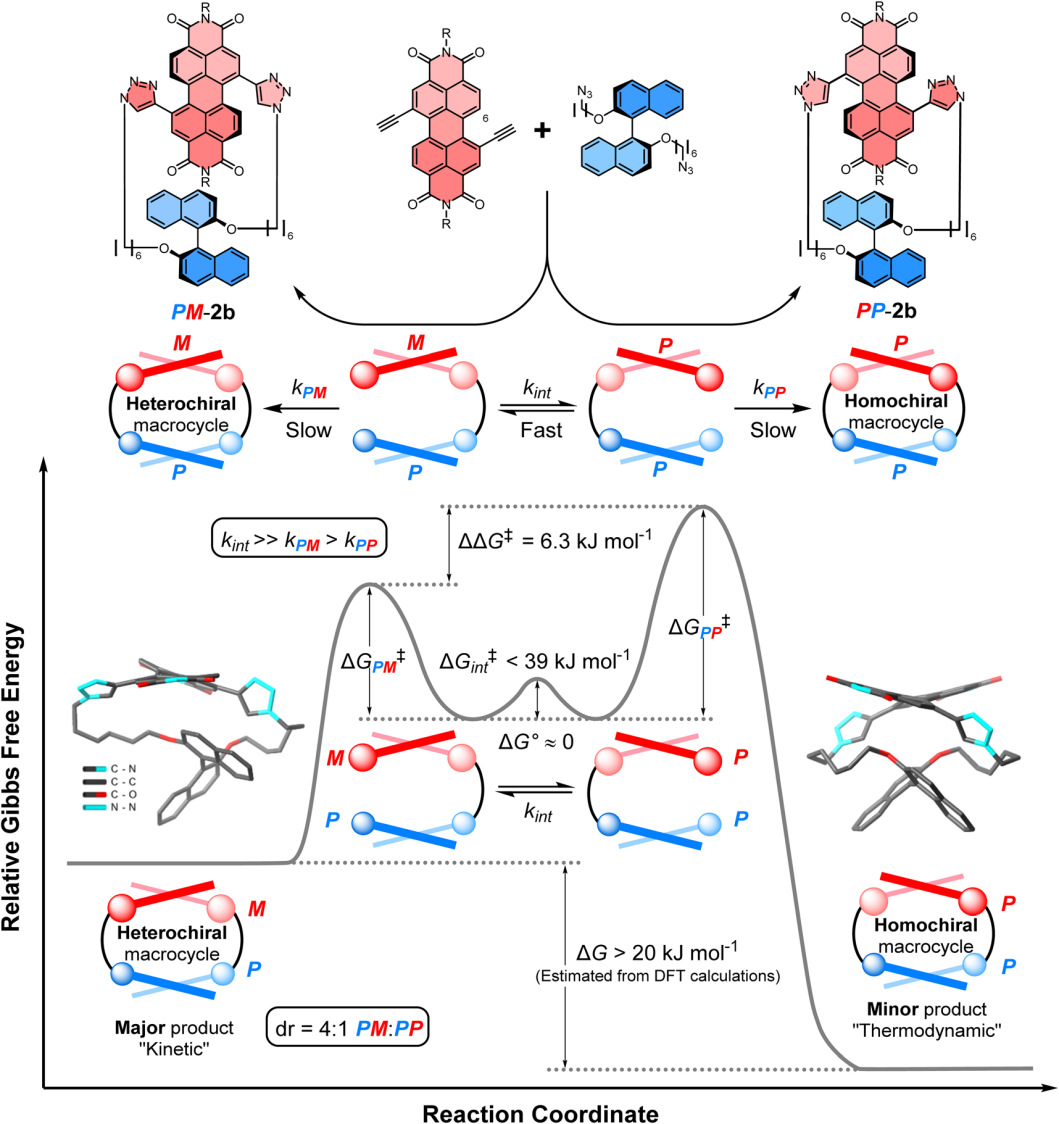
The energy profile diagram for the diastereoselective synthesis of macrocycle *PM*-2b under Curtin–Hammett control. Values for the free energies ΔΔ*G*^‡^, Δ*G*° and Δ*G*^‡^_int_ have been determined experimentally, while the free energy difference between macrocycles *PM*-2b and *PP*-2b (Δ*G*) has been estimated computationally using the DFT (B973c) optimized geometries of the likely lowest energy conformer of macrocycles *PM*-2b and *PP*-2b, as found in the tight-binding DFT (GFN2-xTB) conformer searches (ESI, Section 8†). These computational structures are displayed on the diagram, with hydrogen atoms and the 2,6 diisopropylphenyl imide substituents removed for clarity.

**Fig. 6:**
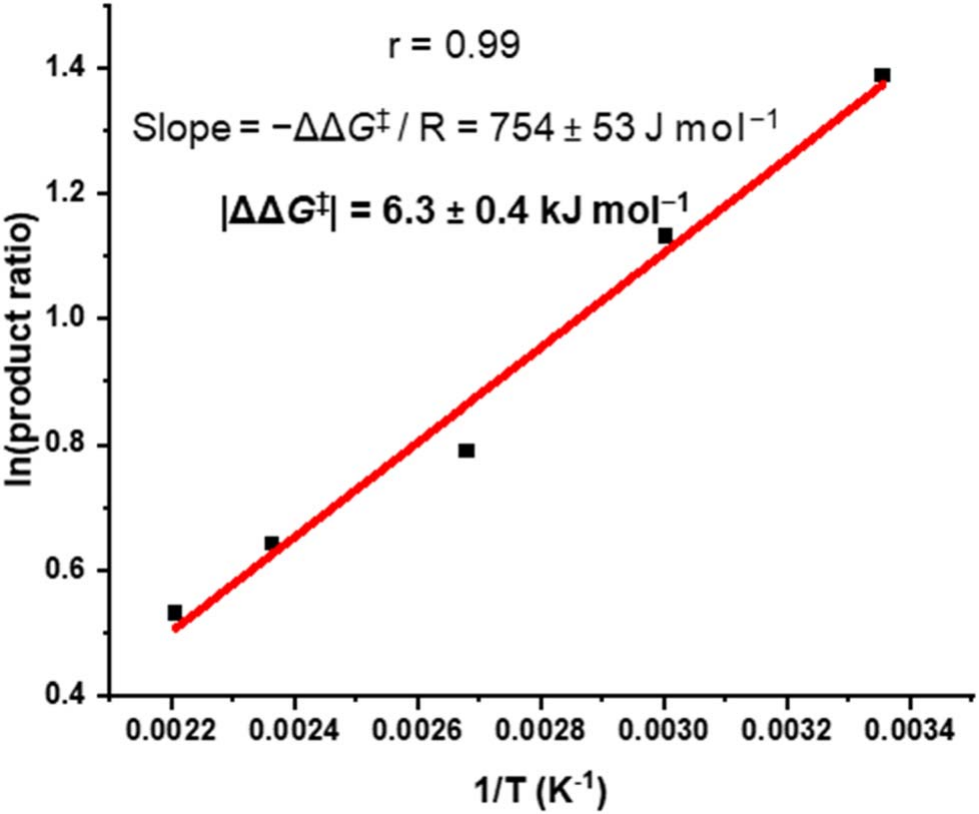
Curtin–Hammett plot of macrocycle 2b, where 2b was synthesised at a range of temperatures (*T*) and the product ratio ([*PM*-2b]/[*PP*-2b]) determined by ^1^H NMR spectroscopy.

We sought to understand the origin of stereoselectivity in the synthesis of macrocycle 2b using the Curtin–Hammett principle. Firstly, it is important to note that, in a macrocyclisation reaction, the rate determining step is typically the one that forms the ring,^37^ which for 1, 2 is the second ring-closing CuAAC ‘click’ reaction. For macrocycle 2, this reaction fulfils the criteria for Curtin–Hammett control because, while the CuAAC chemistry is irreversible and the macrocycle products are configurationally stable, the diastereomers of the mono-clicked macrocycle intermediate (*PP* and *PM*, Fig. 5) are in thermodynamic equilibrium and able to rapidly interconvert (Δ*G*^‡^_int_ < 39 kJ mol^−1^), being acyclic disubstituted PDIs (*vide supra*). Indeed, the diastereomers of acyclic BINOL–PDI–BINOL control compound 4 (*PMP* and *PPP*) could not be isolated or observed by ^1^H NMR spectroscopy due to their rapid exchange (see ESI Section 3.7†).^38^ The Curtin–Hammett principle states that the reaction outcome is solely dependent on the difference in transition state energies of the products (ΔΔ*G*^‡^, Fig. 1). By synthesising macrocycle 2b at different temperatures, we showed that diastereoselectivity decreases upon increasing the macrocylisation reaction temperature (Fig. S9†) and hence calculated the difference in free energy between the transition states of the *PM* and *PP* macrocyclic products, |ΔΔ*G*^‡^| = 6.3 ± 0.4 kJ mol^−1^ (Fig. 6). This ΔΔ*G*^‡^ is dependent on the relative free energy of the interconverting intermediate (Δ*G*°) and the relative activation energies (Δ*G*^‡^_*PP*_ − Δ*G*^‡^_*PM*_). For 2b, Δ*G*° ≈ 0, since the acyclic BINOL–PDI–BINOL compound 4 does not exhibit a CD spectrum in the PDI-only region (Fig. 4d), which indicates that the mono-clicked *PP* and *PM* diastereomeric intermediates of 2b are very close in energy prior to macrocyclisation. Therefore, the difference in transition state energies ΔΔ*G*^‡^ (and hence the kinetic resolution of 2b) does not occur from any inherent stability of the heterochiral mono-clicked intermediate over the homochiral intermediate (Δ*G*°), and instead arises purely on the difference in activation energies (Δ*G*^‡^_*PP*_ − Δ*G*^‡^_*PM*_, Fig. 5).

To investigate the potential steric and electronic factors behind this difference in activation energies, we prepared two further PDI–BINOL macrocycles with shorter (*n*-butyl, 2a) and longer (*n*-octyl, 2c) strap lengths in comparison to 2b (*n*-hexyl).¶¶Unfortunately, the use of shorter or longer linkers outside of this window did not lead to the formation of stable [1 + 1] macrocycles in an isolable yield. Interestingly, neither of these macrocycles exhibited any diastereoselectivity (*i.e.*, *PP*-2a,c : *PM*-2a,c = 1 : 1). Therefore, stabilisation of the heterochiral transition state is unique to macrocycle 2b, suggesting that the trend in diastereoselectivity does not correlate with the change in ring strain that would be anticipated upon changing the linker length. As such, in light of the evidence for intramolecular π–π stacking in our macrocycles (*vide supra*), we also sought to identify any connection between diastereoselectivity and the potential differences in electronic properties.

**Fig. 7:**
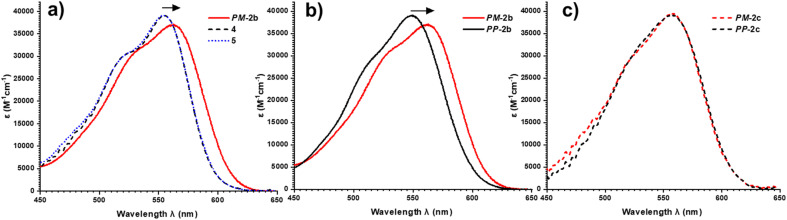
UV-vis spectra (CHCl_3_, 298 K, 26 μM) of: (a) macrocycle *PM*-2b, which is bathochromically shifted relative to acyclic bis-triazole PDIs 4, 5; (b) heterochiral macrocycle *PM*-2b, which is bathochromically shifted relative to homochiral macrocycle *PP*-2b; (c) heterochiral and homochiral macrocycles *PM*-2c and *PP*-2c, with no relative bathochromic shift.

To investigate this, we measured the UV-vis absorption spectra of the diastereomers of macrocycles 2a–c and the acyclic control compounds 4 and 5. Here it is important to clarify that π–π interactions primarily consist of electrostatic and van der Waals contributions,^39^ with the former readily probed by UV-vis spectroscopy since red-shifted charge transfer absorbances can be a consequence of donor–acceptor interactions.^18*b*,40^ Relative to acyclic bis-triazole PDIs, the main PDI absorption band (S_0_–S_1_) of *PM*-2b is significantly red-shifted (Δ*λ* = 10 nm), indicative of new donor–acceptor π–π interactions in this heterochiral macrocycle (Fig. 7a).||||We note that the absorption spectra of 2b follow the Beer–Lambert law (Fig. S21†), indicating that the π–π donor–acceptor interaction in *PM*-2b is intramolecular in origin. However, the absorption spectra of *PP*-2b is relatively unchanged (Fig. 7b), indicating any donor–acceptor π–π interactions in the homochiral macrocycle are significantly weaker.**We note that improved π-conjugation arising from increased chromophore planarity would also lead to red-shifted UV-vis spectra, yet this is not consistent with the computational structures which indicate that the bis-triazole PDI is more twisted in *PM*-2b relative to *PP*-2b (Fig. S29†). The importance of donor–acceptor π–π interactions is also demonstrated by UV-vis analysis of the shorter- (2a) and longer-linked (2c) macrocycles. With both macrocycles exhibiting no diastereoselectivity (Fig. S4 and S8†), it is notable that the diastereomers *PP*-2c and *PM*-2c have similarly red-shifted PDI absorption bands (ΔΔ*λ* ≈ 0 nm, Fig. 7c), as do diastereomers *PP*-2a and *PM*-2a (ΔΔ*λ* = 1 nm, Fig. S22†).††††Relative to 5, the acyclic control compound 4 does not exhibit a red-shifted PDI absorption band (Fig. 7a), which explains the absence of a CD spectrum in the PDI-only region of 4 (Fig. 4d). Moreover, this trend is in line with our evidence for intramolecular aromatic interactions by ^1^H NMR spectroscopy, for which CH–π as well as π–π interactions may contribute. Indicative of π–π interactions, the heterochiral macrocycle *PM*-2b exhibits a larger downfield shift of PDI protons relative to homochiral *PP*-2b (for H_c_, Δ*δ* = 8.47 *vs.* 7.88 ppm, ΔΔ*δ* = 0.59 ppm, Fig. 4a–c), while the difference between diastereomers is much smaller for macrocycles 2a and 2c (ΔΔ*δ* = 0.1 and 0.07 ppm respectively, ESI Section 1†). Indicative of BINOL–PDI CH–π interactions,^41^ relative to acyclic *P*-BINOL, *PM*-2b also exhibits a larger average upfield shift of BINOL protons H_3–5_, compared to *PP*-2b (Δ*δ* = 0.7 *vs.* 0.4 ppm, Fig. S12†). These findings are supported by the computational structures, which show that BINOL–PDI π–π and CH–π distances are overall shorter, and in the latter case also more numerous, in the heterochiral *vs.* homochiral structures of macrocycle 2b (Fig. S28†).

Overall, diastereomers *PM*-2b and *PP*-2b are the only pair of macrocyclic products to show significantly different PDI absorption bands (ΔΔ*λ* = 14 nm) and have the largest discrepancy between their ^1^H NMR PDI aromatic protons (ΔΔ*δ* = 0.59 ppm) and are the only pair to show diastereoselectivity. Taken together, these results show that the geometry of macrocycle 2b is unique in facilitating stronger non-covalent interactions, including π–π and CH–π interactions, in *PM*-2b relative to *PP*-2b. These stronger aromatic interactions provide a potential kinetic template effect during the formation of *PM*-2b and help to rationalise why the heterochiral transition state of 2b is stabilised relative to the homochiral transition state in the Curtin–Hammett-controlled macrocycle synthesis (Fig. 5). Indeed, a ΔΔ*G*^‡^ = −6.3 kJ mol^−1^ is of the order of the binding free energy of donor–acceptor pairs such as dialkoxy naphthalene–naphthalene diimide in a chlorinated solvent (Δ*G* = −9.3 kJ mol^−1^),^42^ which supports our theory of the importance of aromatic-based non-covalent interactions. To provide further evidence for supramolecular templation, we repeated the synthesis of macrocycle 2b in competitive solvents for π–π donor–acceptor interactions and, as expected, found that the 4 : 1 *PP* : *PP* ratio decreases both upon increasing solvent polarity (dr = 3 : 1 in DMF, Fig. S7†), which tempers electrostatic contributions, and upon the addition of an aromatic solvent (dr = 3 : 1 in 1 : 1 toluene : DCM, Fig. S6†).‡‡‡‡The solubility of starting materials dictated the need for a 1 : 1 toluene:DCM solvent mixture.

## Conclusions

We have used the Curtin–Hammett principle to investigate a chiral macrocyclisation reaction, revealing the potential for intramolecular aromatic non-covalent interactions to direct the outcome of a dynamic kinetic resolution, in this case the diastereoselective synthesis of a macrocycle containing twisted aromatic units (2b). A racemic and dynamically chiral PDI dye (1 : 1 *P* : *M*) is made configurationally stable by its strapping with an enantiopure *P*-BINOL derivative under irreversible reaction conditions. Therefore, in contrast to previous diastereomeric PDI-based macrocycles,^22*a*–*c*,23^ both the macrocyclisation reaction and products are under kinetic control, enabling the facile isolation of heterochiral and homochiral macrocycles in high enantiopurities. Notably, instead of a 1 : 1 mixture of macrocycle diastereomers,^19*a*,43^ there is diastereoselectivity for the heterochiral (*PM*-2b) over homochiral (*PP*-2b) product (dr = 4 : 1). From Curtin–Hammett analysis, the origin of this diastereoselectivity is the lower transition state energy of the hetero- *vs.* homo-chiral macrocycle (ΔΔ*G*^‡^ = 6.3 kJ mol^−1^), with experimental and computational studies revealing that stabilising π–π and CH–π interactions are predominant in *PM*-2b, indicative of a kinetic template effect.^9*a*^

While homochirality appears to dominate in dimeric assemblies of PDIs,^22*a*,23,32*a*,*d*^ a preference for heterochiral π–π stacking has recently been reported in PDI-based complexes of polycyclic aromatic hydrocarbons^19*e*^ and a BINOL-perylene cyclophane^22*c*^ where, as in our work here, it is notable that the π-conjugated units in the π–π stack are different. Overall, this suggests that the selection of equivalent or distinct aromatic units is an important consideration when designing new templated syntheses of homo- or hetero-chiral π-conjugated materials. The use of aromatic non-covalent interactions to direct the synthesis of such atropisomeric π-conjugated materials^22*a*,44^ under irreversible conditions is desirable for preparing preorganised chiral receptors^45^ or persistent chiroptical materials^3^ or, as found here, for tuning selectivity for a chiral product that may not be favoured under thermodynamically controlled conditions. This is leading us to develop the stereoselective synthesis of new supramolecular architectures for chiral sensing and the emission/detection of circularly polarised light.

## Data availability

All synthetic method descriptions, product characterisation data, spectroscopic data and other supplementary figures and tables (NMR spectra, UV-vis spectra, circular dichroism spectra, chiral HPLC chromatograms, *etc.*), crystallographic information, and computational data can be found in the ESI.† The relevant computational structures are available in the folder named “Hetero_homochiral_macrocycles_structures”.

## Author contributions

A. Y. and T. A. B. conceptualisation, writing and editing; A. Y. investigation and analysis of all synthetic and spectroscopic data (NMR, UV-vis, CD, chiral HPLC), visualisation (figures), and writing of ESI;† M. A. Z. performed all computational studies and wrote the computational sections; G. R. F. O. processed and solved all crystallographic data, and wrote the crystallographic sections; J. H. R. crystallographic data collection; and T. A. B. writing of original manuscript draft, supervision and funding.

## Conflicts of interest

There are no conflicts to declare.

## Supplementary Material

SC-015-D3SC05715A-s001

SC-015-D3SC05715A-s002

SC-015-D3SC05715A-s003
