# Supplementary material for: Investigating the diastereoselective synthesis of a macrocycle under Curtin–Hammett control
Source: Chem Sci. 2024 Mar 5;15(15):5516–24. doi: 10.1039/d3sc05715a (PMC11023033; doi:10.1039/d3sc05715a)
Supplement: SC-015-D3SC05715A-s002 [file SC-015-D3SC05715A-s002.pdf]

Electronic Supplementary Material (ESI) for Chemical Science.
This journal is © The Royal Society of Chemistry 2024

Supporting Information for:

Investigating the Diastereoselective Synthesis of a Macrocycle under Curtin-Hammett Control

Angus Yeung,[a] Martijn A. Zwijnenburg,[b] Georgia R. F. Orton,[a] Jennifer Robertson[a] and Timothy A. Barendt*[a]

[a] School of Chemistry, University of Birmingham, Edgbaston, Birmingham, UK, B15 2TT
[b] Department of Chemistry, University College London, 20 Gordon Street, London, UK, WC1H 0AJ
*t.a.barendt@bham.ac.uk

Table of Contents

1. Materials and Techniques S2
2. Synthesis and Characterisation S3
3. Further NMR Spectroscopy Experiments S47
4. Circular Dichroism Studies S65
5. UV-vis Spectroscopy S71
6. Chiral HPLC S73
7. X-ray Crystallography S75
8. Density Functional Theory Calculations S77
9. References S84

1. Materials and Techniques

All commercial solvents and reagents were used as purchased, unless otherwise stated. Anhydrous solvents were degassed with N_2 and dried using an Innovative Technology PureSolv MD 5 solvent purification system. $Cu(MeCN)_4{\cdot}PF_6$ was stored in a desiccator. 1-Azidohexane,[1] tris((1-benzyl-4-triazolyl)methyl)amine (TBTA),[2] and tris-hydroxypropyltriazolylmethylamine (THPTA)[3] were prepared following literature procedures. Water was distilled and microfiltered using an ELGA DV 35 Purelab water purification system. Chromatography was undertaken using silica gel (particle size: 40-63 μm) or preparative TLC plates (20 × 20 cm, 1 cm silica thickness).

All NMR spectra (1D: ^{1}H, ^{13}C; 2D: $^{1}H-^{1}H$ COSY, $^{1}H-^{1}H$ NOSEY, $^{1}H-^{13}C$ HSQC, and $^{1}H-^{13}C$ HMBC) were recorded at 298 K using Bruker AVIII400 (400 MHz), Bruker AV NEO 400 (400 MHz), or Bruker AV NEO 500 (500 MHz) spectrometers. These NMR spectra were also used for assignment of peaks in 1D ^{1}H NMR spectra. Mass spectra were recorded using a Waters Synapt G2-S mass spectrometer or a Waters Xevo G2-XS mass spectrometer for high resolution MS-ESI or MS-ASAP.

2. Synthesis and Characterisation

Compounds **6-8** were isolated as a 2:1 inseparable mixture of 1,7 and 1,6 regioisomers.[4] Macrocycles **1-2** were isolated as the pure 1,7 regioisomer since removal of the 1,6 regioisomer was possible by silica gel column chromatography at this stage. Acyclic compounds **3-5** were initially isolated as a regioisomeric mixture and purified by HPLC. HPLC purifications were performed using a COSMOSIL Buckyprep Packed Column (10.0 mm I.D. × 250 mm) on an Agilent 1290 Infinity analytical HPLC instrument at a flow rate of 3 mL/minute, with a detection wavelength of 500 nm. The eluents for each chromatogram are specified in the text.

Note that for clarity, the chiral nomenclature of BINOL and related compounds have replaced ***R***- and ***S***- with ***M***- and ***P***- labels respectively.

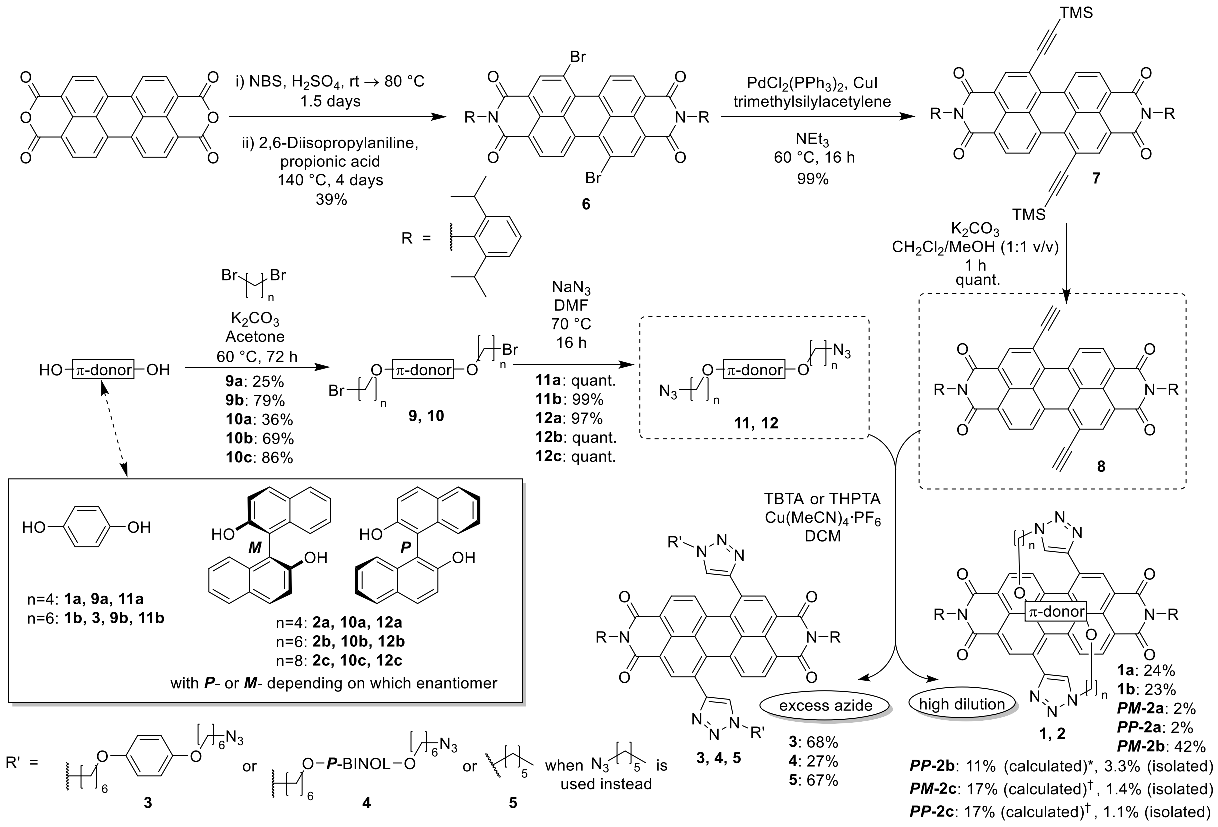

*11% yield of ***PP*-2b** is calculated from ^{1}H NMR spectroscopy of crude mixture.

†**2c** was first isolated as a 1:1 mixture of ***PM*-2c** and ***PP*-2c** (ratio of 1:1 is also confirmed by ^{1}H NMR spectroscopy of crude mixture).

Scheme S1: Multistep synthesis of PDI compounds **1-11**.1-Azidohexane,[1] TBTA,[2] and THPTA[3] were prepared following literature procedures.

General procedure for compounds 1–5: Copper(I)-catalysed Azide-Alkyne Cycloaddition (CuAAC)

Alkyne and organic azide were dissolved in CH_2Cl_2 and degassed by bubbling a stream of N_2 for 15 min. TBTA or THPTA and $Cu(MeCN)_4{\cdot}PF_6$ was then sequentially added, and the resulting mixture was left to stir at rt under an atmosphere of N_2 for a specified time, or until reaching completion as monitored by TLC. After which, the reaction mixture concentrated *in vacuo* and purified *via* flash column chromatography.

1,4-Di(4-butoxy)benzene PDI macrocycle 1a

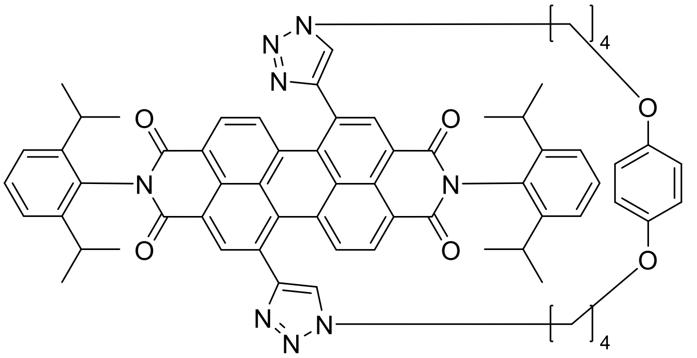

The general procedure above was followed with dialkynyl PDI **8** (120 mg, 0.158 mmol, 1 eq), 1,4-di(4-azidobutoxy)benzene **11a** (48.1 mg, 0.158 mmol, 1 eq), CH_2Cl_2 (260 mL), TBTA (33.5 mg, 0.0633 mmol, 0.4 eq) and $Cu(MeCN)_4{\cdot}PF_6$ (23.6 mg, 0.0633 mmol, 0.4 eq). The reaction was completed after four days and purified by flash column chromatography (SiO_2, 0.4:100 MeOH:CH_2Cl_2) to give macrocycle **1a** as a purple solid (27 mg, 24%).

^{1}H NMR ($CDCl_3$, 400 MHz) δ 8.84 (2H, s, Ar*H* Ortho PDI), 8.38 (2H, d, *J* = 8.0 Hz, Ar*H* Ortho PDI), 8.27 (2H, d, *J* = 8.0 Hz, Ar*H* Bay PDI), 8.01 (2H, s, C*H* triazole), 7.50 (2H, t, *J* = 7.7 Hz, iPr$_2$-Ar*H*), 7.35 (4H, app dd, *J* = 7.7, iPr$_2$-Ar*H*), 6.11 (4H, s, Ar*H* oxybenzene), 4.63 (2H, ddd, *J* = 14.0, 8.5, 3.7 Hz, O(CH$_2$)$_3$C*H*$_A$H$_B$N), 4.54 (2H, ddd, *J* = 14.0, 6.8, 3.7 Hz, O(CH$_2$)$_3$CH$_A$*H*$_B$N), 3.36 – 3.23 (4H, m, OC*H*$_2$(CH$_2$)$_3$N), 2.85 (2H, hept, *J* = 6.9 Hz, C*H* iPr), 2.71 (2H, hept, *J* = 6.7 Hz, C*H* iPr), 2.23 – 2.02 (4H, m, OCH$_2$C*H*$_2$(CH$_2$)$_2$N), 1.77 – 1.55 (4H, m, O(CH$_2$)$_2$C*H*$_2$CH$_2$N), 1.24 (1H, d, *J* = 6.9 Hz, CH(C*H*$_3$)$_2$ iPr), 1.22 (2H, d, *J* = 6.9 Hz, CH(C*H*$_3$)$_2$ iPr), 1.16 (3H, d, *J* = 6.7 Hz, CH(C*H*$_3$)$_2$ iPr), 1.24 (1H, d, *J* = 6.7 Hz, CH(C*H*$_3$)$_2$ iPr)

^{13}C NMR (101 MHz, $CDCl_3$) δ 163.5, 163.3, 152.3, 147.5, 147.0, 145.8, 145.6, 135.0, 134.6, 133.4, 130.5, 130.1, 129.9, 129.7, 129.5, 129.4, 128.5, 124.4, 124.1, 123.5, 122.8, 122.4, 114.7, 77.5, 77.2, 76.8, 66.3, 50.3, 29.8, 29.5, 29.3, 25.8, 24.2, 24.2, 24.1, 24.0.

MS (ESI): m/z calc. for $C_{66}H_{63}N_8O_6$ $[M+H]^+$: 1063.4871; found 1063.4869

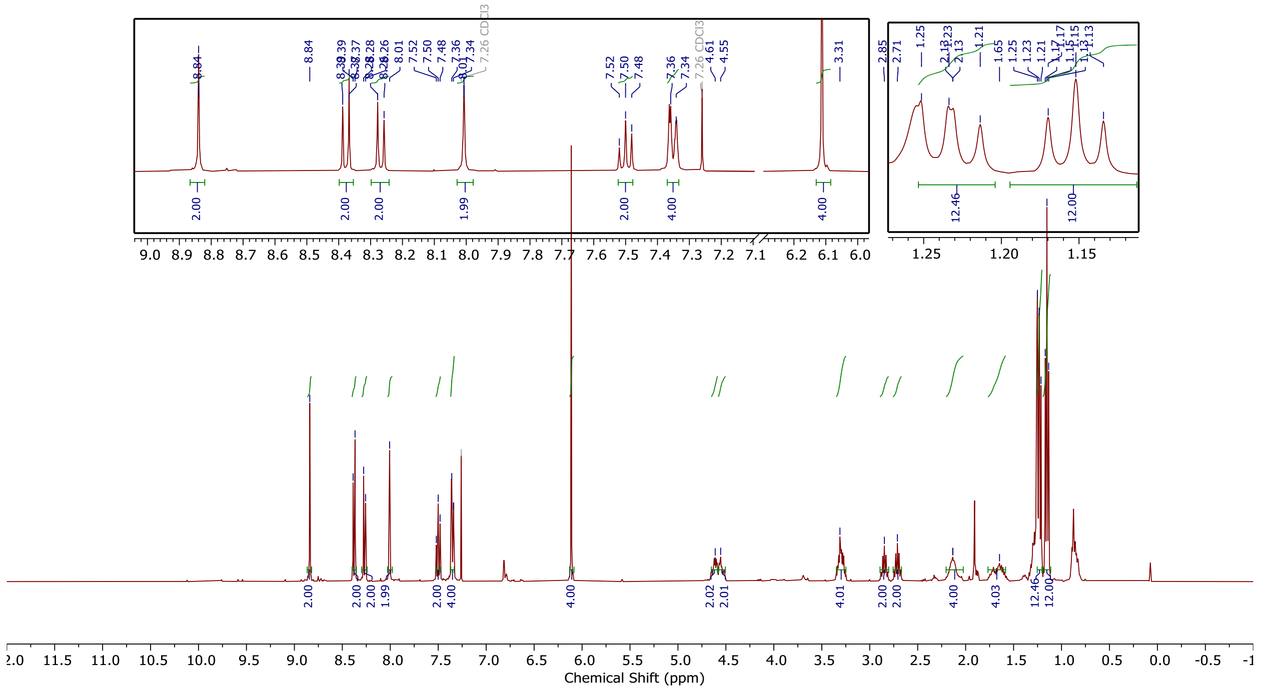

^{1}H NMR spectrum of **1a** (400 MHz, $CDCl_3$).

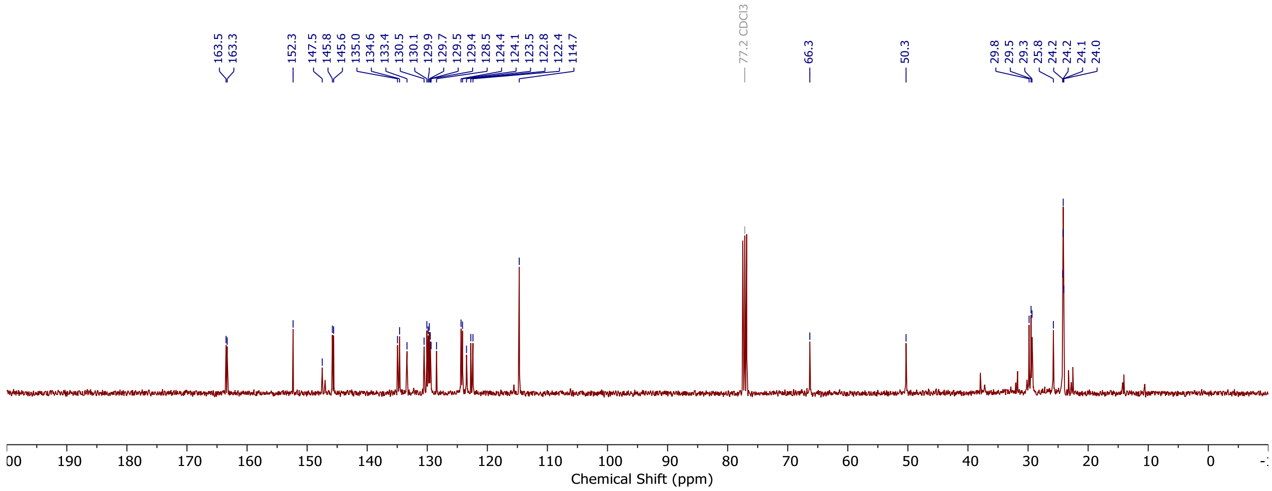

^{13}C NMR spectrum of **1a** (101 MHz, $CDCl_3$).

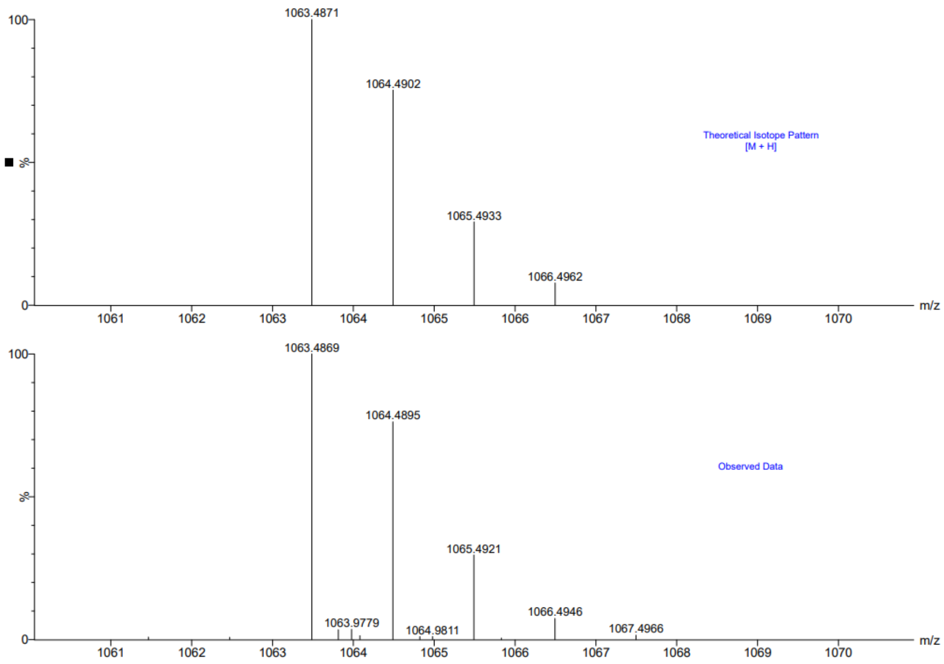

Calculated (top) and observed (bottom) ESI MS data for compound **1a**.

1,4-Di(6-hexoxy)benzene PDI macrocycle 1b

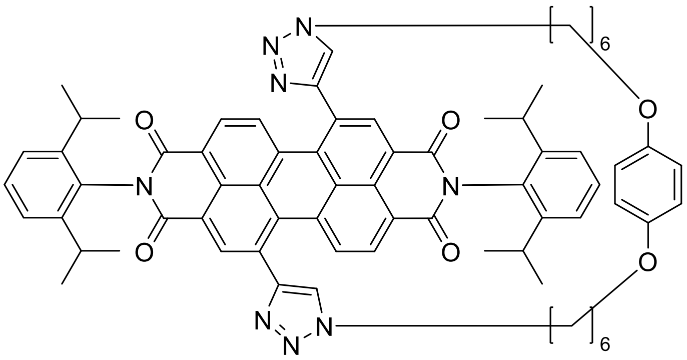

The general procedure above was followed with dialkynyl PDI **8** (150 mg, 0.198 mmol, 1 eq), 1,4-di(6-azidohexoxy)benzene **11b** (75.0 mg, 0.198 mmol, 1 eq), CH_2Cl_2 (600 mL), TBTA (45 mg, 79.1 µmol, 0.4 eq) and $Cu(MeCN)_4{\cdot}PF_6$ (30 mg, 79.1 µmol, 0.4 eq). The reaction was completed after four days and purified by flash column chromatography (SiO_2, 0.4:100 MeOH:CH_2Cl_2) to give macrocycle **1b** a purple solid (33.7 mg, 23%).

^{1}H NMR (400 MHz, $CDCl_3$) δ 8.74 (s, 2H, Ar*H* Ortho PDI), 8.37 (d, *J* = 8.1 Hz, 2H, Ar*H* Ortho PDI), 8.15 (d, *J* = 8.1 Hz, 2H, Ar*H* Bay PDI), 7.65 (s, 2H, C*H* triazole), 7.49 (t, *J* = 7.8 Hz, 2H, iPr_2-Ar*H*), 7.37 – 7.31 (m, 4H, iPr_2-Ar*H*), 6.19 (s, 4H, Ar*H* oxybenzene), 4.64 (ddd, *J* = 13.9, 7.5, 3.9 Hz, 2H, NCH_AH_B), 4.48 (ddd, *J* = 13.9, 8.1, 3.9 Hz, 2H, NCH_AH_B), 3.60 – 3.37 (m, 4H, OCH_2), 2.79 (p, *J* = 6.8 Hz, 2H, C*H* iPr), 2.68 (d, *J* = 6.9 Hz, 2H, C*H* iPr), 2.22 – 2.04 (m, 2H $NCH_2CH_AH_B$), 2.04 – 1.87 (m, 2H, $NCH_2CH_AH_B$), 1.72 – 1.25 (m, 12H, CH_2), 1.21 (d, *J* = 6.8 Hz, 6H, $CH(CH_3)_2$ iPr), 1.18 (d, *J* = 6.8 Hz, 6H, $CH(CH_3)_2$ iPr), 1.15 (d, *J* = 6.9 Hz, 6H, $CH(CH_3)_2$ iPr), 1.13 (d, *J* = 6.9 Hz, 6H, $CH(CH_3)_2$ iPr).

^{13}C NMR (101 MHz, $CDCl_3$) δ 163.5, 163.2, 152.6, 147.5, 145.8, 145.7, 135.8, 135.1, 133.8, 130.5, 129.9, 129.7, 129.5, 129.3, 129.0, 124.4, 124.2, 122.7, 122.7, 122.2, 114.7, 67.4, 49.9, 29.3, 29.3, 29.1, 28.2, 24.6, 24.4, 24.3, 24.3, 24.2, 24.1.

MS (ESI): m/z calc. for $C_{70}H_{71}N_8O_6$ $[M+H]^+$: 1119.5497; found 1119.5502

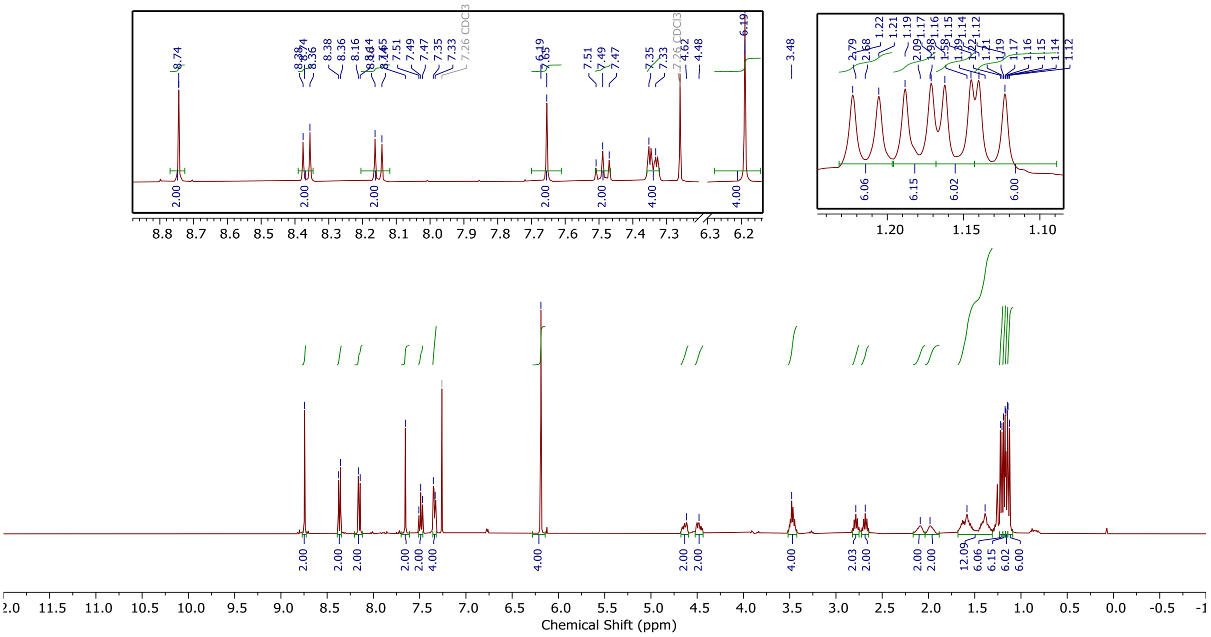

^{1}H NMR spectrum of **1b** (400 MHz, $CDCl_3$).

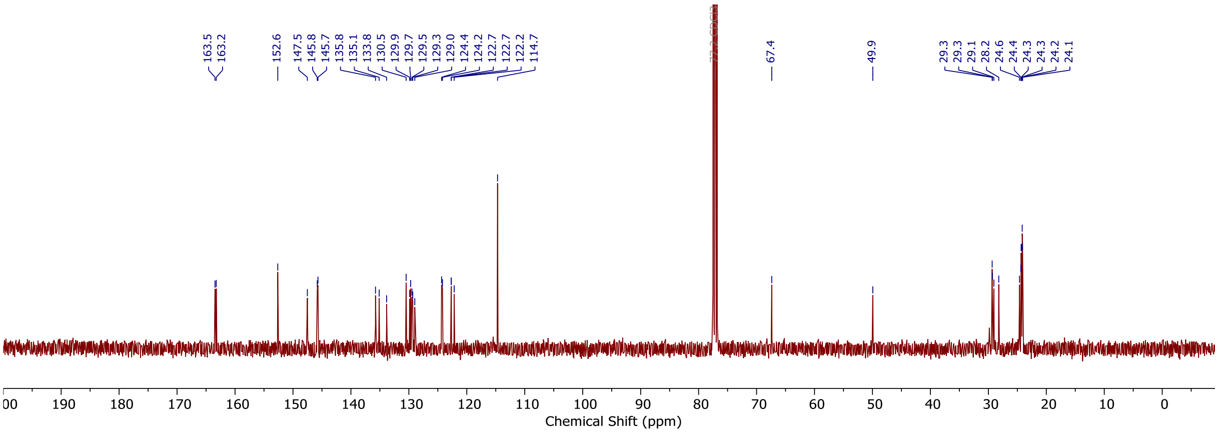

^{13}C NMR spectrum of **1b** (101 MHz, $CDCl_3$).

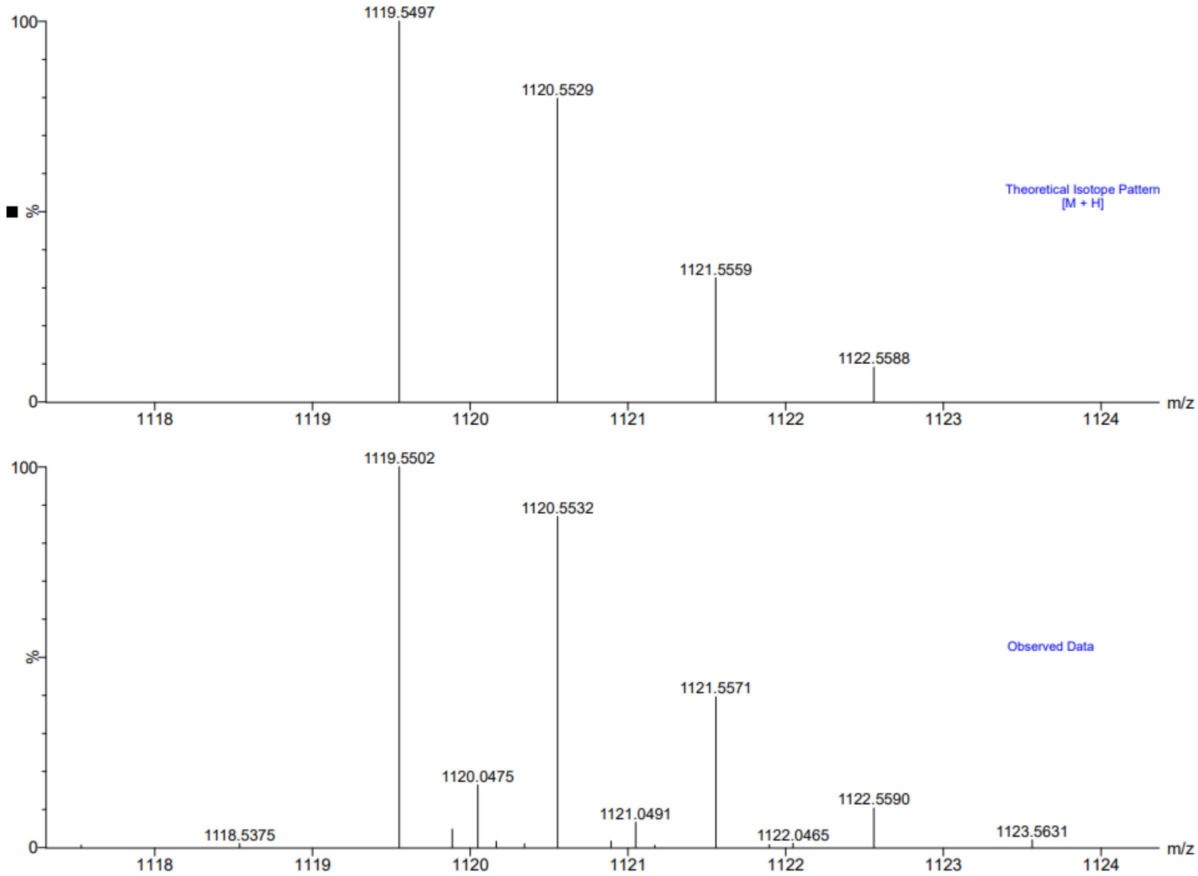

Calculated (top) and observed (bottom) ESI MS data for compound **1b**.

***P*-BINOL butyl PDI macrocycle 2a**

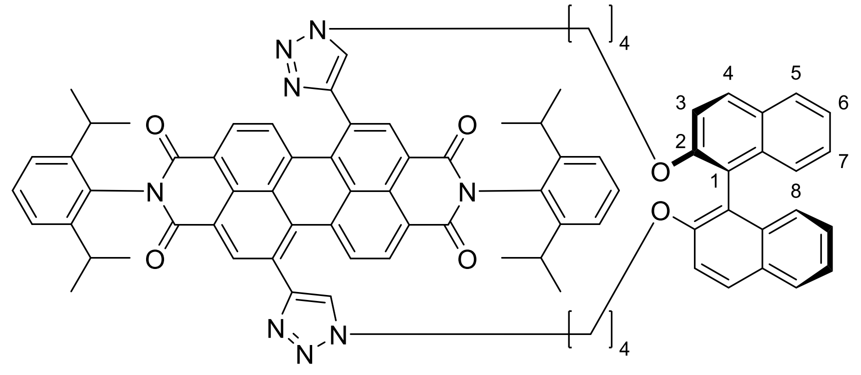

The general procedure above followed with dialkynyl PDI **8** (100 mg, 0.132 mmol, 1 eq), ***P***-2,2'-bis((4-azidobutyl)oxy)-1,1'-binaphthalene **12a** (63.3 mg, 0.132 mmol, 1 eq), CH_2Cl_2 (600 mL), THPTA (23 mg, 52.7 µmol, 0.4 eq) and $Cu(MeCN)_4{\cdot}PF_6$ (20 mg, 52.7 µmol, 0.4 eq). The reaction was completed after six days and purified by flash column chromatography (SiO_2, 0.4:100 → 0.6:100 MeOH:CH_2Cl_2), then by preparative TLC (SiO_2, 1:100 MeOH:CH_2Cl_2) to give the two diastereomers of the macrocycle ***PM*-2a** and ***PP*-2a** as purple solids (2.0 mg, 2%; 2.0 mg, 2%).

Diastereomer 1, ***PM*-2a**:

^{1}H NMR (500 MHz, $CDCl_3$) δ 9.36 (s, 2H, Ar*H* Ortho PDI), 8.65 (d, *J* = 8.0 Hz, 2H, Ar*H* Ortho PDI), 8.48 (d, *J* = 8.0 Hz, 2H, Ar*H* Bay PDI), 7.55 – 7.48 (m, 4H, iPr_2-Ar*H*, C(5)*H*), 7.36 (app ddd, *J* = 11.8, 7.8, 1.4 Hz, 4H, iPr_2-Ar*H*), 7.30 – 7.26 (obs m, 2H, C(6)*H*), 7.24 (s, 2H, C*H* triazole), 7.18 (ddd, *J* = 8.4, 6.8, 1.3 Hz, 2H, C(7)*H*), 7.10 (d, *J* = 9.0 Hz, 2H, C(4)*H*), 7.07 (dd, *J* = 8.4, 1.1 Hz, 2H, C(8)*H*), 6.77 (d, *J* = 9.0 Hz, 2H, C(3)*H*), 4.54 (ddd, *J* = 13.9, 7.9, 3.5 Hz, 2H, NCH_AH_B), 3.83 (ddd, *J* = 13.9, 7.5, 3.1 Hz, 2H, NCH_AH_B), 3.73 (ddd, *J* = 9.7, 7.7, 3.6 Hz, 2H, OCH_AH_B), 3.52 – 3.41 (m, 2H, OCH_AH_B), 2.81 (dh, *J* = 13.7, 6.8 Hz, 4H, C*H* iPr), 1.22 (d, *J* = 6.9 Hz, 6H, $CH(CH_3)_2$ iPr), 1.21 (d, *J* = 6.9 Hz, 6H, $CH(CH_3)_2$ iPr), 1.19 (d, *J* = 6.9 Hz, 6H, $CH(CH_3)_2$ iPr), 1.12 (d, *J* = 6.9 Hz, 6H, $CH(CH_3)_2$ iPr), 1.39 – 0.98 (m, 4H, CH_2).

^{13}C NMR (126 MHz, $CDCl_3$) δ 163.5, 163.0, 153.8, 147.2, 145.8, 145.4, 134.8, 134.4, 133.7, 132.5, 130.4, 129.8, 128.9, 128.6, 127.5, 126.5, 125.4, 124.3, 124.2, 124.1, 123.2, 122.8, 122.3, 120.3, 115.5, 69.2, 50.5, 29.7, 29.4, 29.3, 26.1, 24.1, 24.1, 24.0, 24.0.

MS (ESI): m/z calc. for $C_{80}H_{71}N_8O_6$ $[M+H]^+$: 1239.5497; found 1239.5510

Diastereomer 2, ***PP*-2a**:

^{1}H NMR (500 MHz, $CDCl_3$) δ 9.17 (s, 2H, Ar*H* Ortho PDI), 8.43 (d, *J* = 8.0 Hz, 2H, Ar*H* Ortho PDI), 8.38 (d, *J* = 8.0 Hz, 2H, Ar*H* Bay PDI), 7.69 (dd, *J* = 8.1, 1.4 Hz, 2H, C(5)*H*), 7.64 (d, *J* = 9.0 Hz, 2H, C(4)*H*), 7.51 (t, *J* = 7.8 Hz, 2H, iPr_2-Ar*H*), 7.36 (app ddd, *J* = 7.8, 4.4, 1.4 Hz, 4H, iPr_2-Ar*H*), 7.29 – 7.23 (obs m, 2H, C(6)*H*), 7.18 (ddd, *J* = 8.4, 6.7, 1.4 Hz, 2H, C(7)*H*), 7.15 (s, 2H, C*H* triazole), 7.06 (dd, *J* = 8.4, 1.1 Hz, 2H, C(8)*H*), 7.01 (d, *J* = 9.0 Hz, 2H, C(3)*H*), 4.50 (dt, *J* = 14.2, 4.7 Hz, 2H, NCH_AH_B), 4.12 (ddd, *J* = 14.2, 10.6, 3.6 Hz, 2H, NCH_AH_B), 3.84 (td, *J* = 9.5, 5.3 Hz, 2H, OCH_AH_B), 3.54 (ddd, *J* = 9.5, 8.4, 6.6 Hz, 2H, OCH_AH_B), 2.82 – 2.70 (m, 4H, C*H* iPr), 1.64 – 1.57 (obs m, 2H, $NCH_2CH_AH_B$), 1.52 – 1.43 (obs m, 2H, $NCH_2CH_AH_B$), 1.21 (d, *J* = 6.8 Hz, 6H, $CH(CH_3)_2$ iPr), 1.19 (d, *J* = 6.8 Hz, 6H, $CH(CH_3)_2$ iPr), 1.18 (d, *J* = 6.8 Hz, 6H, $CH(CH_3)_2$ iPr), 1.17 (d, *J* = 6.8 Hz, 6H, $CH(CH_3)_2$ iPr), 1.24 – 1.08 (m, 4H, OCH_2CH_2).

^{13}C NMR (126 MHz, $CDCl_3$) δ 163.6, 163.2, 153.9, 147.3, 145.9, 145.5, 134.9, 134.5, 133.8, 132.7, 130.6, 130.0, 129.1, 128.7, 127.6, 126.7, 125.6, 124.4, 124.3, 124.3, 123.3, 122.9, 122.4, 120.5, 115.6, 69.4, 50.6, 29.9, 29.5, 29.4, 26.3, 24.3, 24.2, 24.2, 24.1.

MS (ESI): m/z calc. for $C_{80}H_{71}N_8O_6$ $[M+H]^+$: 1239.5497; found 1239.5508

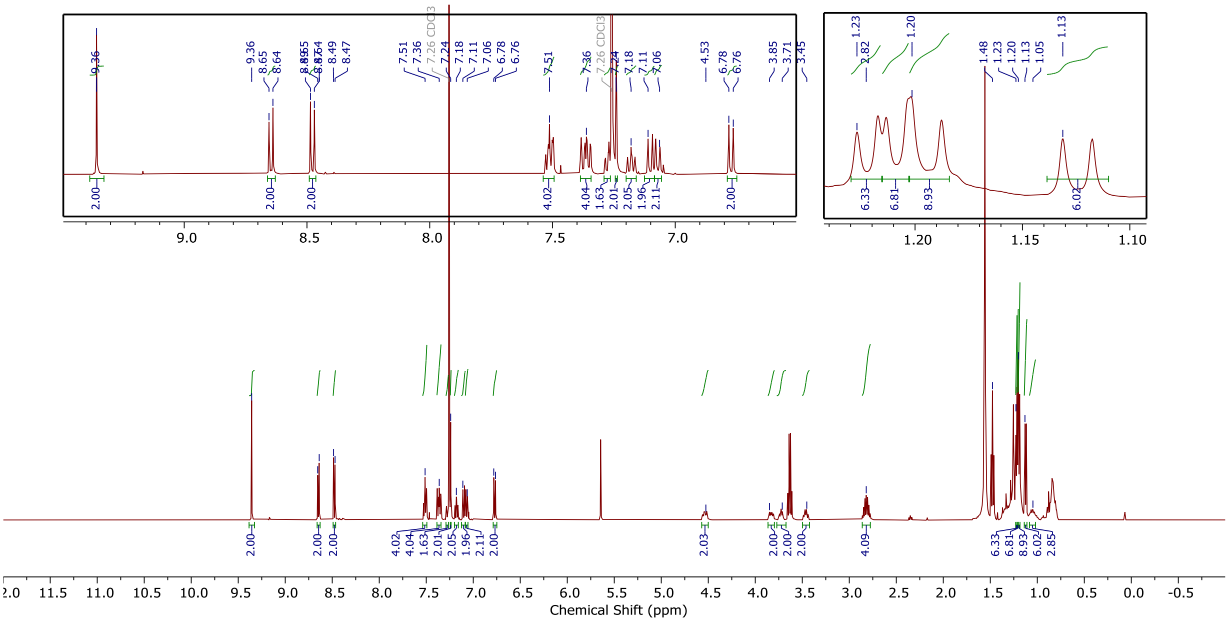

^1H NMR spectrum of ***PM*-2a** (500 MHz, $CDCl_3$).

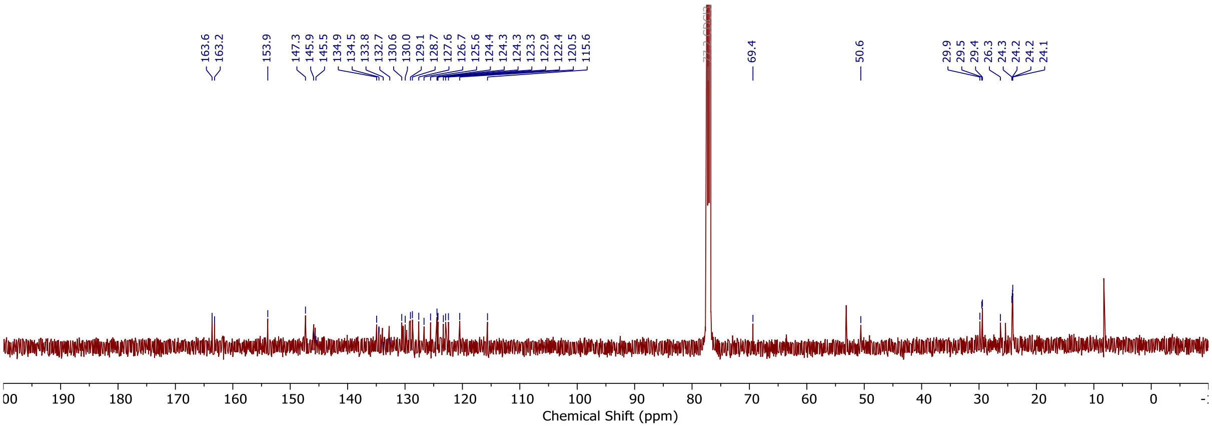

^{13}C NMR spectrum of ***PM*-2a** (126 MHz, $CDCl_3$).

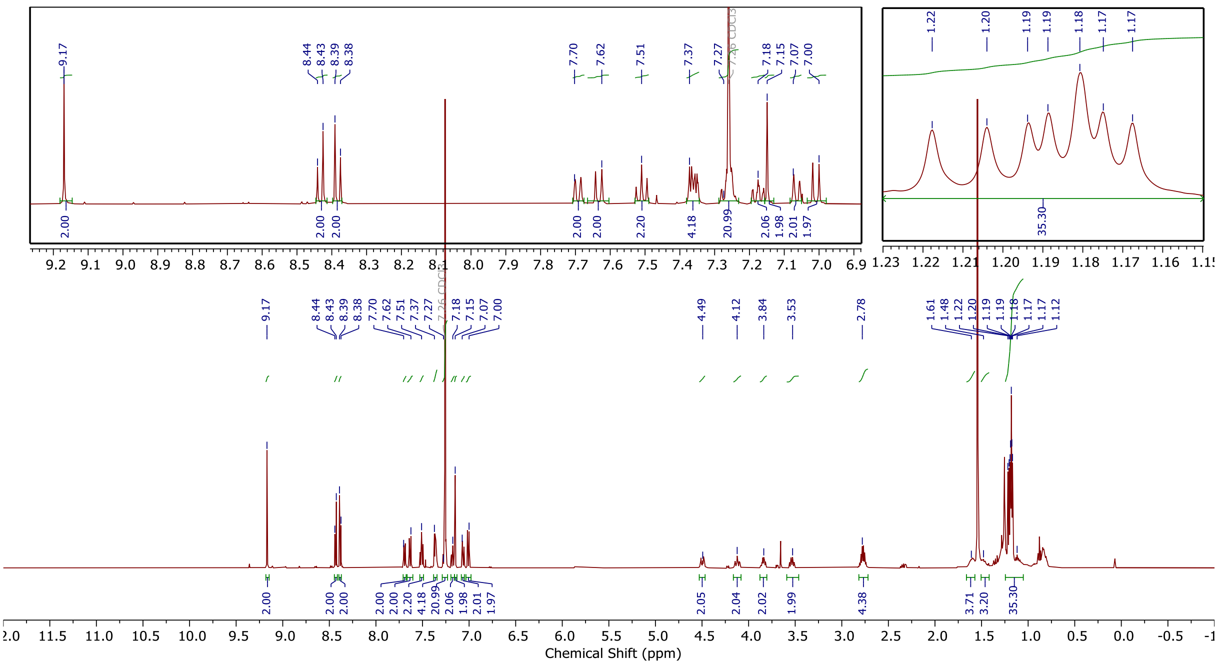

^{1}H NMR spectrum of ***PP*-2a** (500 MHz, $CDCl_3$).

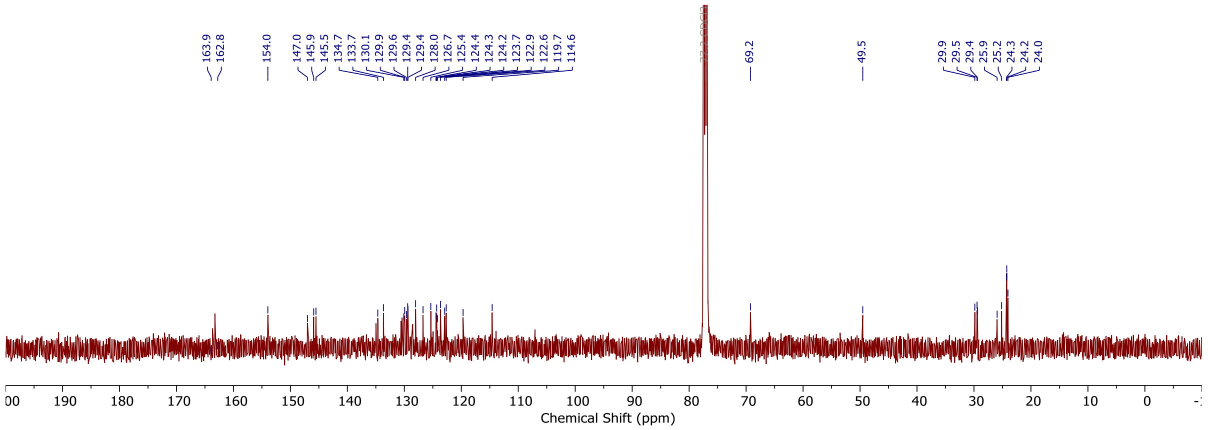

^{13}C NMR spectrum of ***PP*-2a** (126 MHz, $CDCl_3$).

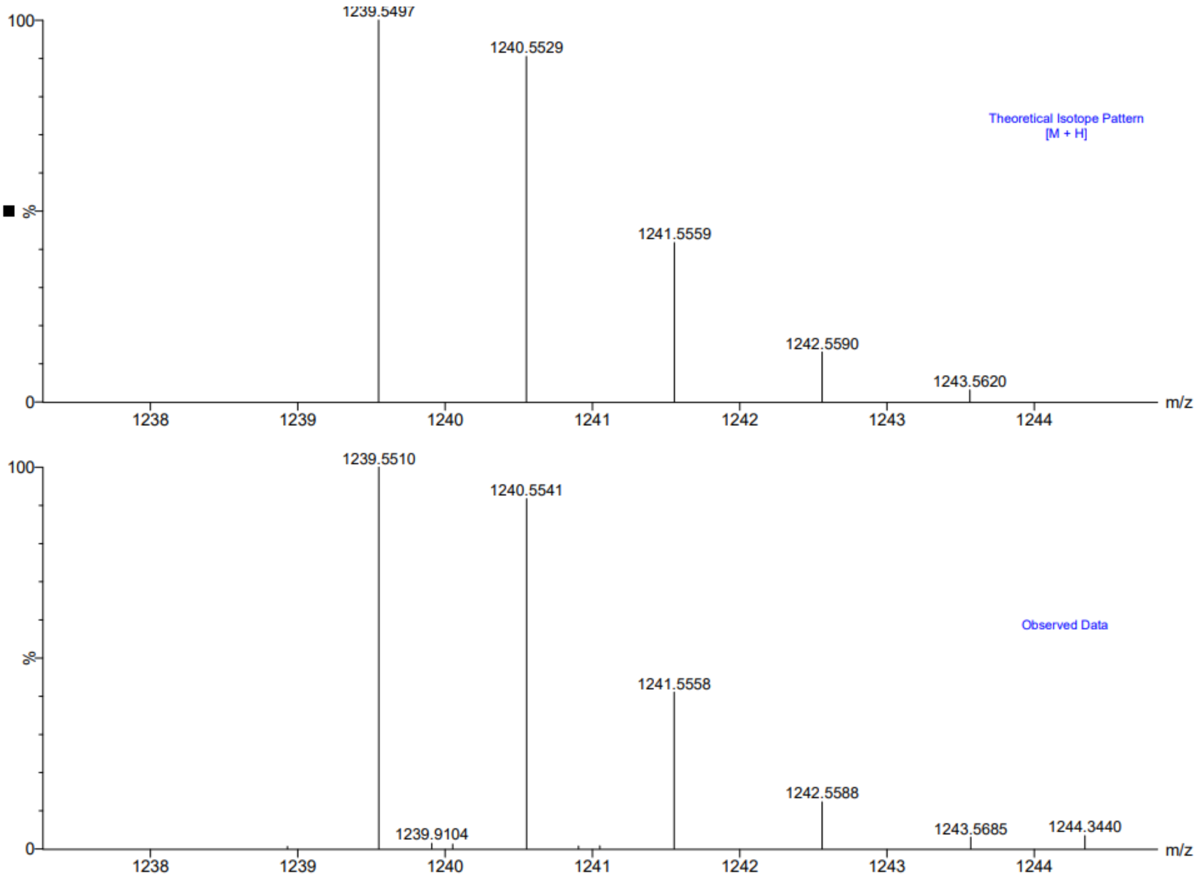

Calculated (top) and observed (bottom) ESI MS data for compound ***PM*-2a**.

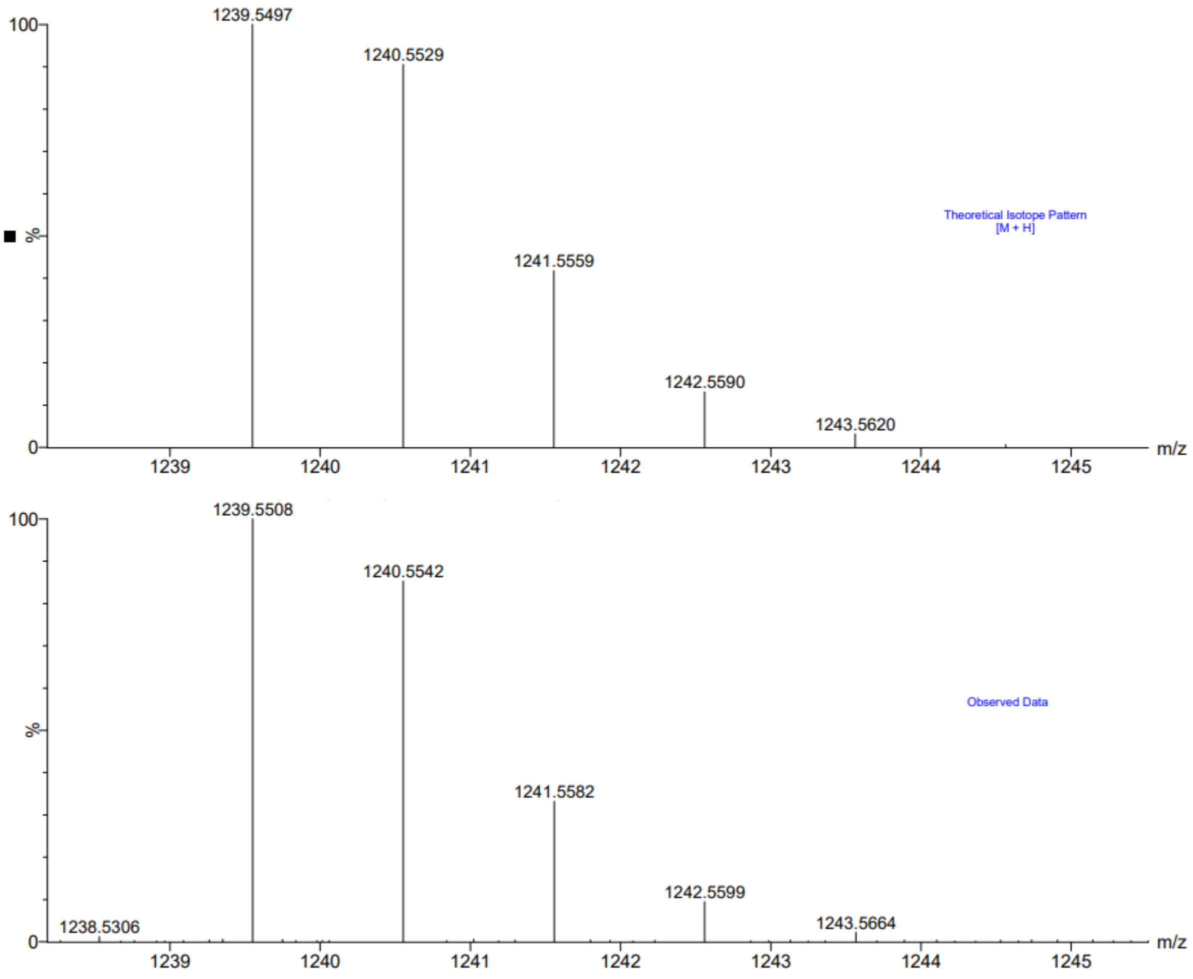

Calculated (top) and observed (bottom) ESI MS data for compound ***PP*-2a**.

P-BINOL hexyl PDI macrocycle 2b

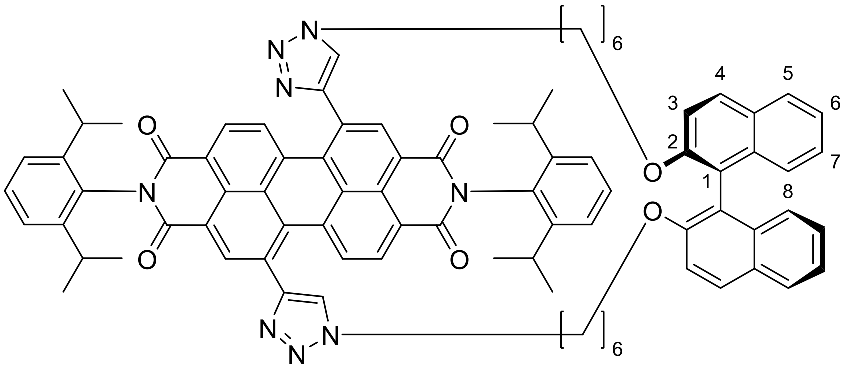

The general procedure above was followed with dialkynyl PDI **8** (113 mg, 0.149 mmol, 1 eq), ***P***-2,2'-bis((6-azidohexyl)oxy)-1,1'-binaphthalene ***P*-12b** (80.0 mg, 0.149 mmol, 1 eq), CH_2Cl_2 (680 mL), TBTA (31 mg, 59.6 µmol, 0.4 eq) and $Cu(MeCN)_4{\cdot}PF_6$ (22 mg, 59.6 µmol, 0.4 eq). The reaction was completed after four days and purified by flash column chromatography (SiO_2, 0.5:100 MeOH:CH_2Cl_2) to give two diastereomers of macrocycle ***PM*-2b** and ***PP*-2b** as purple solids. Diastereomer 1 ***PM*-2b** did not require any further purification (54.0 mg, 42%), whilst diastereomer 2 ***PP*-2b** was further purified by preparative TLC (SiO_2, 1:100 MeOH:CH_2Cl_2) (isolated 4.3 mg, 3.3%, calculated yield from crude NMR 11%). The ratio of diastereomers 1 and 2 are calculated to be 4:1 by ^{1}H NMR spectroscopy of the crude product (see Section 3.4).

The reaction was repeated with its enantiomer ***M*-12b** to yield ***MP*-2b** and ***MM*-2b** with near identical results and spectroscopic data (excluding CD and chiral HPLC).

Diastereomer 1 ***PM*-2b**:

^{1}H NMR (400 MHz, $CDCl_3$) δ 9.14 (s, 2H, Ar*H* Ortho PDI), 8.55 (d, *J* = 8.1 Hz, 2H, Ar*H* Ortho PDI), 8.47 (d, *J* = 8.1 Hz, 2H, Ar*H* Bay PDI), 7.49 (t, *J* = 7.8 Hz, 2H, iPr_2-Ar*H*), 7.41 – 7.34 (m, 4H, iPr_2-Ar*H*, C(5)*H*), 7.29 (dd, *J* = 7.8, 1.4 Hz, 2H, iPr_2-Ar*H*), 7.26 – 7.22 (m, 2H, C(6)*H*), 7.21 (s, 2H, C*H* triazole), 7.15 (ddd, *J* = 8.3, 6.7, 1.4 Hz, 2H, C(7)*H*), 7.00 (dd, *J* = 8.3, 1.2 Hz, 2H, C(8)*H*), 6.95 (d, *J* = 9.0 Hz, 2H, C(4)*H*), 6.79 (d, *J* = 9.0 Hz, 2H, C(3)*H*), 4.53 (ddd, *J* = 13.6, 6.1, 4.1 Hz, 2H, $NC\mathit{H}_AH_B$), 3.78 (ddd, *J* = 13.6, 9.6, 3.5 Hz, 2H, $NCH_A\mathit{H}_B$), 3.74 – 3.68 (m, 2H, $OC\mathit{H}_AH_B$), 3.58 – 3.46 (m, 2H, $OCH_A\mathit{H}_B$), 2.79 (p, *J* = 6.8 Hz, 2H, C*H* iPr_A), 2.62 (p, *J* = 6.8 Hz, 2H, C*H* iPr_B), 1.99 – 1.71 (m, 2H, $NCH_2C\mathit{H}_AH_B$), 1.50 (obs dq, *J* = 6.6, 3.0 Hz, 2H, $NCH_2CH_A\mathit{H}_B$), 1.20 (app t, *J* = 6.8 Hz, 12H, $CH(C\mathit{H}_3)_2$ iPr_A), 1.02 (d, *J* = 6.8 Hz, 6H, $CH(C\mathit{H}_3)_2$ iPr_B), 0.96 (d, *J* = 6.8 Hz, 6H, $CH(C\mathit{H}_3)_2$ iPr_B) 1.30 – 0.57 (obs m, 12H, $C\mathit{H}_2$).

^{13}C NMR (101 MHz, $CDCl_3$) δ 163.5, 163.2, 154.0, 146.9, 145.9, 145.5, 135.9, 135.0, 134.0, 133.3, 130.6, 130.4, 130.2, 129.9, 129.6, 129.5, 129.1, 128.9, 128.3, 127.3, 126.3, 125.6, 124.4, 124.2, 124.0, 123.2, 123.1, 122.3, 120.4, 115.0, 69.1, 49.7, 29.8, 29.4, 28.8, 28.6, 24.6, 24.2, 24.2, 24.1, 24.0.

MS (ESI): m/z calc. for $C_{84}H_{79}N_8O_6$ $[M+H]^+$: 1295.6123; found 1295.6139

Diastereomer 2 ***PP*-2b**:

^{1}H NMR (500 MHz, $CDCl_3$) δ 8.97 (s, 2H, Ar*H* Ortho PDI), 8.30 (d, *J* = 8.0 Hz, 2H, Ar*H* Ortho PDI), 7.88 (d, *J* = 8.0 Hz, 2H, Ar*H* Bay PDI), 7.71 (d, *J* = 8.1 Hz, 2H, C(5)*H*), 7.61 (d, *J* = 9.1 Hz, 2H, C(4)*H*), 7.51 (t, *J* = 7.8 Hz, 2H, iPr_2-Ar*H*), 7.37 (dd, *J* = 8.0, 1.4 Hz, 2H, iPr_2-Ar*H*), 7.34 (dd, *J* = 7.9, 1.4 Hz, 2H, iPr_2-Ar*H*), 7.29 (td, *J* = 8.1, 6.5, 1.3 Hz, 2H, C(6)*H*), 7.14 (td, *J* = 8.4, 6.5, 1.3 Hz, 2H, C(7)*H*), 6.92 (d, *J* = 8.4 Hz, 2H, C(8)*H*), 6.66 (d, *J* = 9.1 Hz, 2H, C(3)*H*), 5.87 (s, 2H, C*H* triazole), 4.28 (dt, *J* = 13.6, 3.9 Hz, 2H, $NC\mathit{H}_AH_B$), 3.10 (ddd, *J* = 10.5, 7.0, 2.9 Hz, 2H, $O\mathit{H}_AH_B$), 2.92 (ddd, *J* = 13.6, 11.5, 2.8 Hz, 2H, $NC\mathit{H}_AH_B$), 2.75 (p, *J* = 6.9 Hz, 2H, C*H* iPr), 2.67 (p, *J* = 6.8 Hz, 2H, C*H* iPr), 2.51 (ddd, *J* = 10.5, 8.4, 2.7 Hz, 2H, $OH_A\mathit{H}_B$), 1.89 – 1.69 (m, 2H, $NCH_2\mathit{H}_AH_B$), 1.38 – 1.27 (obs m, 2H, $NCH_2H_A\mathit{H}_B$), 1.24 (d, *J* = 6.8 Hz, 6H, $CH(C\mathit{H}_3)_2$ iPr), 1.19 (d, *J* = 6.8 Hz, 6H, $CH(C\mathit{H}_3)_2$ iPr), 1.15 (d, *J* = 6.8 Hz, 12H, $CH(C\mathit{H}_3)_2$ iPr), 1.20 – 1.07 (obs m, 2H, $C\mathit{H}_2$) 1.03 – 0.78 (obs m, 2H, $C\mathit{H}_2$), 0.39 (dt, *J* = 12.5, 4.6 Hz, 2H, $C\mathit{H}_2$), 0.34 – 0.25 (m, 2H, $C\mathit{H}_2$), 0.22 – 0.11 (m, 4H, $C\mathit{H}_2$).

^{13}C NMR (126 MHz, $CDCl_3$) δ 163.3, 163.2, 153.1, 146.7, 146.1, 145.0, 135.4, 135.3, 134.8, 133.7, 130.6, 130.5, 130.0, 130.0, 129.8, 129.5, 129.2, 129.1, 128.6, 128.0, 126.8, 125.4, 124.6, 124.3, 124.2, 124.1, 122.4, 122.3, 119.6, 113.6, 65.4, 47.1, 29.9, 29.7, 29.4, 28.6, 27.7, 24.2, 24.1, 24.1, 24.0, 22.6, 21.0.

MS (ESI): m/z calc. for $C_{84}H_{79}N_8O_6$ $[M+H]^+$: 1295.6123; found 1295.6139.

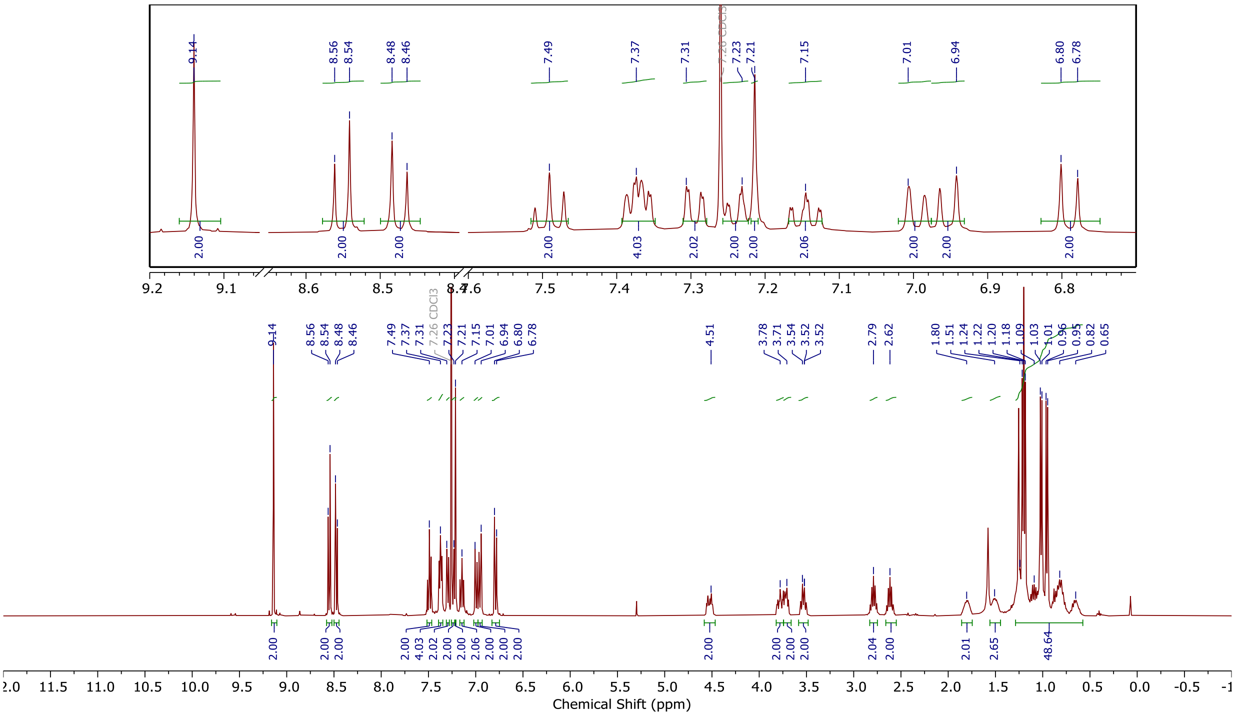

^{1}H NMR spectrum of ***PM*-2b** (400 MHz, $CDCl_3$).

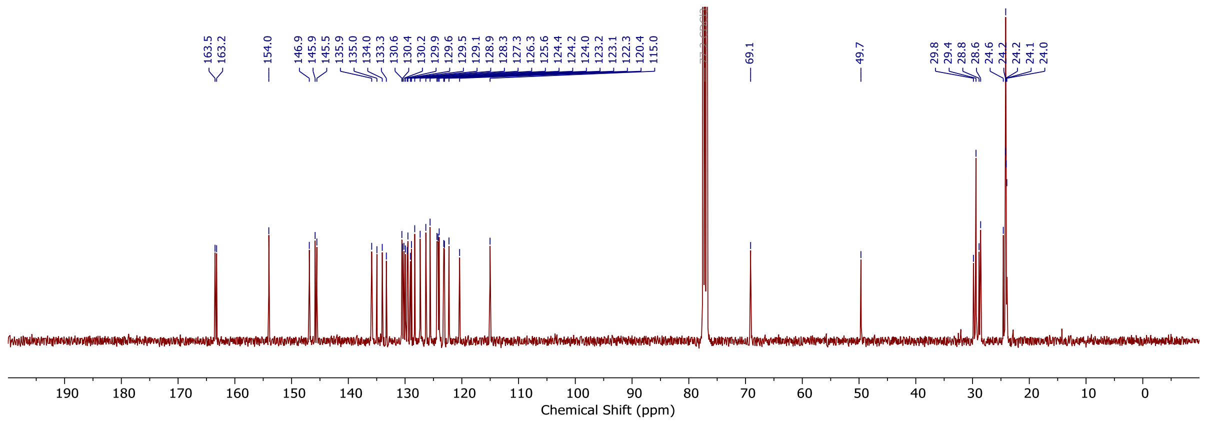

^{13}C NMR spectrum of ***PM*-2b** (101 MHz, $CDCl_3$).

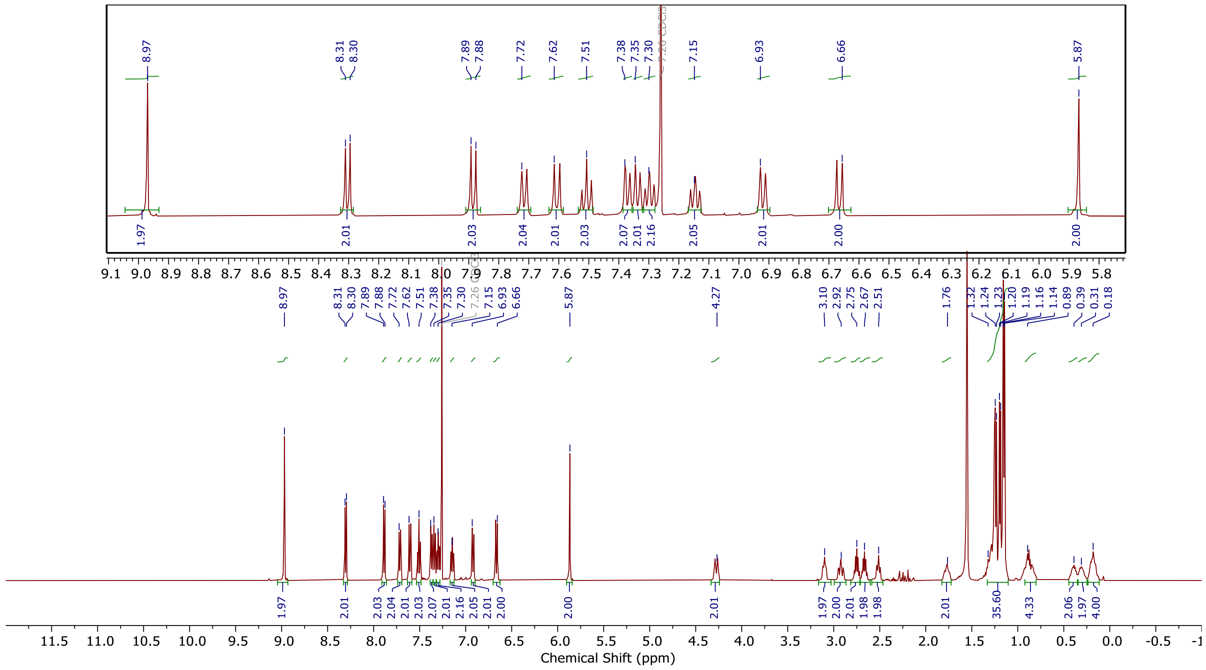

^{1}H NMR spectrum of ***PP*-2b** (500 MHz, $CDCl_3$).

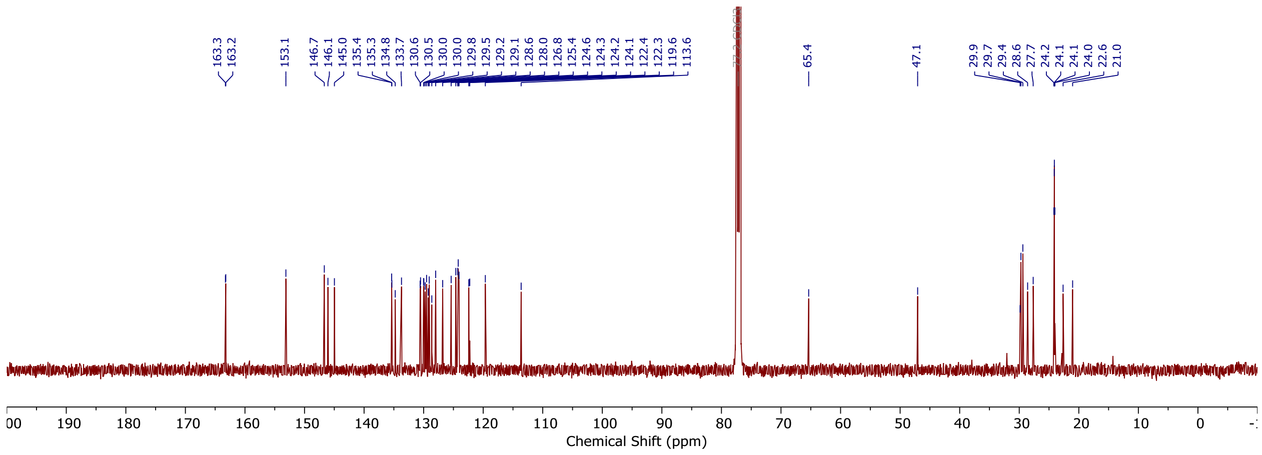

^{13}C NMR spectrum of ***PP*-2b** (126 MHz, $CDCl_3$).

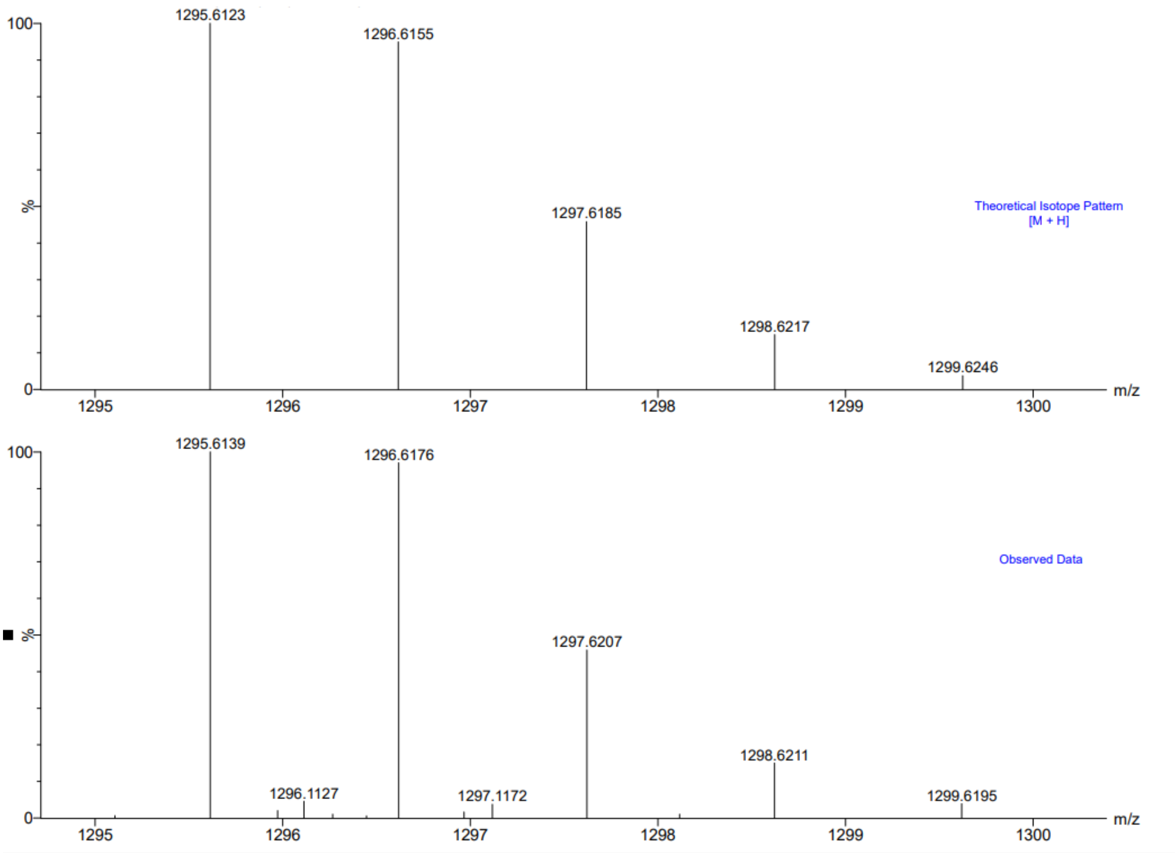

Calculated (top) and observed (bottom) ESI MS data for compound ***PM*-2b**.

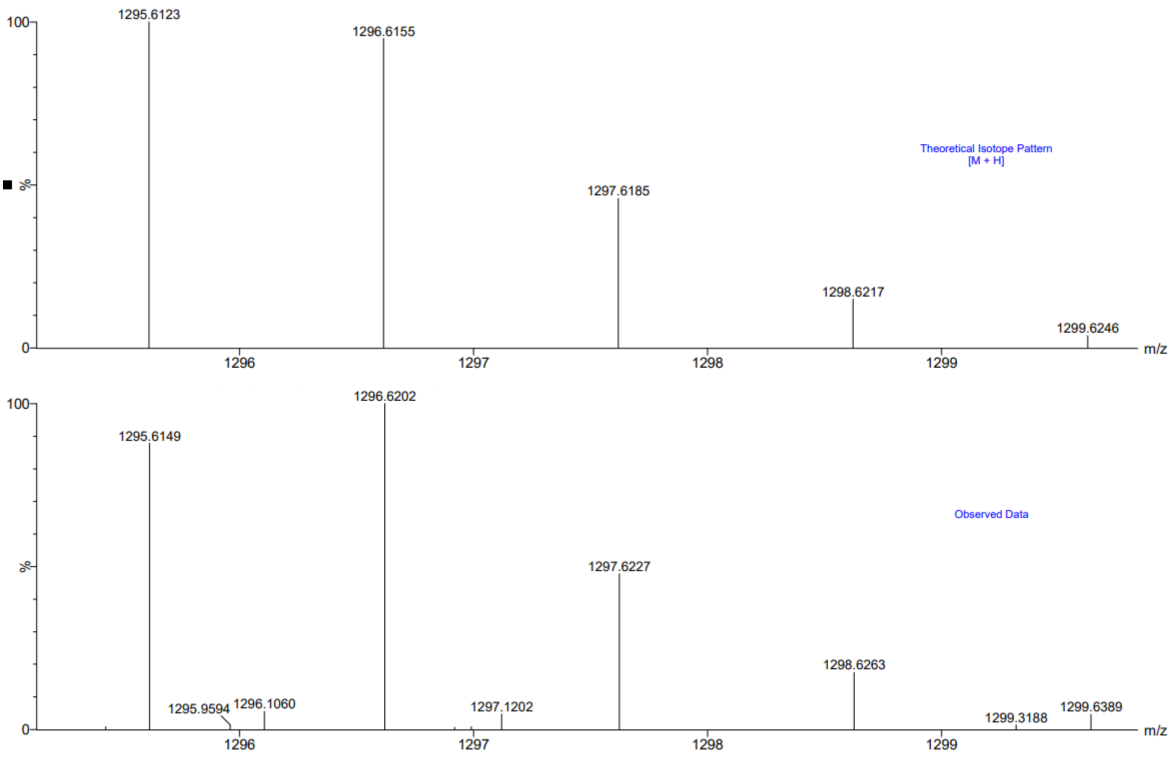

Calculated (top) and observed (bottom) ESI MS data for compound ***PP*-2b**.

***P*-BINOL octyl PDI macrocycle 2c**

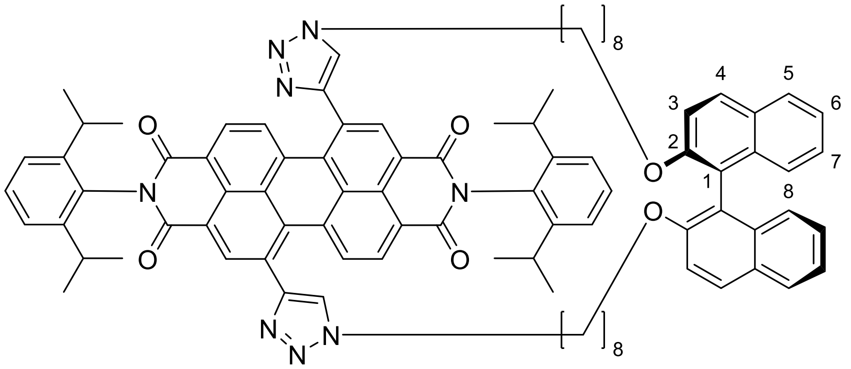

The general procedure above was followed with dialkynyl PDI **8** (150 mg, 198 µmol, 1 eq), ***P*-**2,2'-bis((8-azidooctyl)oxy)-1,1'-binaphthalene ***P*-12c** (117 mg, 198 µmol, 1 eq), CH_2Cl_2 (900 mL), TBTA (35 mg, 79 µmol, 0.4 eq) and $Cu(MeCN)_4{\cdot}PF_6$ (30 mg, 79 µmol, 0.4 eq). The reaction was completed after four days and purified by flash column chromatography (SiO_2, 0.5:100 MeOH:CH_2Cl_2) to give a mixture of two diastereomers of macrocycle ***PM*-2c** and ***PP*-2c** (80 mg, 33%). This mixture was separated by preparative TLC (SiO_2, 1:100 MeOH:CH_2Cl_2), where despite appearing as a single band on TLC, extracting the top 10% and bottom 10% respectively gives the diastereomer of macrocycle ***PM*-2c** (isolated 3.4 mg, 1.4%, calculated yield from mixture in NMR 17%) and ***PP*-2c** (isolated 2.7 mg, 1.1%, calculated yield from mixture in NMR 17%) as purple solids. The ratio of diastereomers is calculated to be 1:1 by ^{1}H NMR spectroscopy of the crude product (see Section 3.4).

Diastereomer 1 ***PM*-2c**:

^{1}H NMR (400 MHz, $CDCl_3$) δ 8.94 (s, 2H, Ar*H* Ortho PDI), 8.45 (d, *J* = 8.1 Hz, 2H, Ar*H* Ortho PDI), 8.26 (d, *J* = 8.1 Hz, 2H, Ar*H* Bay PDI), 7.63 (s, 2H, C*H* triazole), 7.52 (dd, *J* = 8.0, 1.1 Hz, 2H, C(5)*H*), 7.49 (t, *J* = 7.8 Hz, 2H, iPr$_2$-Ar*H*), 7.43 (d, *J* = 8.9 Hz, 2H, C(4)*H*), 7.35 (dd, *J* = 8.0, 1.4 Hz, 2H, iPr$_2$-ArH_AH$_B$), 7.31 (dd, *J* = 7.8, 1.4 Hz, 2H, iPr$_2$-ArH$_A$$H_B$), 7.20 (ddd, *J* = 8.1, 6.7, 1.2 Hz, 2H, C(6)*H*), 7.11 (ddd, *J* = 8.4, 6.7, 1.1 Hz, 2H, C(7)*H*), 7.01 (obs dd, *J* = 8.4 Hz, 1.2, 2H, C(8)*H*), 7.00 (d, *J* = 8.9 Hz, 2H, C(3)*H*), 4.54 (dt, *J* = 13.5, 5.5 Hz, 2H, NCH_AH$_B$), 4.39 (dt, *J* = 13.5, 6.5 Hz, 2H, NCH$_A$$H_B$), 3.71 – 3.61 (m, 2H, O$H_AH_B$), 3.55 (dt, *J* = 9.3, 6.4 Hz, 2H, OH$_A$$H_B$), 2.74 (h, *J* = 6.7 Hz, 2H, C*H* iPr$_A$), 2.60 (p, *J* = 6.7 Hz, 2H, C*H* iPr$_B$), 1.19 (d, *J* = 6.8 Hz, 6H, CH(CH_3)$_2$ iPr$_A$), 1.16 (d, *J* = 6.8 Hz, 6H, CH(CH_3)$_2$ iPr$_A$), 1.06 (d, *J* = 6.8 Hz, 6H, CH(CH_3)$_2$ iPr$_B$), 0.99 (d, *J* = 6.8 Hz, 6H, CH(CH_3)$_2$ iPr$_B$), 1.84 – 0.52 (obs m, 24H, CH_2).

^{13}C NMR (101 MHz, $CDCl_3$) δ 163.4, 163.3, 154.5, 147.4, 145.8, 145.7, 135.6, 135.2, 134.2, 133.8, 130.5, 130.5, 129.9, 129.9, 129.7, 129.6, 129.2, 129.0, 128.7, 127.5, 126.1, 125.7, 124.3, 124.2, 123.5, 123.0, 122.8, 122.2, 116.0, 50.6, 32.1, 29.8, 29.4, 29.2, 28.2, 27.7, 25.2, 25.0, 24.1, 24.0, 22.8.

MS (ESI): m/z calc. for $C_{88}H_{86}N_8O_6Na$ [M+Na]$^+$: 1373.6569; found 1373.6609

Diastereomer 2 ***PP*-2c**:

^{1}H NMR (400 MHz, $CDCl_3$) δ 9.02 (s, 2H, Ar*H* Ortho PDI), 8.34 (d, *J* = 8.0 Hz, 2H, Ar*H* Ortho PDI), 8.19 (d, *J* = 8.0 Hz, 2H, Ar*H* Bay PDI), 7.73 (br d, *J* = 8.8 Hz, 4H, C(4)*H*, C(5)*H*), 7.50 (t, *J* = 8.2 Hz, 2H, iPr$_2$-Ar*H*), 7.41 (s, 2H, C*H* triazole), 7.38 – 7.31 (m, 4H, iPr$_2$-Ar*H*), 7.25 – 7.23 (obs ddd, *J* = 8.6, 6.7, 1.2 Hz, 2H, C(6)*H*), 7.14 (ddd, *J* = 8.3, 6.7, 1.3 Hz, 2H, C(7)*H*), 7.10 (d, *J* = 9.1 Hz, 2H, C(3)*H*), 7.03 (dd, *J* = 8.3, 1.2 Hz, 2H, C(8)*H*), 4.45 (dt, *J* = 13.3, 6.4 Hz, 2H, NCH_AH$_B$), 4.36 (dt, *J* = 13.3, 6.1 Hz, 2H, NCH$_A$$H_B$), 3.56 (dt, *J* = 9.5, 6.5 Hz, 2H, OCH_AH$_B$),

3.50 (dt, J = 9.5, 6.5 Hz, 2H, OCH$_A$$H_B$), 2.81 – 2.66 (m, 4H, C$H$ iPr$_A$), 1.86 – 1.70 (m, 4H, , OCH$_2$$H_2$), 1.18 (d, J = 6.9 Hz, 6H, CH(CH_3)$_2$ iPr), 1.18 (d, J = 7.1 Hz, 6H, CH(CH_3)$_2$ iPr), 1.16 (d, J = 7.1 Hz, 6H, CH(CH_3)$_2$ iPr), 1.14 (d, J = 6.9 Hz, 6H, CH(CH_3)$_2$ iPr), 1.68 – 0.52 (obs m, 20H, CH_2)

^{13}C NMR (101 MHz, CDCl$_3$) δ 163.2, 163.1, 154.1, 147.2, 145.6, 145.5, 135.4, 135.0, 134.0, 133.8, 130.3, 129.8, 129.7, 129.7, 129.5, 129.4, 129.3, 129.2, 128.9, 128.8, 127.6, 126.1, 125.5, 124.3, 124.0, 123.6, 122.7, 122.6, 121.9, 120.6, 119.8, 115.6, 50.1, 31.9, 29.2, 29.2, 29.1, 28.2, 27.4, 25.1, 24.8, 24.1, 24.0.

MS (ESI): m/z calc. for $C_{88}H_{86}N_8O_6Na$ [M+Na]$^+$: 1373.6569; found 1373.6609

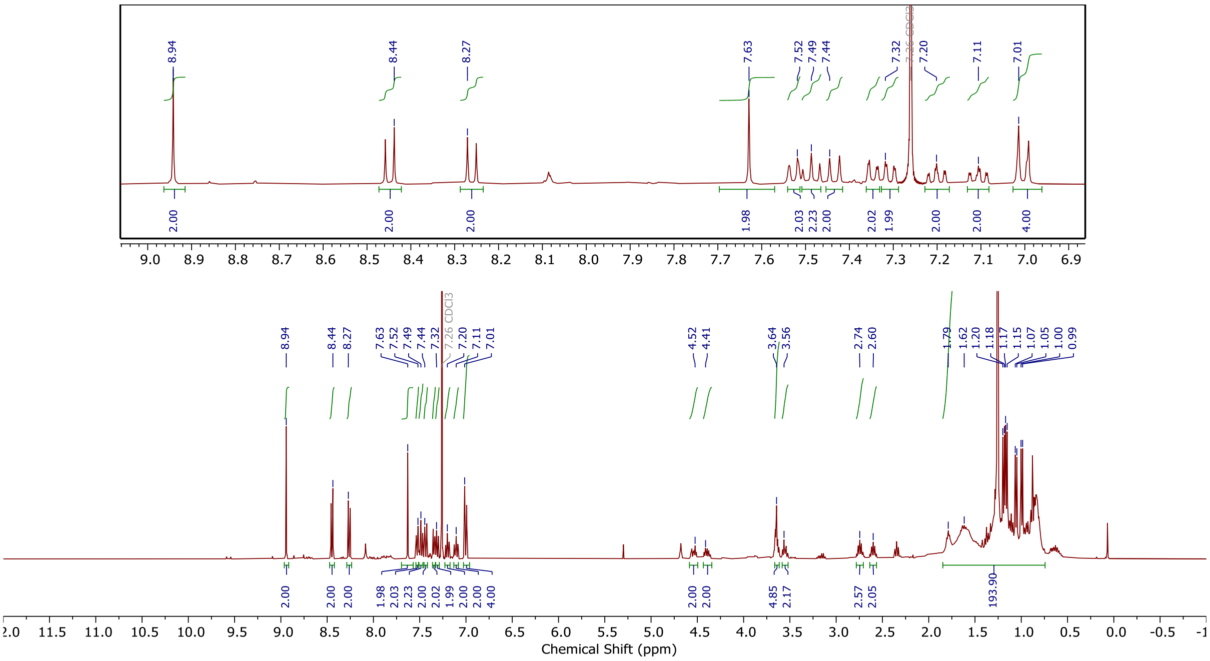

^{1}H NMR spectrum of ***PM*-2c** (400 MHz, CDCl$_3$).

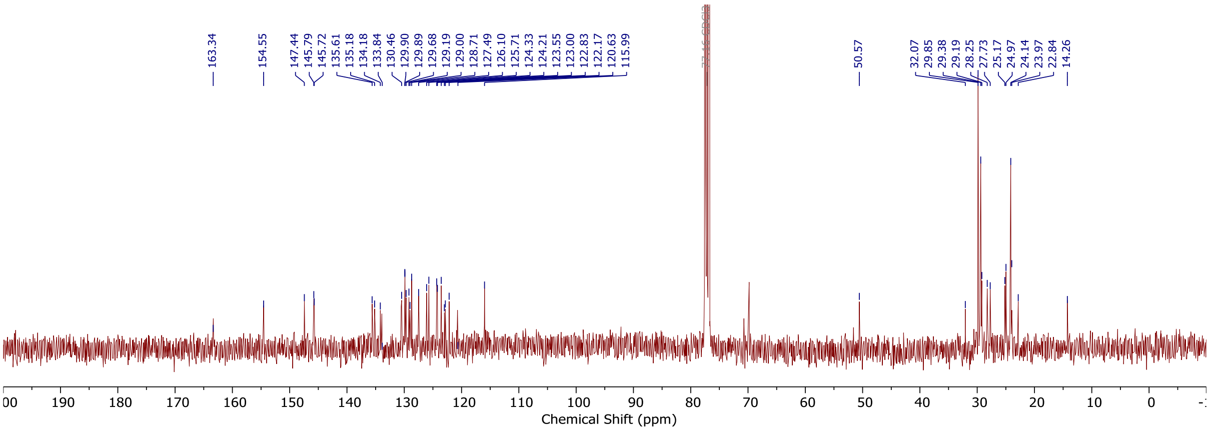

^{13}C NMR spectrum of ***PM*-2c** (101 MHz, CDCl$_3$).

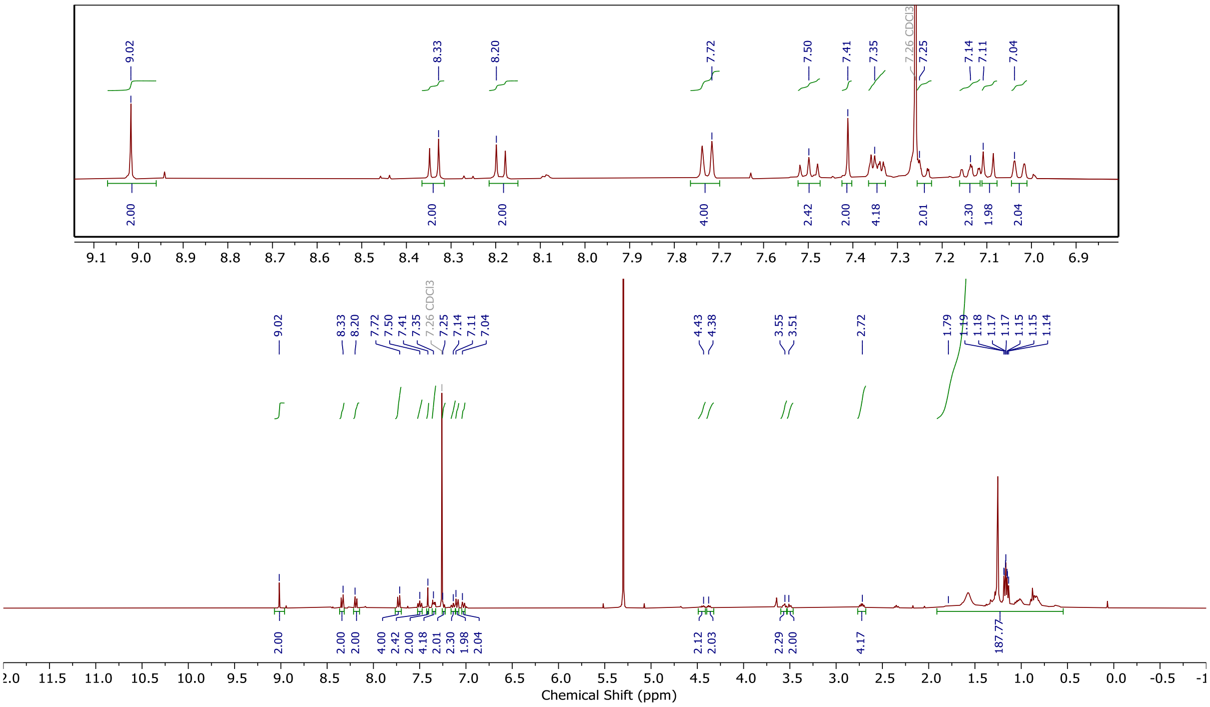

^{1}H NMR spectrum of ***PP*-2c** (400 MHz, $CDCl_3$).

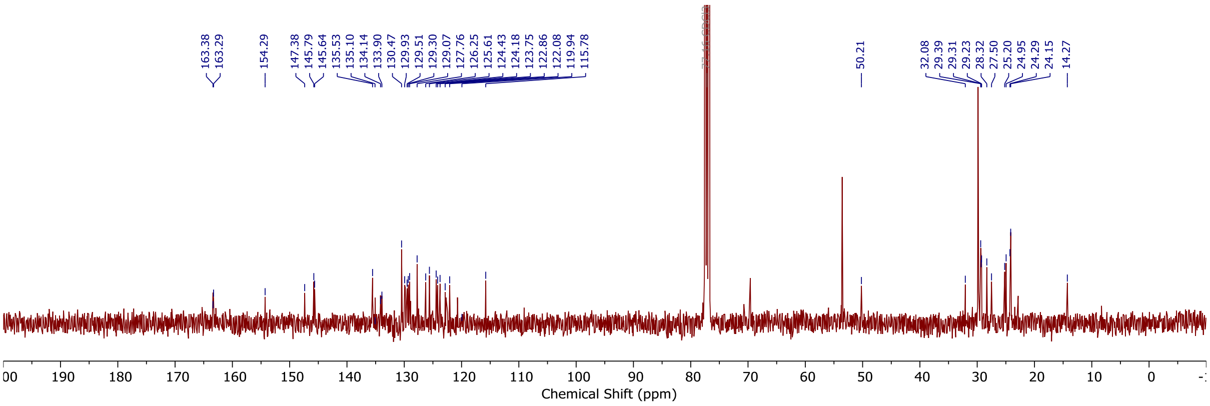

^{13}C NMR spectrum of ***PP*-2c** (101 MHz, $CDCl_3$).

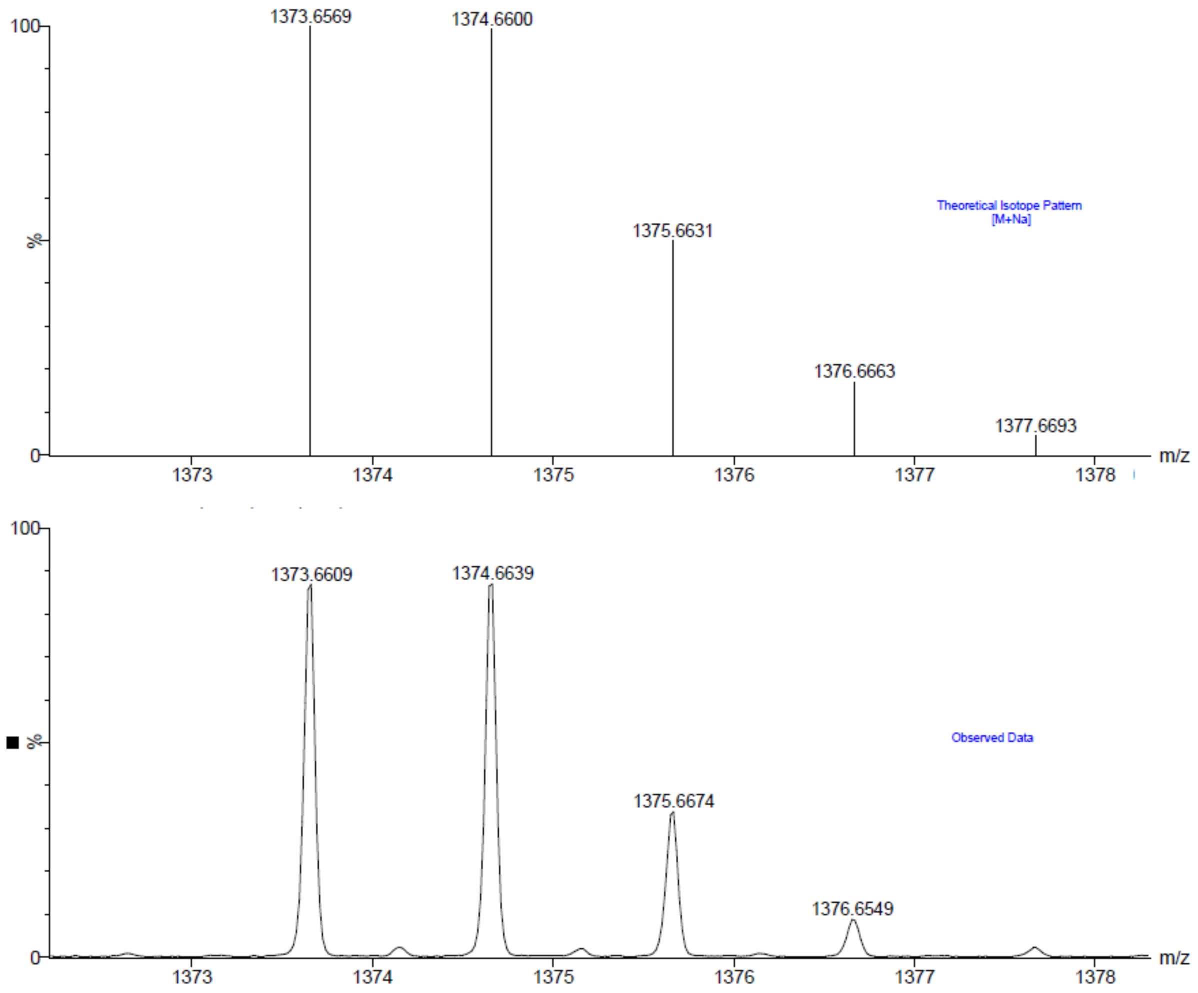

Calculated (top) and observed (bottom) ESI MS data for compound ***PM*-2c**.

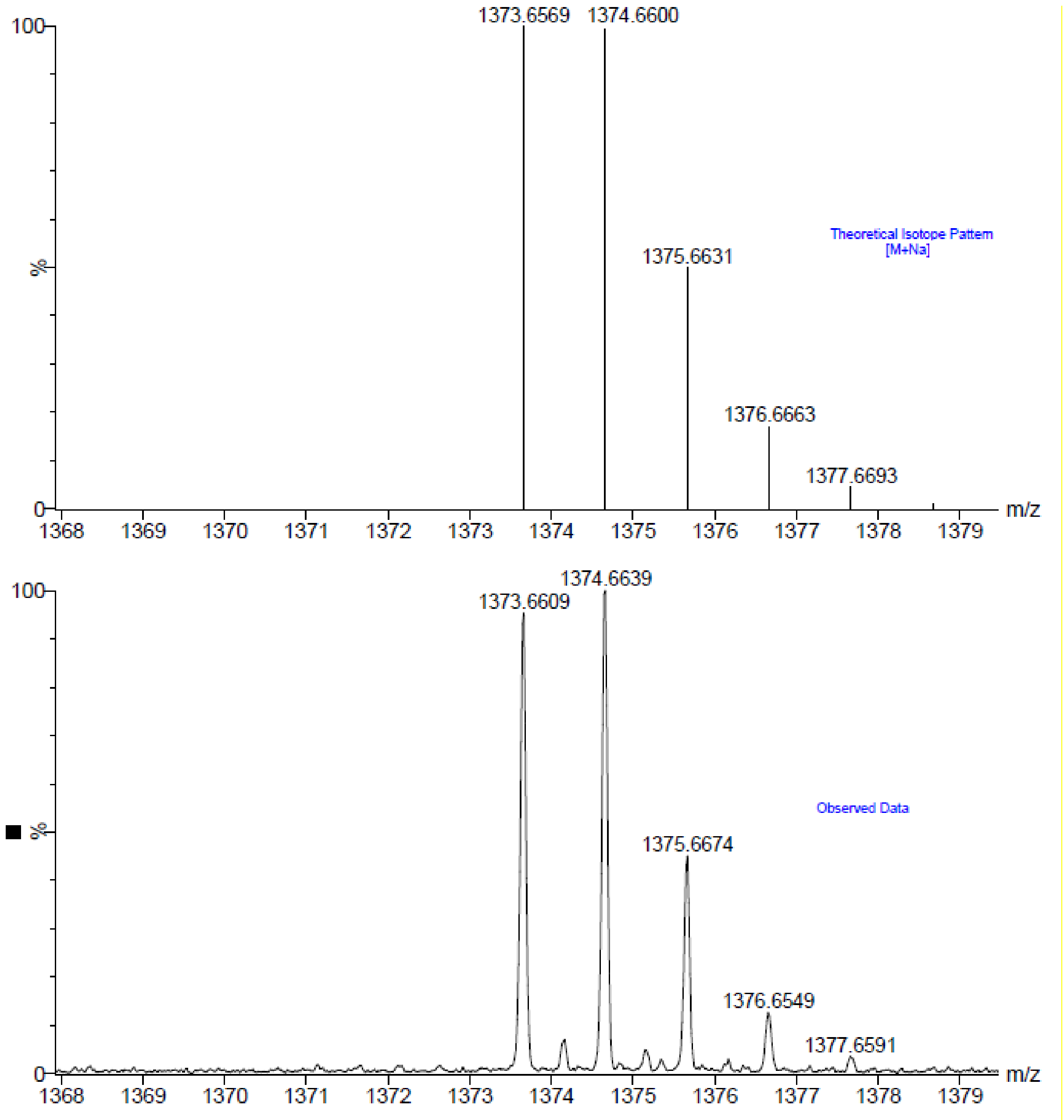

Calculated (top) and observed (bottom) ESI MS data for compound ***PP*-2c**.

1,4-Di(6-hexoxy)benzene PDI acyclic control 3

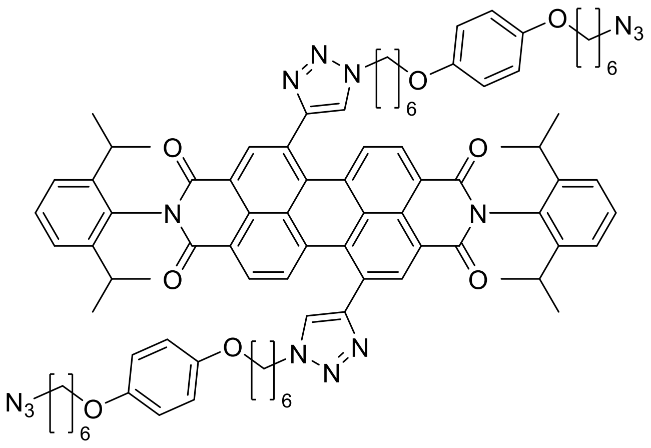

General Procedure B was followed with dialkynyl PDI **8** (50 mg, 0.0659 mmol, 1 eq), 1,4-di(6-azidohexoxy)benzene **11b** (237 mg, 0.659 mmol, eq), CH_2Cl_2 (15 mL), THPTA (11.5 mg, 26.4 µmol, 0.4 eq) and $Cu(MeCN)_4{\cdot}PF_6$ (9.82 mg, 26.4 µmol, 0.4 eq). The reaction was completed after three days and purified by flash column chromatography (SiO_2, 0.4:100 MeOH:CH_2Cl_2) to give PDI **3** as a purple solid (66 mg, 68%). The 1,7 and 1,6 regioisomers were separated by HPLC (Buckyprep, 2:60:38 IPA:n-hexane:CH_2Cl_2).

^{1}H NMR (400 MHz, $CDCl_3$) δ 8.75 (s, 2H, Ar*H* Ortho PDI), 8.34 (d, *J* = 8.1 Hz, 2H, Ar*H* PDI), 8.00 (d, *J* = 8.1 Hz, 2H, Ar*H* PDI), 7.88 (s, 2H, C*H* triazole), 7.50 (t, *J* = 7.9 Hz, 2H, iPr$_2$-Ar*H*), 7.35 (d, *J* = 7.9 Hz, 4H, iPr$_2$-Ar*H*), 6.83 – 6.73 (m, 8H, Ar*H* oxybenzene), 4.54 (t, *J* = 7.1 Hz, 4H, C*H*$_2$), 3.91 (t, *J* = 6.2 Hz, 4H, C*H*$_2$), 3.84 (t, *J* = 6.4 Hz, 4H, C*H*$_2$), 3.27 (t, *J* = 6.9 Hz, 4H, C*H*$_2$), 2.74 (p, *J* = 6.8 Hz, 4H, C*H* iPr), 2.07 (p, *J* = 7.3 Hz, 4H, C*H*$_2$), 1.85 – 1.69 (m, 8H, C*H*$_2$), 1.66 – 1.25 (m, 20H, C*H*$_2$), 1.17 (d, *J* = 6.8 Hz, 24H, CH(C*H*$_3$)$_2$ iPr).

^{13}C NMR (126 MHz, $CDCl_3$) δ 163.5, 163.5, 153.3, 153.2, 147.9, 145.8, 135.7, 135.3, 134.1, 130.6, 130.5, 129.9, 129.6, 129.5, 129.5, 129.1, 124.3, 122.8, 122.6, 122.1, 115.5, 115.5, 68.5, 68.3, 51.5, 50.9, 30.5, 29.9, 29.4, 29.4, 29.3, 28.9, 26.7, 26.4, 25.8, 25.7, 24.2, 24.1.

MS (ESI): m/z calc. for $C_{88}H_{99}N14O_8$ $[M+H]^+$: 1479.777; found 1479.7783

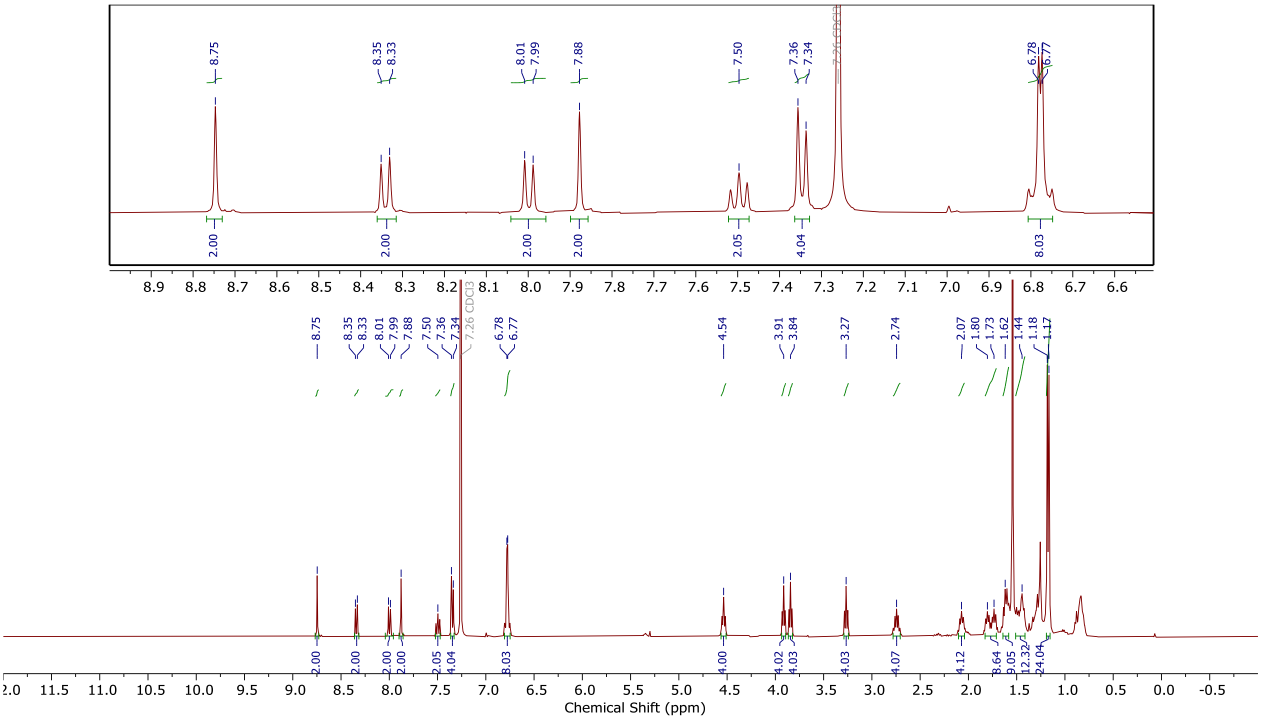

^{1}H NMR spectrum of **3** (400 MHz, $CDCl_3$).

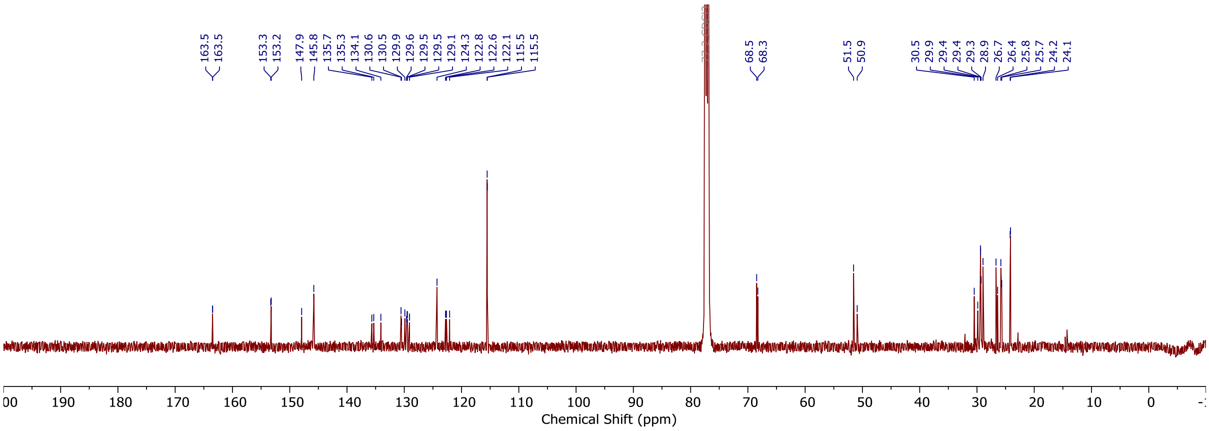

^{13}C NMR spectrum of **3** (126 MHz, $CDCl_3$).

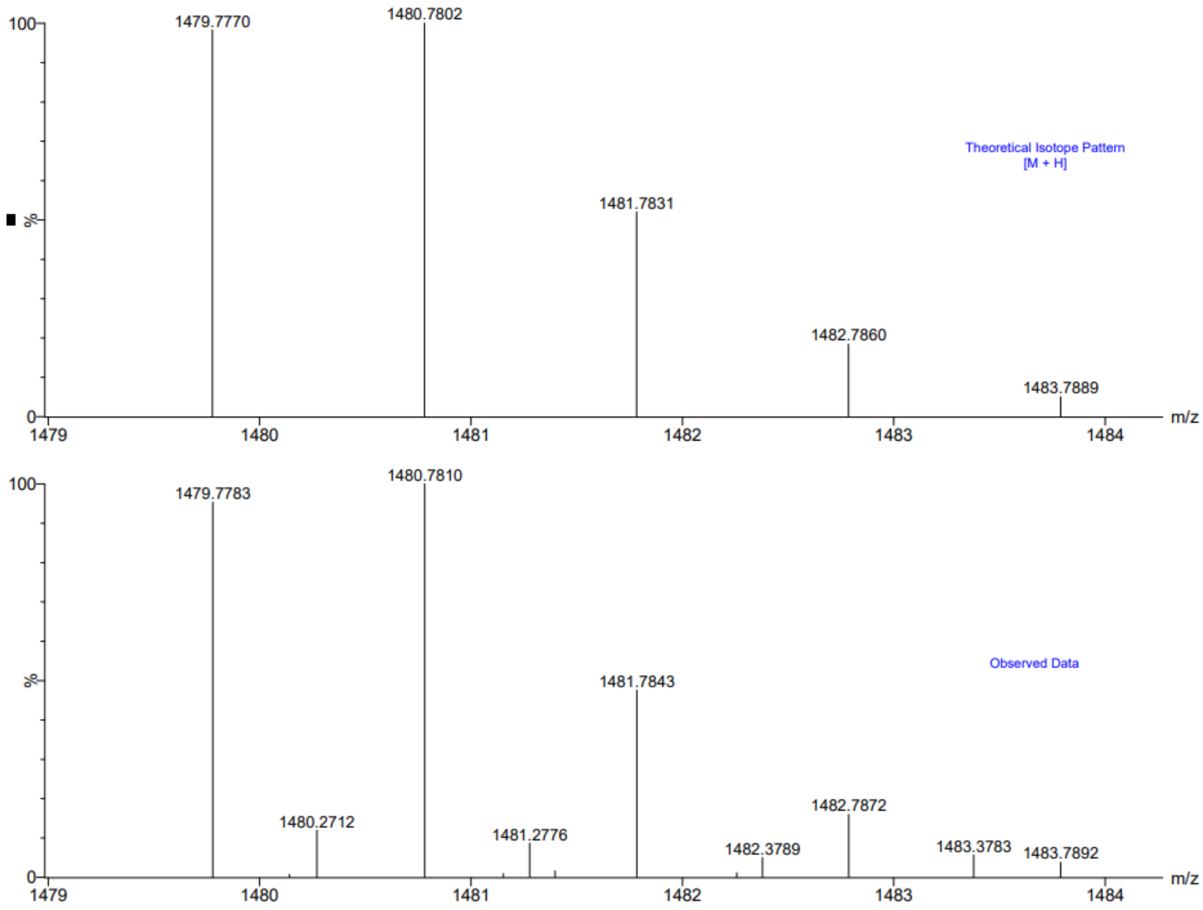

Calculated (top) and observed (bottom) ESI MS data for compound **3**.

P-BINOL hexyl PDI acyclic control 4

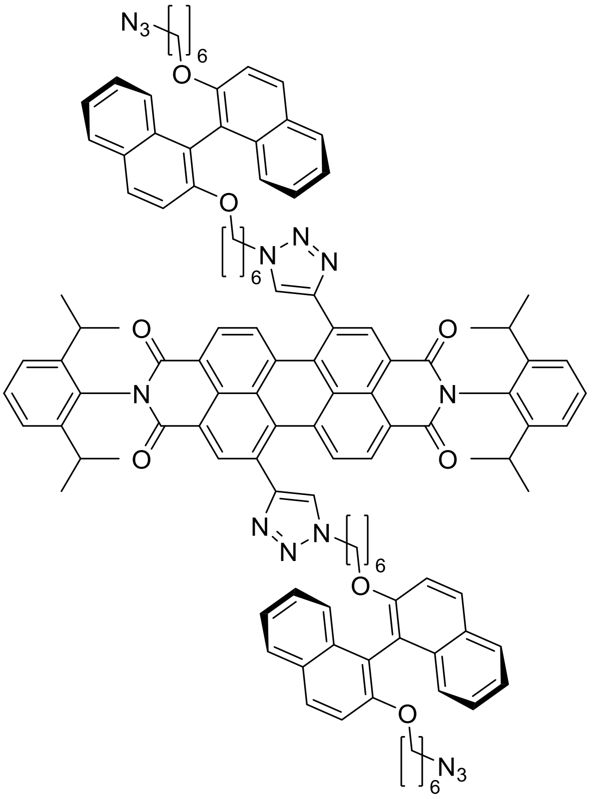

General Procedure B was followed with dialkynyl PDI **8** (53 mg, 69.8 µmol, 1 eq), (*M*)-2,2'-bis((6-azidohexyl)oxy)-1,1'-binaphthalene **12b** (375 mg, 0.698 mmol, 10 eq), CH_2Cl_2 (20 mL), THPTA (11.7 mg, 26.5 µmol, 0.4 eq) and Cu(MeCN)$_4$-PF$_6$ (8.30 mg, 26.5 µmol, 0.4 eq). Reaction was completed after 1 day and purified by flash column chromatography (SiO_2, 0.4:100 → 0.6:100 MeOH:CH_2Cl_2) to give PDI **4** as a purple solid (34 mg, 27%). The 1,7 and 1,6 regioisomers were separated by HPLC (Buckyprep, 2:60:38 IPA:n-hexane:CH_2Cl_2).

^{1}H NMR (400 MHz, $CDCl_3$) δ 8.75 (s, 2H, Ar*H* PDI), 8.35 (d, *J* = 8.1 Hz, 2H), 7.98 (d, *J* = 8.1 Hz, 2H), 7.93 – 7.87 (m, 12H), 7.84 (d, *J* = 8.1 Hz, 4H), 7.64 (s, 2H), 7.54 – 7.47 (m, 4H), 7.43 – 7.38 (m, 10H), 7.35 (d, *J* = 7.7 Hz, 6H), 7.30 (ddd, *J* = 8.1, 6.7, 1.3 Hz, 6H), 7.25 – 7.07 (m, 24H), 4.22 – 4.07 (m, 8H), 4.07 – 3.80 (m, 18H), 2.97 (t, *J* = 7.1 Hz, 9H), 2.75 (ddt, *J* = 19.8, 13.1, 6.8 Hz, 9H), 1.67 – 1.58 (m, 8H), 1.50 – 1.28 (m, 14H), 1.28 – 1.09 (m, 65H), 1.07 – 0.88 (m, 15H), 0.87 – 0.74 (m, 11H).

^{13}C NMR (101 MHz, $CDCl_3$) δ 163.5, 163.4, 154.7, 147.9, 145.8, 135.7, 135.4, 134.9, 134.4, 134.3, 130.5, 130.3, 129.9, 129.5, 129.4, 129.3, 129.2, 128.0, 126.3, 125.7, 124.3, 123.7, 122.8, 122.6, 122.1, 121.1, 120.7, 116.1, 115.9, 69.7, 69.5, 51.2, 50.4, 30.0, 29.5, 29.3, 29.1, 28.6, 26.1, 25.7, 25.2, 24.9, 24.1.

MS (ESI): m/z calc. for $C_{116}H_{115}N_{14}O_8$ $[M+H]^+$: 1831.9022; found 1831.8977

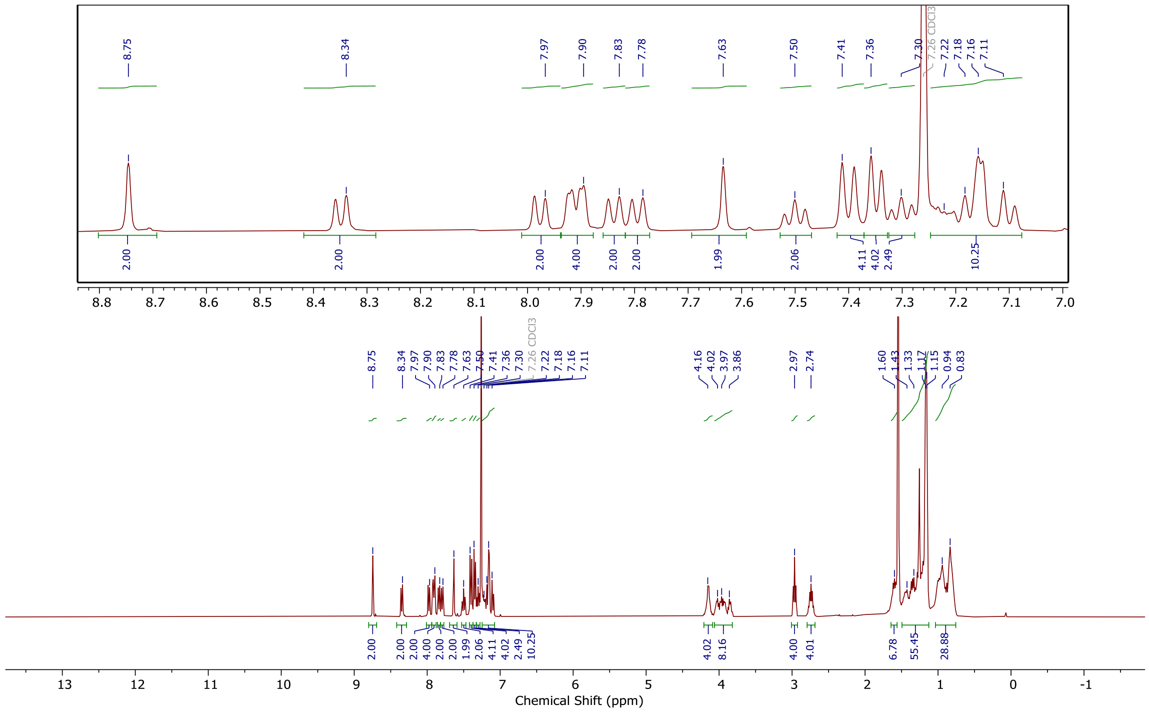

^{1}H NMR spectrum of **4** (400 MHz, $CDCl_3$).

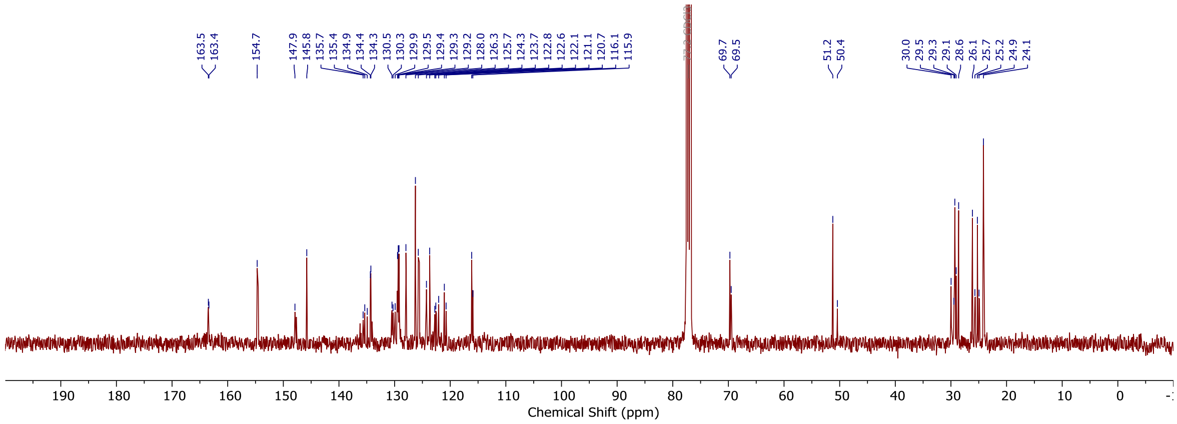

^{13}C NMR spectrum of **4** (101 MHz, $CDCl_3$).

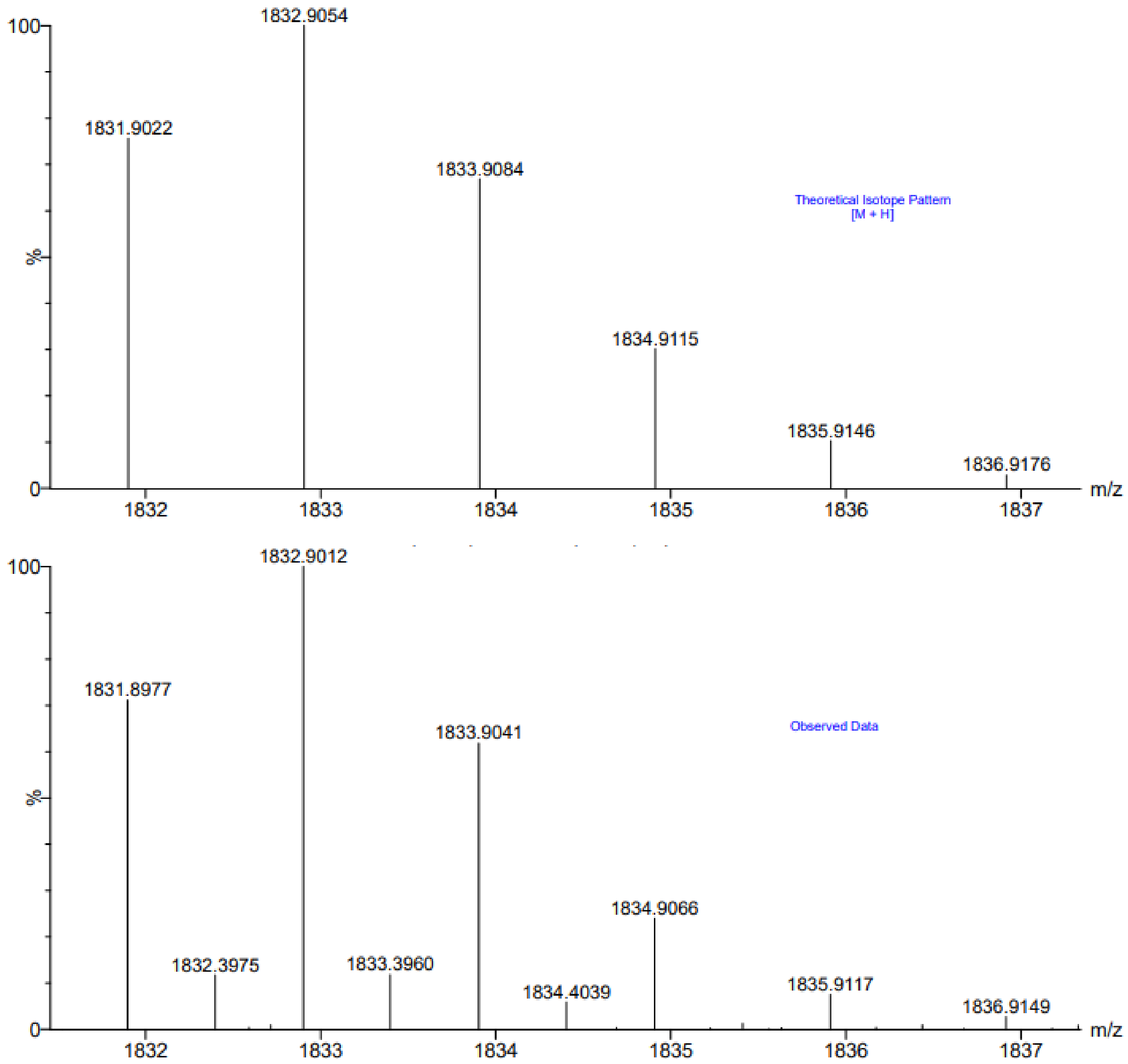

Calculated (top) and observed (bottom) ESI MS data for compound **4**.

Dihexyl triazole PDI acyclic control 5

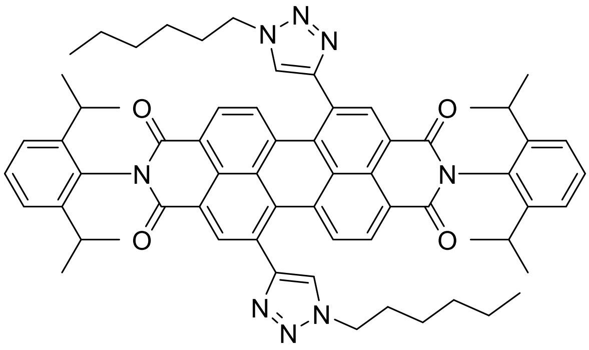

General Procedure B was followed with dialkynyl PDI **8** (50 mg, 65.9 µmol, 1 eq), 1-azidohexane (58.7 mg, 461 µmol, 7 eq), CH_2Cl_2 (260 mL), TBTA (9.82 mg, 26.4 µmol, 0.4 eq) and $Cu(MeCN)_4{\cdot}PF_6$ (14.0 mg, 26.4 µmol, 0.4 eq). The reaction was completed after two days and purified by flash column chromatography (SiO_2, 0.4:100 MeOH:CH_2Cl_2) to give PDI **4** as a purple solid (45 mg, 67%). The 1,7 and 1,6 regioisomers were separated by HPLC (Buckyprep, 2:38:60 IPA:n-hexane:CH_2Cl_2) as outlined above.

^{1}H NMR (400 MHz, $CDCl_3$) δ 8.76 (s, 2H, Ar*H* Ortho PDI), 8.34 (d, *J* = 8.1 Hz, 2H, Ar*H* Ortho PDI), 8.01 (d, *J* = 8.1 Hz, 2H, Ar*H* Bay PDI), 7.88 (s, 2H, C*H* triazole), 7.50 (t, *J* = 7.8 Hz, 2H, iPr_2-Ar*H*), 7.35 (d, *J* = 7.8 Hz, 4H, iPr_2-Ar*H*), 4.52 (t, *J* = 7.2 Hz, 4H, NCH_2), 2.74 (p, *J* = 6.8 Hz, 4H, C*H* iPr), 2.02 (p, *J* = 7.3 Hz, 4H, NCH_2CH_2), 1.47 – 1.31 (m, 12H, CH_2), 1.17 (d, *J* = 6.8 Hz, 24H, $CH(CH_3)_2$ iPr), 0.90 (t, *J* = 6.9 Hz, 6H, CH_2CH_3).

^{13}C NMR (101 MHz, $CDCl_3$) δ 163.3, 163.3, 145.7, 135.5, 135.2, 134.0, 130.4, 130.4, 129.7, 129.5, 129.4, 128.9, 124.1, 122.6, 122.5, 50.9, 31.1, 30.4, 29.2, 26.2, 24.0, 24.0, 22.4, 14.0.

MS (ESI): m/z calc. for $C_{64}H_{69}N_8O_4$ $[M+H]^+$: 1013.5442; found 1013.5493.

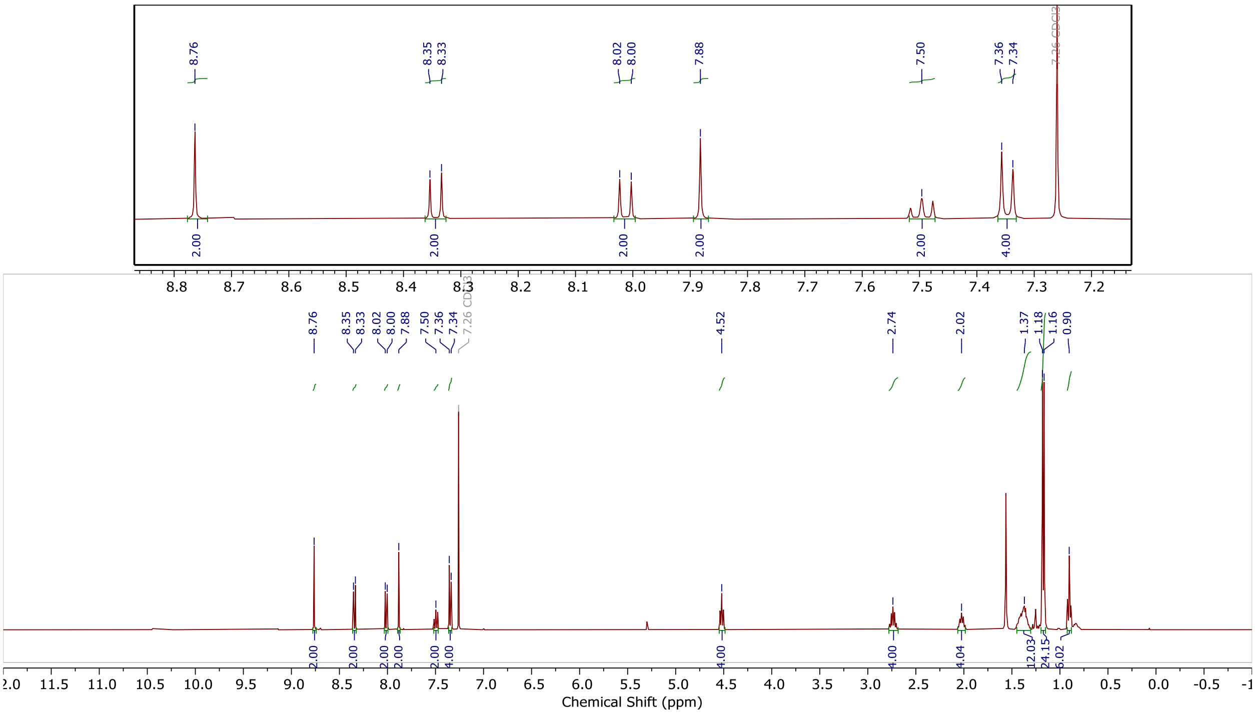

^{1}H NMR spectrum of **4** (400 MHz, $CDCl_3$).

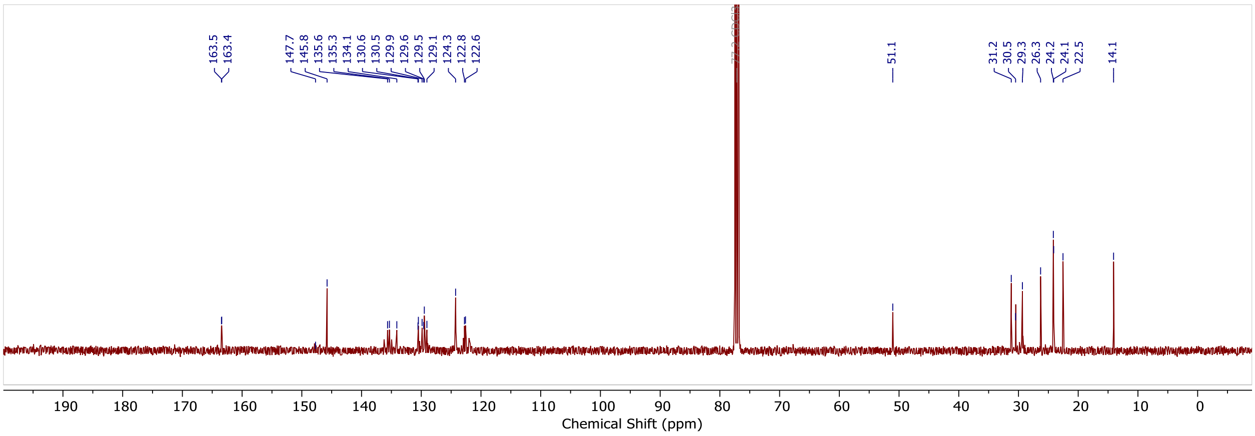

^{13}C NMR spectrum of **4** (101 MHz, $CDCl_3$).

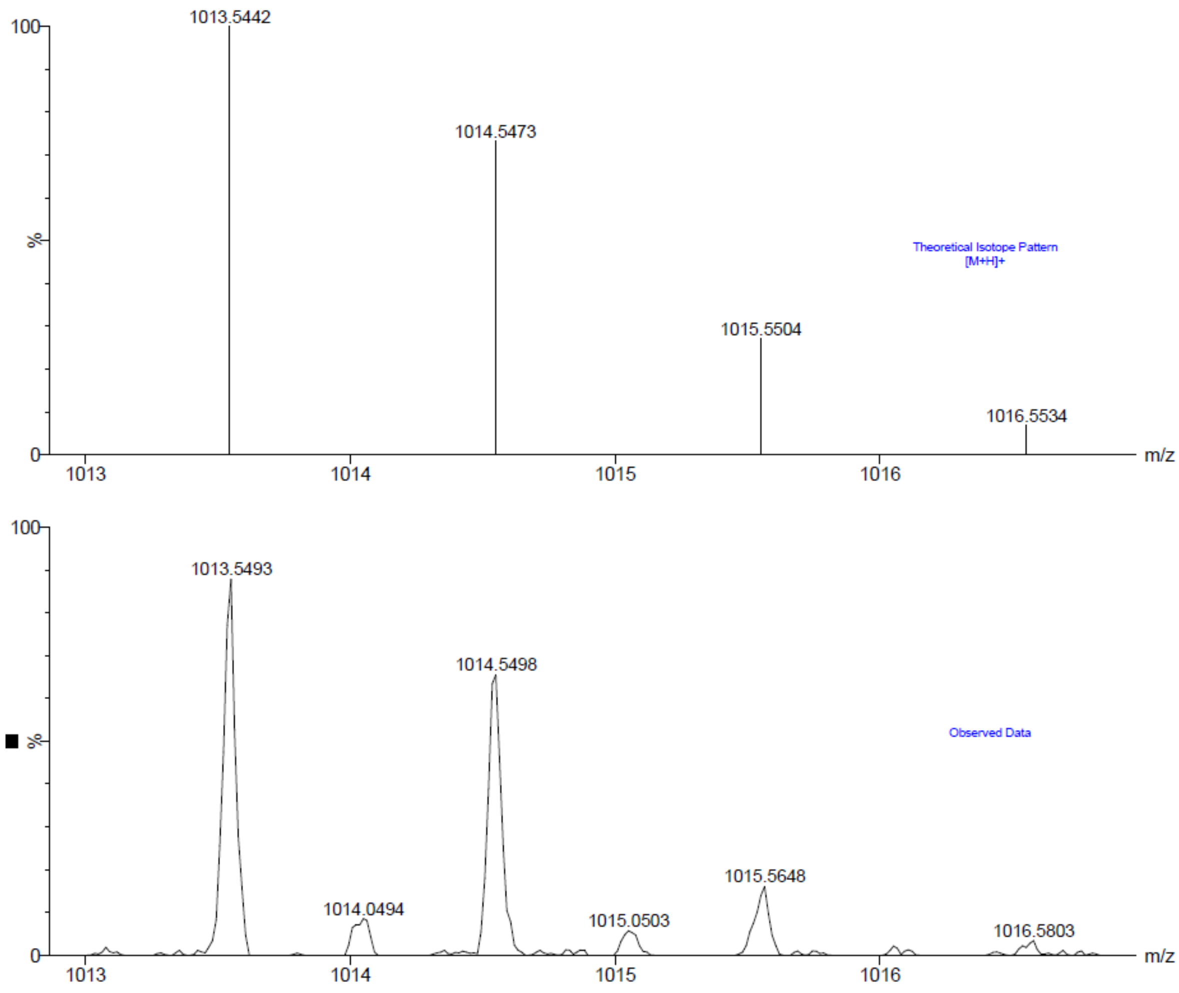

Calculated (top) and observed (bottom) ESI MS data for compound **5**.

1,7-Dibromo-perylene-3,4,9,10-tetracarboxylic dianhydride[5]

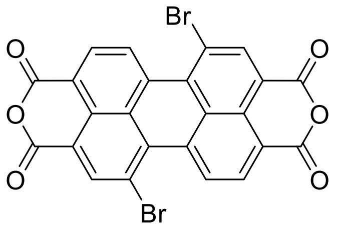

Perylene-3,4,9,10-tetracarboxylic dianhydride (20.0 g, 64.0 mmol, 1 eq) was dissolved in conc H_2SO_4 (300 mL) in a round bottomed flask. *N*-Bromosuccinimide (9.1 g, 79.8 mmol, 1.1 eq) was then added and the reaction flask was stoppered and left to stir at rt for 16 h. After which, additional *N*-bromosuccinimide (9.1 g, 79.8 mmol, 1.1 eq) was added and the flask was again stoppered and left to stir at 80 °C for 1 day. On cooling to rt, the resulting mixture was added onto a large beaker of ice, causing the formation of a red precipitate. Any remaining ice was left to melt, and precipitate was collected by vacuum filtration, washed with H_2O until neutral. The precipitate was dried in the oven overnight to yield a crude mixture of dibromo PDA as a red solid (28.0 g). Its poor solubility in any solvent left it unable to be characterised, and it was subjected to imidization reaction to synthesise **6** without further purification.

***N-N*-Di(2,6-diisopropylphenyl) 1,7-dibromo-perylene-3,4,9,10-tetracarboxylic diimide[5] 6**

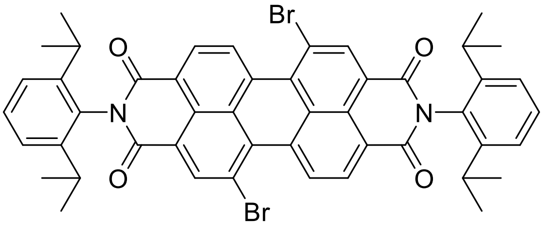

Crude mixture of dibromo PDA (5.90 g, 10.7 mmol, 1 eq) was dissolved in propionic acid (120 mL). 2,6-Diisopropylaniline (4.5 mL, 24 mmol, 2.2 eq) was then added and the reaction mixture was left to stir at 140 °C for four days. On cooling to rt, the resulting mixture was added onto a large beaker of ice, causing the formation of a red precipitate. Any remaining ice was left to melt, and precipitate was collected by vacuum filtration, washed with H_2O until neutral. The red solid was added to CH_2Cl_2 (200 mL), and the resulting suspension was filtered under vacuum, washing with CH_2Cl_2 (3 × 50 mL). The filtrate was concentrated under vacuum and purified by flash column chromatography (SiO_2, 60:40 CH_2Cl_2:n-hexane) to yield dibromo PDI **6** as red solids (2.42 g, 39%).

^{1}H NMR (400 MHz, $CDCl_3$) δ 9.57 (d, *J* = 8.1 Hz, 2H, Ar*H* PDI), 9.03 (s, 2H, Ar*H* PDI), 8.82 (d, *J* = 8.1 Hz, 2H, Ar*H* PDI), 7.53 (t, *J* = 7.8 Hz, 2H, Ar*H* iPr$_2$-Ar*H*), 7.37 (d, *J* = 7.8 Hz, 4H, iPr$_2$-Ar*H*), 2.75 (hept, *J* = 7.0 Hz, 4H, C*H* iPr), 1.20 (d, *J* = 7.0 Hz, 24H, CH(C*H*$_3$)$_2$ iPr).

MS (ESI): m/z calc. for $C_{48}H_{41}Br_2N_2O_4$ $[M+H]^+$: 869.1418; found 869.1443.

Spectroscopic data is in agreement with literature.[5]

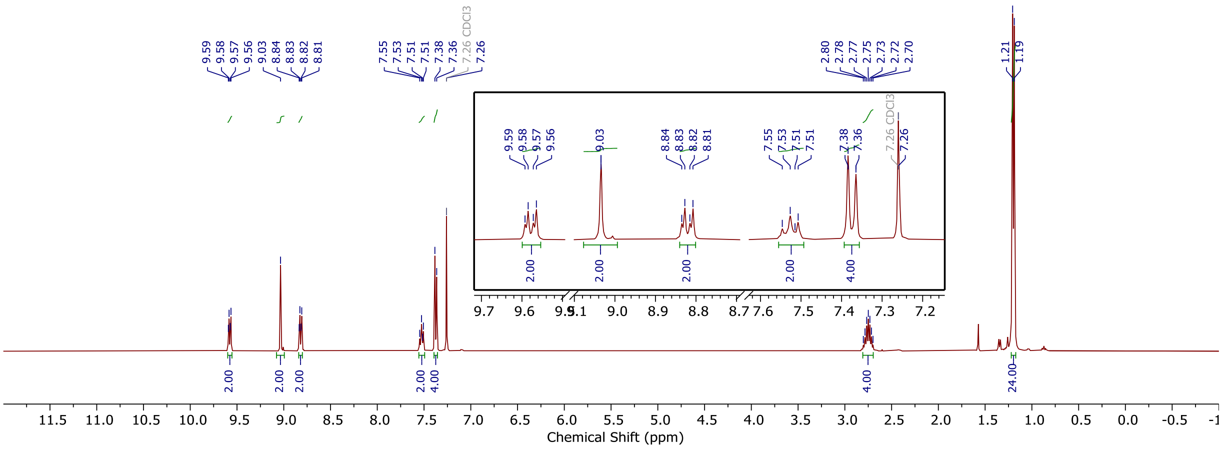

^{1}H NMR spectrum of **6** (400 MHz, $CDCl_3$).

***N-N*-Di(2,6-diisopropylphenyl) 1,7-di((trimethylsilyl)ethynyl)-perylene-3,4,9,10-tetracarboxylic diimide[6] 7**

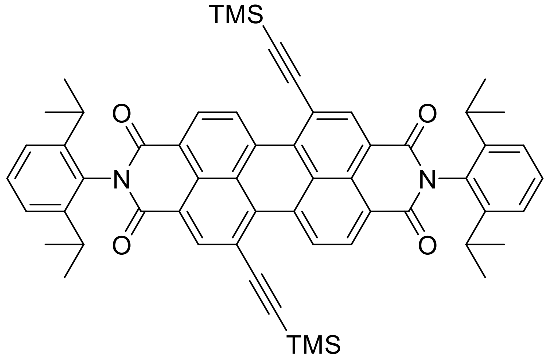

Dibromo PDI **6** (2.40 g, 2.76 mmol, 1 eq) was dissolved in triethylamine (125 mL) and degassed by bubbling a stream of N_2 for 15 min. After which, copper iodide (58.0 mg, 0.303 mmol, 0.11 eq), $PdCl_2(PPh_3)_2$ (116 mg, 0.166 mmol, 0.06 eq), and trimethylsilylacetylene (1.95 mL, 13.8 mmol, 5 eq) was added, and the reacting mixture was left to stir at 60°C for 16 h under an atmosphere of N_2. On cooling to rt, the resulting mixture was quenched with 10M HCl (100 mL). The product was extracted with CH_2Cl_2 (3 × 100 mL), washed with H_2O until neutral, dried with $MgSO_4$ and concentrated *in vacuo*. Purification via flash column chromatography (SiO_2, 60:40 CH_2Cl_2:n-hexane) furnished TMS protected PDI **7** as a red solid (2.28 g, 99%).

^{1}H NMR (400 MHz, $CDCl_3$) δ 10.30 (d, *J* = 8.2 Hz, 2H, Ar*H* PDI), 8.94 (s, 2H, Ar*H* PDI), 8.75 (d, *J* = 8.2 Hz, 2H, Ar*H* PDI), 7.52 (t, *J* = 7.8 Hz, 2H, iPr_2-Ar*H*), 7.37 (d, *J* = 7.8 Hz, 4H, iPr_2-Ar*H*), 2.82 – 2.68 (m, 4H, C*H* iPr), 1.19 (app dd, *J* = 6.8, 1.0 Hz, 24H, CH(CH_3)$_2$ iPr), 0.40 (s, 18H, Si(CH_3)$_3$).

MS (ESI): m/z calc. for $C_{58}H_{59}N_2O_4Si_2$ $[M+H]^+$: 903.4013; found: 903.4020

Spectroscopic data is in agreement with literature.[6]

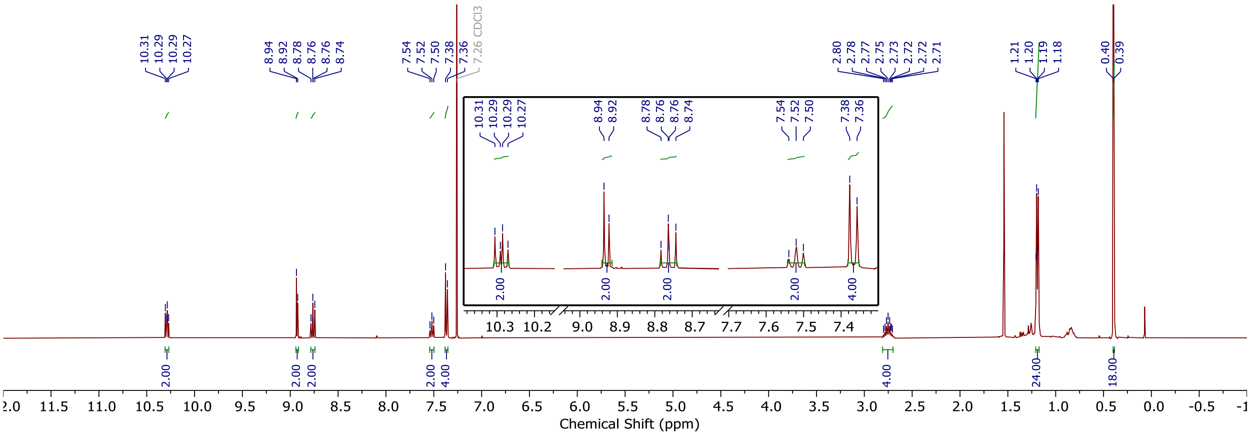

^{1}H NMR spectrum of **7** (400 MHz, $CDCl_3$).

_N-N_-Di(2,6-diisopropylphenyl) 1,7-diethynyl-perylene-3,4,9,10-tetracarboxylic diimide[7] 8

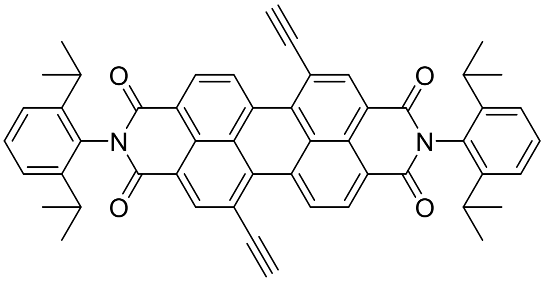

TMS protected PDI **7** (2.28 g, 2.52 mmol, 1 eq) was dissolved in CH_2Cl_2/MeOH (1:1 v/v, 300 mL). Potassium carbonate (1.05 g, 7.57 mmol, 3 eq) was then added and the reaction mixture was stirred at rt for 1 h. After which, the resulting mixture was added to H_2O (200 mL) extracted with CH_2Cl_2, washed with 1 M HCl (200 mL) and H_2O (200 mL). The organic layer was dried with $MgSO_4$ and concentrated *in vacuo* to furnished dialkynyl PDI **8** as a dark red solid (1.93 g, quant.).

^{1}H NMR (400 MHz, $CDCl_3$) δ 10.11 (d, *J* = 8.2 Hz, 2H) , Ar*H* PDI, 8.96 (s, 2H, Ar*H* PDI), 8.83 (d, *J* = 8.2 Hz, 2H, Ar*H* PDI), 7.52 (t, *J* = 7.8 Hz, 2H, iPr$_2$-Ar*H*), 7.37 (d, *J* = 7.8 Hz, 4H, iPr$_2$-Ar*H*), 3.84 (s, 2H, C≡C*H*), 2.83 – 2.68 (m, 4H, C*H* iPr), 1.19 (d, *J* = 6.8 Hz, 24H, CH(C*H*$_3$)$_2$ iPr).

MS (ESI): m/z calc. for $C_{52}H_{43}N_2O_4$ $[M+H]^+$: 759.3223; found 759.3251

Spectroscopic data is in agreement with literature.[7]

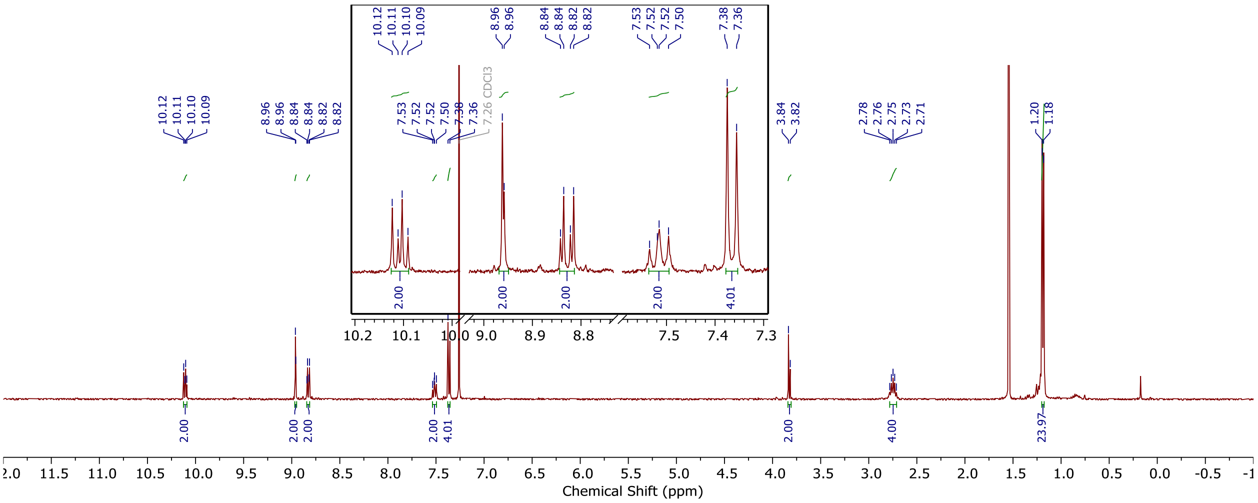

^{1}H NMR spectrum of **8** (400 MHz, $CDCl_3$).

General procedure for compounds 9-12:

A: Formation of alkyl bromide
The dihydroxy starting material and dibromo alkane were added to a suspension of potassium carbonate in acetone and left to stir at 60 °C for three days. After which, the reaction mixture was left to cool to room temperature and filtered. The filtrate was concentrated *in vacuo* and purified via flash column chromatography (100:0 → 60:40 n-hexane:CH_2Cl_2).

B: Formation of organic azides
Halide substrate and NaN_3 were dissolved in DMF and left to stir at 75 °C for 16 h. The reaction mixture was quenched with H_2O. If a precipitate was formed, the solid product was collected by vacuum filtration, washed with H_2O and left under suction for 15 min to dry. If no precipitate was formed, the reaction mixture was extracted with Et_2O, washed with H_2O (× 4), brine, then dried with $MgSO_4$ and concentrated *in vacuo*.

1,4-Di(4-bromobutoxy)benzene[8] 9a

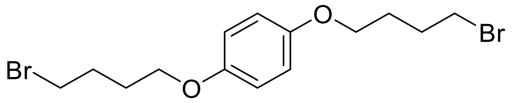

1,4-Dibromobutane (0.87 mL, 7.32 mmol, 3.5 eq) was added to a stirring suspension of potassium carbonate (1.5 g, 10.93 mmol, 5.3 eq) and hydroquinone (227 mg, 2.06 mmol, 1 eq) in acetone (60 mL). The resulting mixture was left to stir at 60 °C for three days. Upon cooling to room temperature, the reaction mixture was filtered, and the filtrate was concentrated *in vacuo* to an orange oil. The oil was diluted in n-hexane (50 mL) and filtered again, washing with more n-hexane (2 × 10 mL). The filtrate was set aside overnight in the fridge to crystallise. The off-white crystals were collected, and the mother liquor was concentrated *in vacuo*, redissolved in a minimal amount of hot ethanol, and set aside overnight to yield a second crop of off-white crystals. These crystals were combined with the first batch to yield **9a** (199 mg, 25%).

^{1}H NMR (400 MHz, $CDCl_3$) δ 6.81 (s, Ar*H*, 4H), 3.94 (t, *J* = 6.1 Hz, 4H, C*H*$_2$), 3.49 (t, *J* = 6.6 Hz, 4H, C*H*$_2$), 2.10 – 1.99 (m, 4H, C*H*$_2$), 1.97 – 1.85 (m, C*H*$_2$, 4H). Spectroscopic data is in agreement with literature.[8]

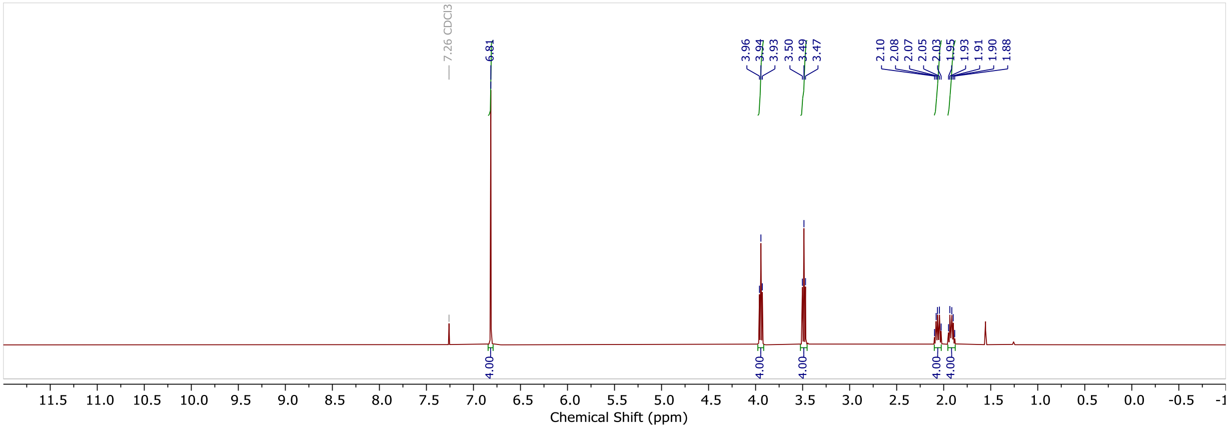

^{1}H NMR spectrum of **9a** (400 MHz, $CDCl_3$).

P-2,2'-bis(4-bromobutoxy)-1,1'-binaphthalene[9] *P*-10a

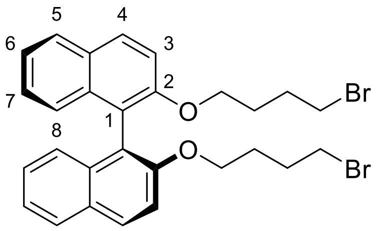

General procedure **A** was followed with ***P***-BINOL (1.00 g, 3.49 mmol, 1 eq), 1,4-dibromobutane (6.3 mL, 52.4 mmol, 15 eq), potassium carbonate (2.90 g, 21.0 mmol, 6 eq) in acetone (10 mL), to yield ***P*-10a** as a colourless oil (0.702 g, 36%). The major side product was identified to be the [1+1] macrocyclic adduct. The dibromide ***P*-10a** was further reacted to form the diazide ***P*-12a** without further characterization.

^{1}H NMR (400 MHz, $CDCl_3$) δ 7.95 (d, *J* = 9.0 Hz, 2H, C(4)*H*), 7.87 (dd, *J* = 8.2, 1.0 Hz, 2H, C(5)*H*), 7.41 (d, *J* = 9.0 Hz, 2H, C(3)*H*), 7.33 (ddd, *J* = 8.2, 6.6, 1.0 Hz, 2H, C(6)*H*), 7.22 (ddd, *J* = 8.3, 6.6, 1.0 Hz, 2H, C(7)*H*), 7.16 (dd, *J* = 8.3, 1.0 Hz, 2H, C(8)*H*), 4.04 (ddd, *J* = 9.4, 6.5, 4.8 Hz, 2H, OCH_AH_B), 3.93 (ddd, *J* = 9.4, 6.9, 4.8 Hz, 2H, OCH_AH_B), 2.91 (td, *J* = 6.8, 2.4 Hz, 4H, $BrCH_2$), 1.64 – 1.48 (m, 4H, CH_2), 1.45 – 1.32 (m, 4H, CH_2).

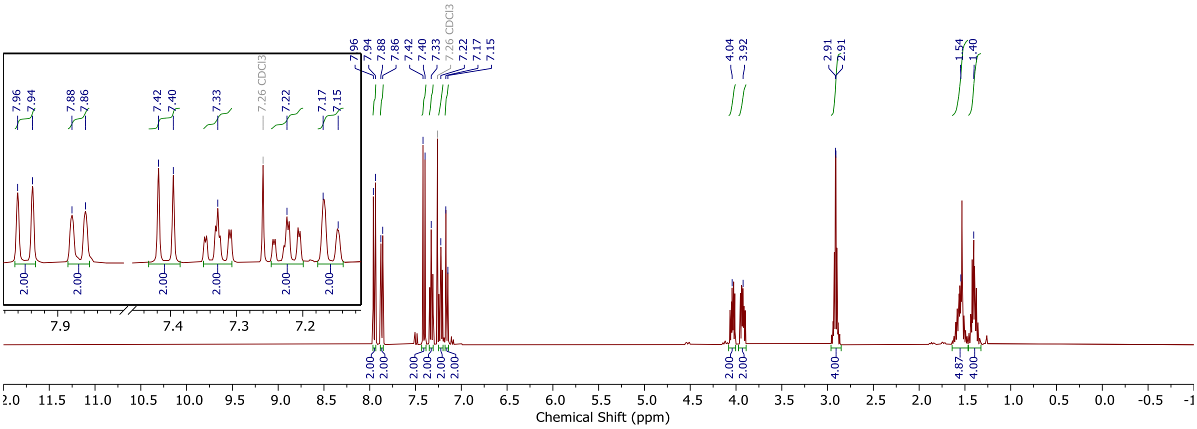

^{1}H NMR spectrum of ***P*-10a** (400 MHz, $CDCl_3$).

***P*-2,2'-bis((6-bromohexyl)oxy)-1,1'-binaphthalene[10] *P*-10b**

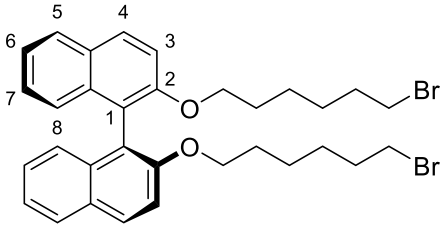

General procedure **A** was followed with ***P***-BINOL (1.00 g, 3.49 mmol, 1 eq), 1,6-dibromohexane (8.1 mL, 52.4 mmol, 15 eq), potassium carbonate (2.90 g, 21.0 mmol, 6 eq) in acetone (10 mL), to yield ***P*-10b** as a colourless oil (1.47g, 69%). The major side product was identified to be the [1+1] macrocyclic adduct.

The reaction was repeated with its enantiomer ***M***- to yield ***M*-10b** with matching results and spectroscopic data.

^{1}H NMR (400 MHz, $CDCl_3$) δ = 7.94 (d, *J* = 8.9 Hz, 2H, C(4)*H*), 7.86 (dd, *J* = 8.3, 1.2 Hz, 2H, C(5)*H*), 7.41 (d, *J* = 8.9 Hz, 2H, C(3)*H*), 7.32 (ddd, *J* = 8.3, 6.5, 1.0 Hz, 2H, C(6)*H*), 7.21 (ddd, *J* = 8.2, 6.5, 1.2 Hz, 2H, C(7)*H*), 7.16 (ddd, *J* = 8.2, 1.0, 0.5 Hz, 2H, C(8)*H*), 4.08 – 3.94 (m, 2H, OCH_AH_B), 3.88 (ddd, *J* = 9.3, 7.1, 5.4 Hz, 2H, OCH_AH_B), 3.15 (t, *J* = 7.0 Hz, 4H, $BrCH_2$), 1.54 – 1.45 (m, 4H, CH_2), 1.45 – 1.32 (m, 4H, CH_2), 1.05 (dtt, *J* = 9.0, 6.8, 3.2 Hz, 4H, CH_2), 0.90 – 0.75 (m, 4H, CH_2).

Spectroscopic data is in agreement with literature.[10]

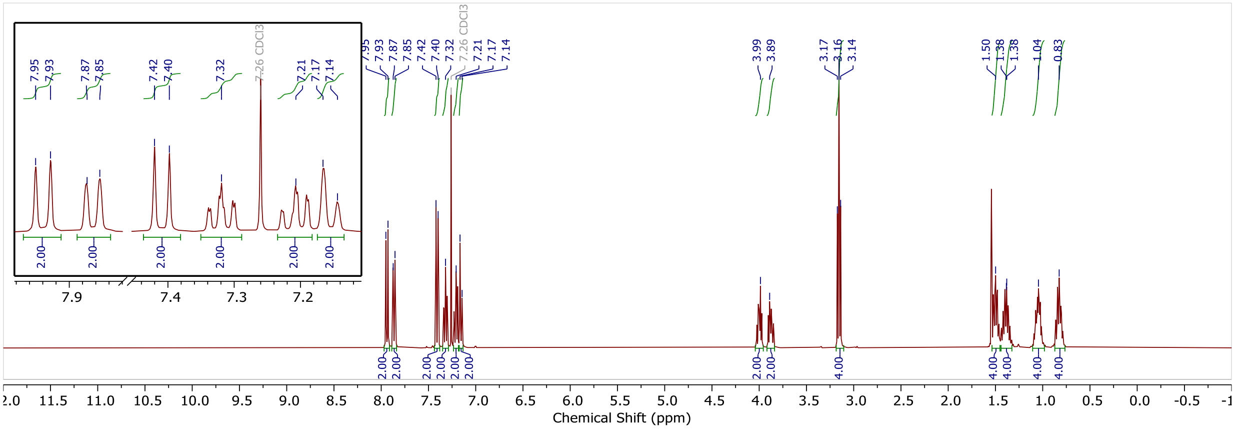

^{1}H NMR spectrum of ***P*-10b** (400 MHz, $CDCl_3$).

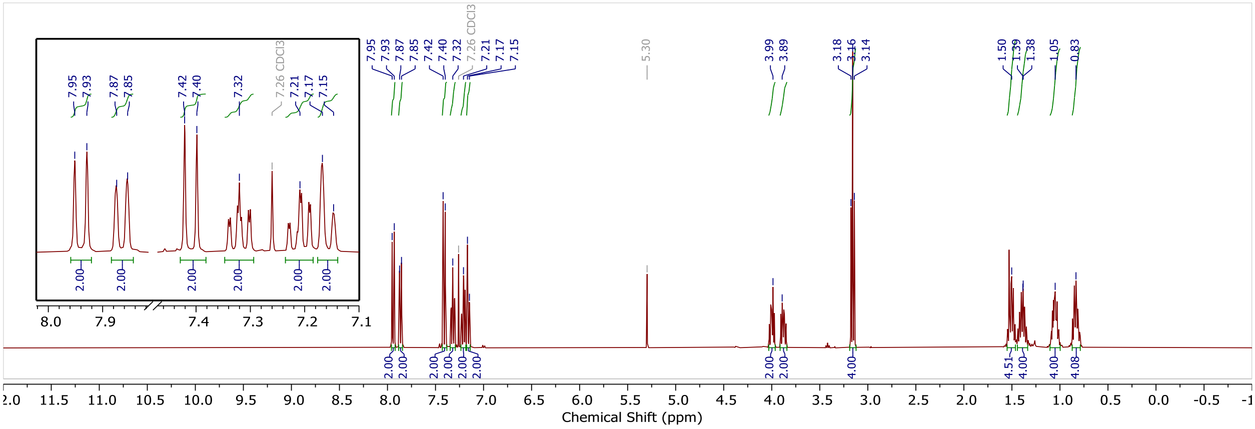

^{1}H NMR spectrum of ***M*-10b** (400 MHz, $CDCl_3$).

1,4-Di(4-azidobutoxy)benzene[11] 11a

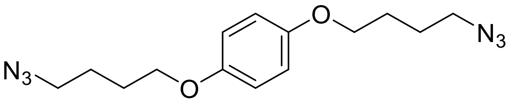

General procedure **B** was followed with dibromo **9a** (199 mg, 0.524 mmol, 1 eq) and NaN_3 (85.1 mg, 1.31 mmol, 2.5 eq) in DMF (10 mL) to yield azide **11a** an off-white solid (160 mg, quant.)

^{1}H NMR (400 MHz, $CDCl_3$) δ 6.83 (s, 4H, Ar*H*), 3.94 (t, *J* = 5.8 Hz, 4H, C*H*$_2$), 3.36 (t, *J* = 6.5 Hz, 4H, C*H*$_2$), 1.95 – 1.71 (m, C*H*$_2$, 8H).

MS (ASAP): m/z calc. for $C_{14}H_{21}N_6O_2$ $[M]^+$: 304.1648; found 304.1650.

Spectroscopic data is in agreement with literature.[11]

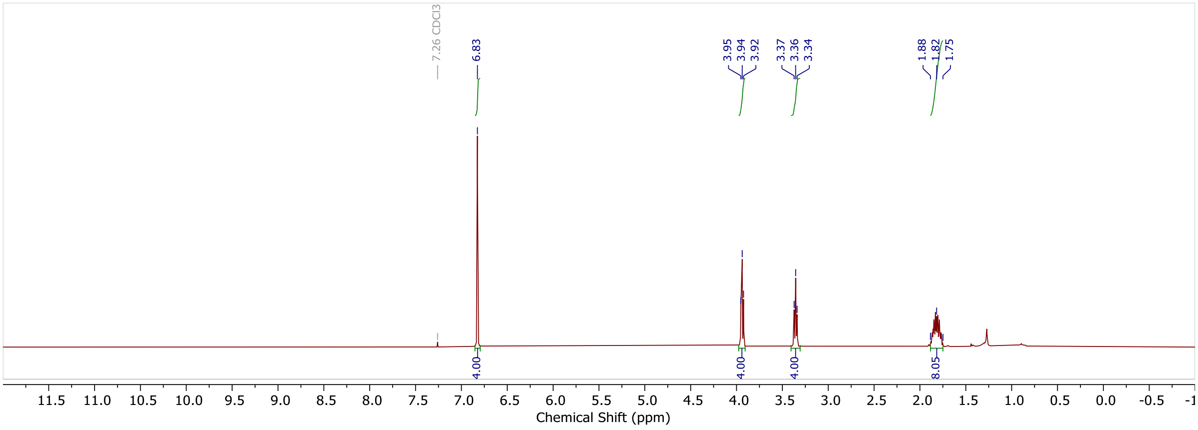

^{1}H NMR spectrum of **11a** (400 MHz, $CDCl_3$).

1,4-Di(6-azidohexoxy)benzene 11b

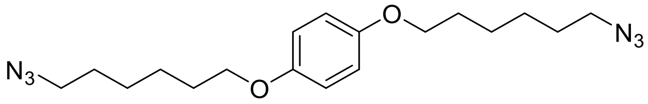

General procedure **A** was followed with hydroquinone (1.00 g, 9.08 mmol, 1 eq), 1,6-dibromohexane (14 mL, 90.8 mmol, 10 eq), potassium carbonate (7.53 g, 54.5 mmol, 6 eq) in acetone (20 mL), to yield 1,4-di(6-bromohexoxy)benzene **9b** as a white solid (3.13 g, 79%).

General procedure **B** was then followed with dibromo **9b** (3.12 g, 7.15 mmol, 1 eq) and NaN_3 (1.16 g, 17.9 mmol, 2.5 eq) in DMF (50 mL) to yield azide **11b** as a yellow oil that solidified into a cream white solid (2.55 g, 99%).

^{1}H NMR (400 MHz, $CDCl_3$) δ = 6.82 (s, 4H, Ar*H*), 3.91 (t, *J* = 6.4 Hz, 4H, C*H*$_2$), 3.28 (t, *J* = 6.9 Hz, 4H, C*H*$_2$), 1.77 (dq, *J* = 7.7, 6.3 Hz, 4H, C*H*$_2$), 1.63 (p, *J* = 7.0 Hz, 4H, C*H*$_2$), 1.55 – 1.38 (m, 8H, C*H*$_2$).

MS (ASAP): m/z calc. for $C_{18}H_{28}N_6O_2$ $[M]^+$: 360.2274; found 360.2284

Spectroscopic data is in agreement with literature.[11]

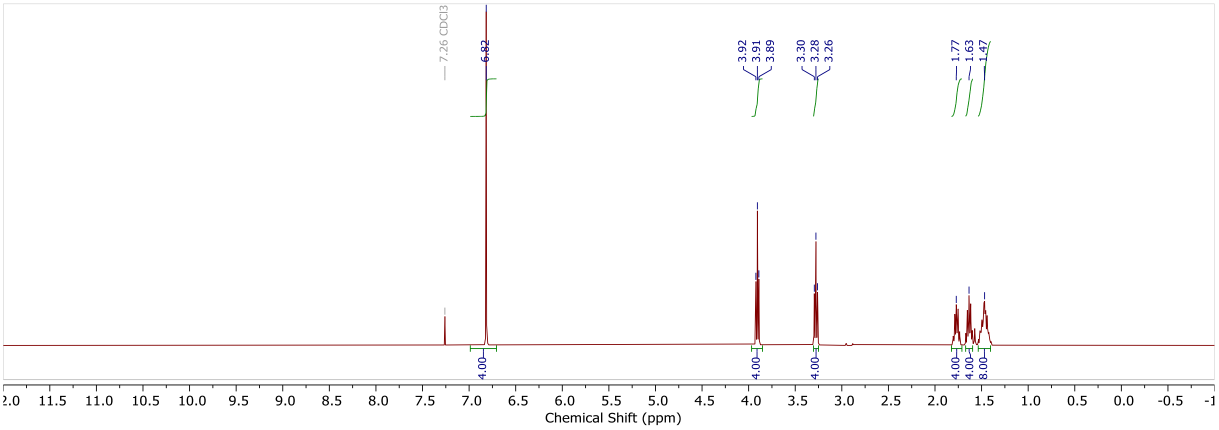

^{1}H NMR spectrum of **11b** (400 MHz, $CDCl_3$).

P-2,2'-bis(4-azidobutoxy)-1,1'-binaphthalene *P*-12a

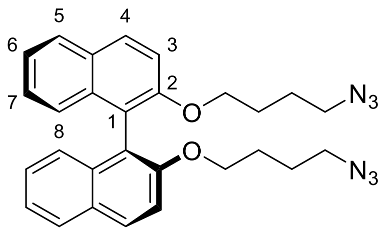

General procedure **B** was followed with dibromo ***P*-10a** (680 mg, 1.22 mmol, 1 eq) and NaN_3 (200 mg, 3.06 mmol, 2.5 eq) in DMF (10 mL) to yield ***P*-12a** as a yellow oil that solidified into a cream white solid (575 mg, 97 %).

^{1}H NMR (400 MHz, $CDCl_3$) δ 7.96 (d, *J* = 9.0 Hz, 2H, C(4)*H*), 7.88 (d, *J* = 8.0 Hz, 2H, C(5)*H*), 7.41 (d, *J* = 9.0 Hz, 2H, C(3)*H*), 7.34 (ddd, *J* = 8.0, 6.5, 1.3 Hz, 2H, C(6)*H*), 7.23 (ddd, *J* = 8.1,

6.6, 1.3 Hz, 2H, C(7)*H*), 7.17 (dd, *J* = 8.1, 1.3 Hz, 2H, C(8)*H*), 4.02 (ddd, *J* = 9.3, 6.6, 4.9 Hz, 2H, OCH_AH_B), 3.91 (ddd, *J* = 9.4, 7.0, 4.8 Hz, 2H, OCH_AH_B), 2.74 (app td, *J* = 7.0, 3.3 Hz, 4H, N_3CH_2), 1.46 (ttd, *J* = 14.2, 7.0, 4.9 Hz, 4H, CH_2), 1.18 – 1.07 (m, 4H, CH_2).

^{13}C NMR (101 MHz, $CDCl_3$) δ 154.4, 134.2, 129.5, 129.5, 128.1, 126.4, 125.5, 123.8, 120.9, 115.9, 69.3, 50.8, 26.5, 25.5.

MS (ASAP): m/z calc. for $C_{28}H_{28}N_6O_2$ $[M]^+$: 480.2274; found 480.2283.

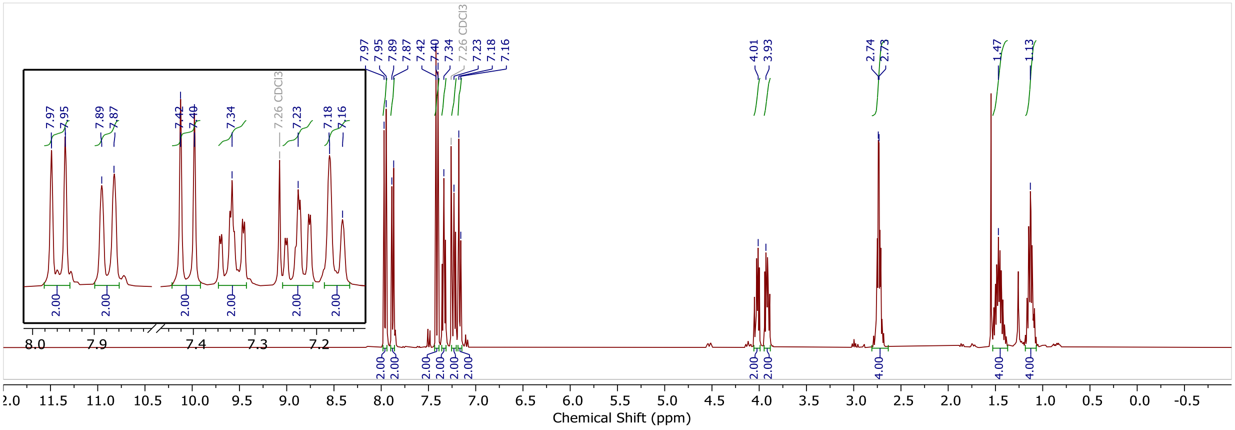

^1H NMR spectrum of ***P*-12a** (400 MHz, $CDCl_3$).

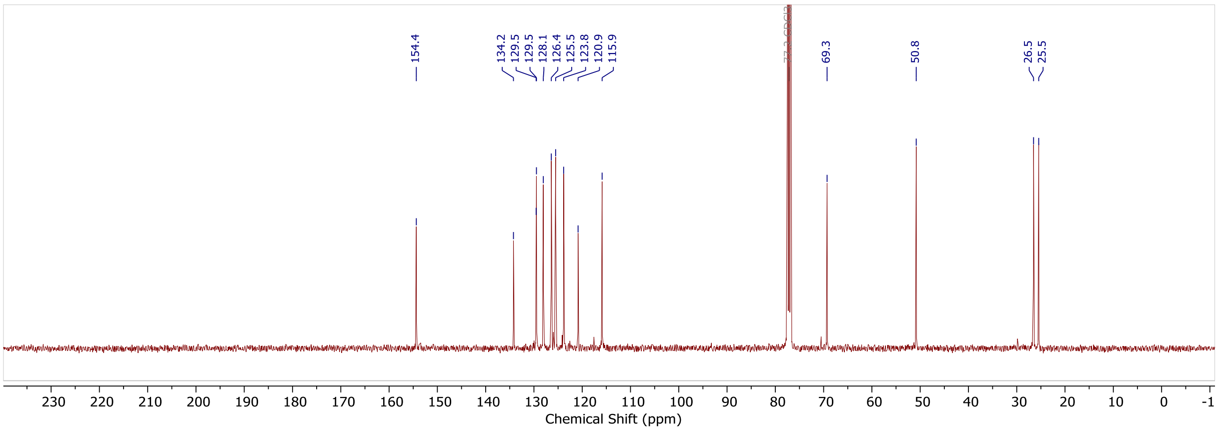

^{13}C NMR spectrum of ***P*-12a** (101 MHz, $CDCl_3$).

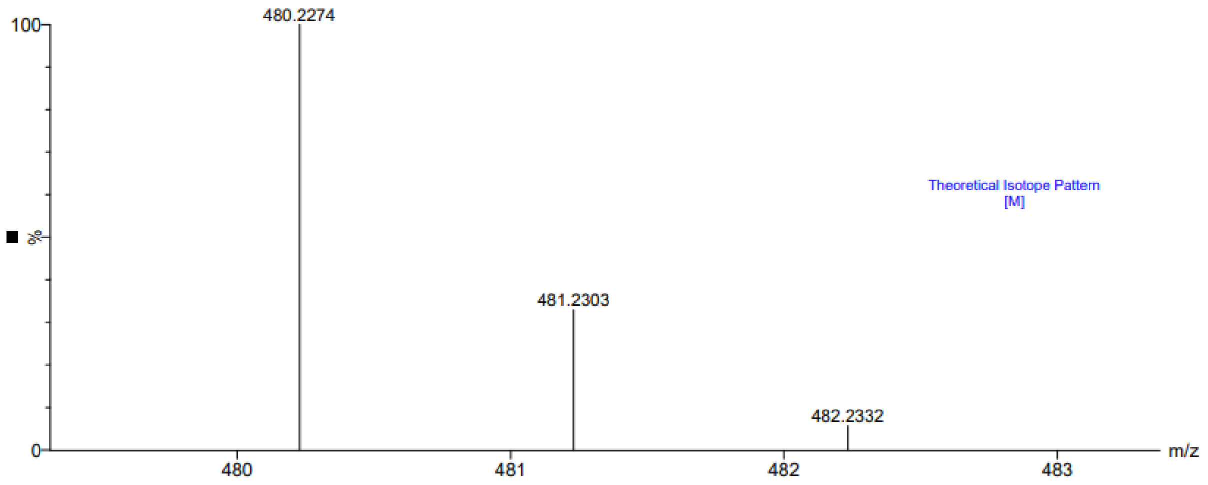

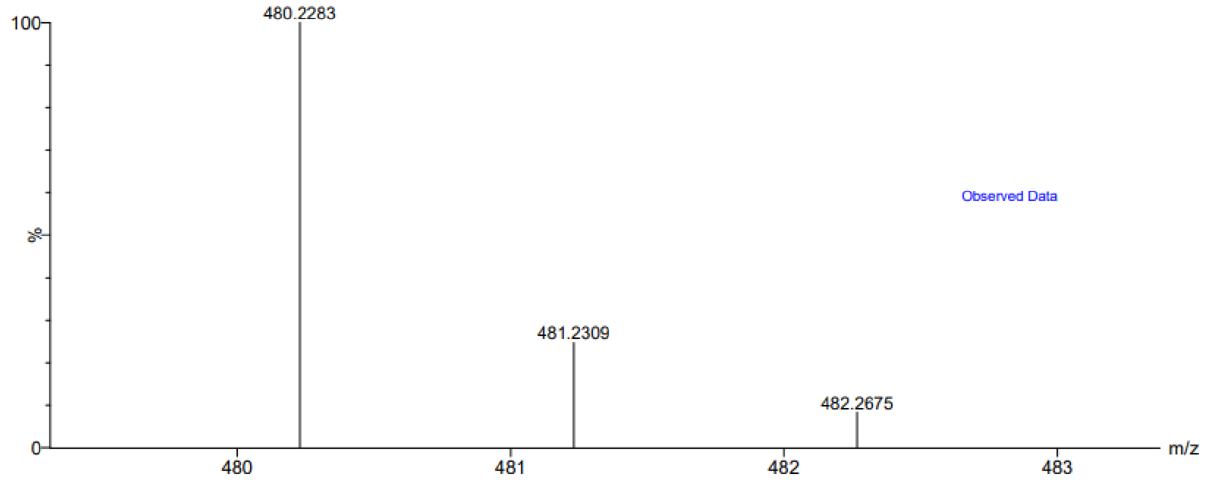

Calculated (top) and observed (bottom) ESI MS data for compound ***P*-12a**.

P-2,2'-bis((6-azidohexyl)oxy)-1,1'-binaphthalene *P*-12b

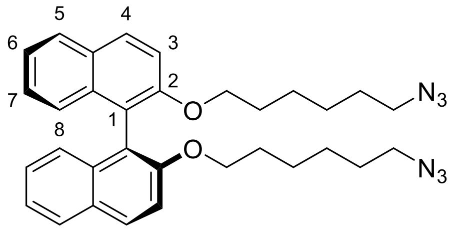

General procedure **B** was followed with dibromo ***P*-10b** (1.47 g, 2.40 mmol, 1 eq) and NaN_3 (390 mg, 6.00 mmol, 2.5 eq) in DMF (20 mL) to yield ***P*-12b** as a yellow oil (1.29 g, quant.).

The reaction was repeated with its enantiomer ***M*-10b** to yield ***M*-12b** with near identical results and spectroscopic data.

^{1}H NMR (400 MHz, $CDCl_3$) δ 7.94 (d, *J* = 8.9 Hz, 2H, C(4)*H*), 7.86 (dd, *J* = 8.3, 1.1 Hz, 2H, C(5)*H*), 7.41 (d, *J* = 8.9 Hz, 2H, C(3)*H*), 7.32 (ddd, *J* = 8.3, 6.5, 1.4 Hz, 2H, C(6)*H*), 7.21 (ddd, *J* = 8.1, 6.5, 1.1 Hz, 2H, C(7)*H*), 7.16 (dd, *J* = 8.1, 1.4 Hz, 2H, C(8)*H*), 4.00 (dt, *J* = 9.3, 5.8 Hz, 2H, OCH_AH_B), 3.88 (ddd, *J* = 9.3, 7.0, 5.5 Hz, 2H, OCH_AH_B), 3.00 (t, *J* = 7.1 Hz, 4H,

N_3CH_2), 1.45 – 1.32 (m, 4H, OCH_2CH_2), 1.31 – 1.19 (m, 4H, NCH_2CH_2), 0.99 (dddd, J = 13.5, 11.3, 6.8, 2.4 Hz, 4H, $N(CH_2)_2CH_2$), 0.90 – 0.77 (m, 4H, $O(CH_2)_2CH_2$).

^{13}C NMR (101 MHz, $CDCl_3$) δ 154.6, 134.3, 129.5, 129.2, 128.0, 126.2, 125.6, 123.7, 121.0, 116.1, 69.7, 51.3, 29.3, 28.6, 26.2, 25.3.

MS (ASAP): m/z calc. for $C_{32}H_{37}N_6O_2$ $[M+H]^+$: 537.2978; found 537.2974

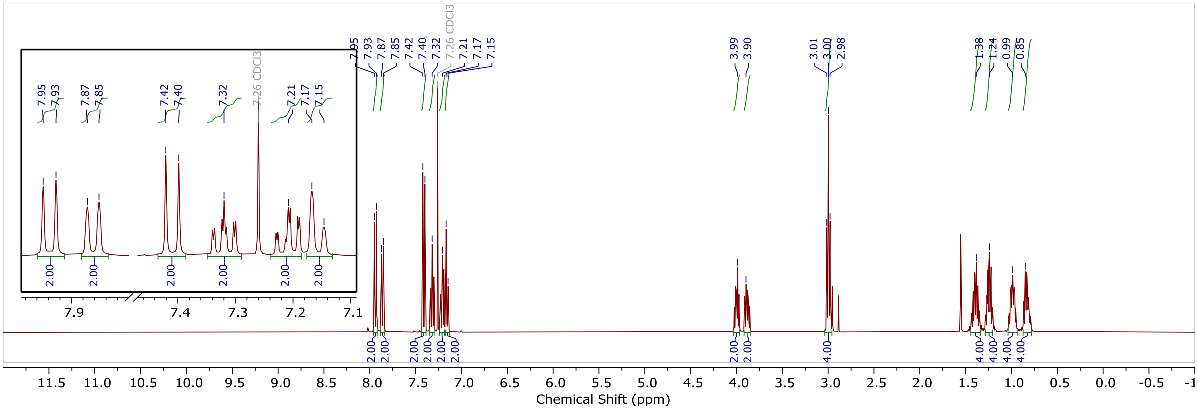

^{1}H NMR spectrum of ***P*-12b** (400 MHz, $CDCl_3$).

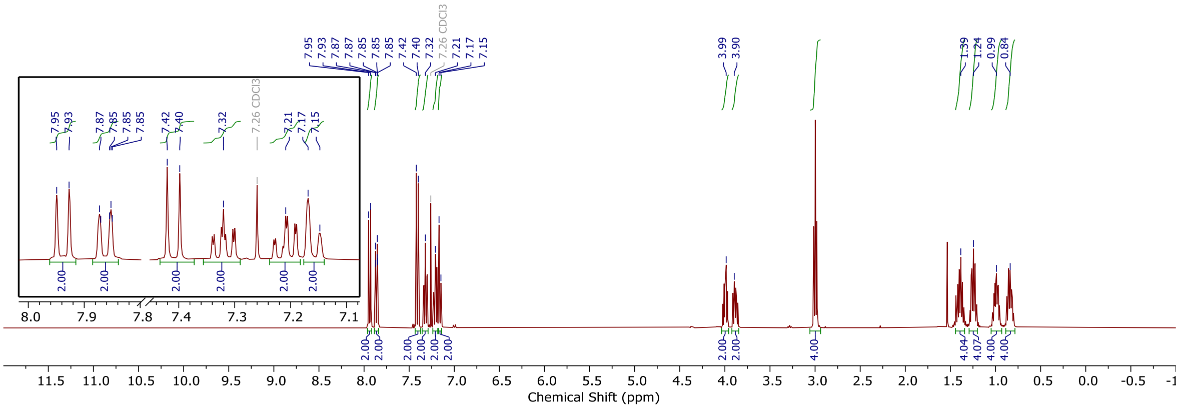

^{1}H NMR spectrum of ***M*-12b** (400 MHz, $CDCl_3$).

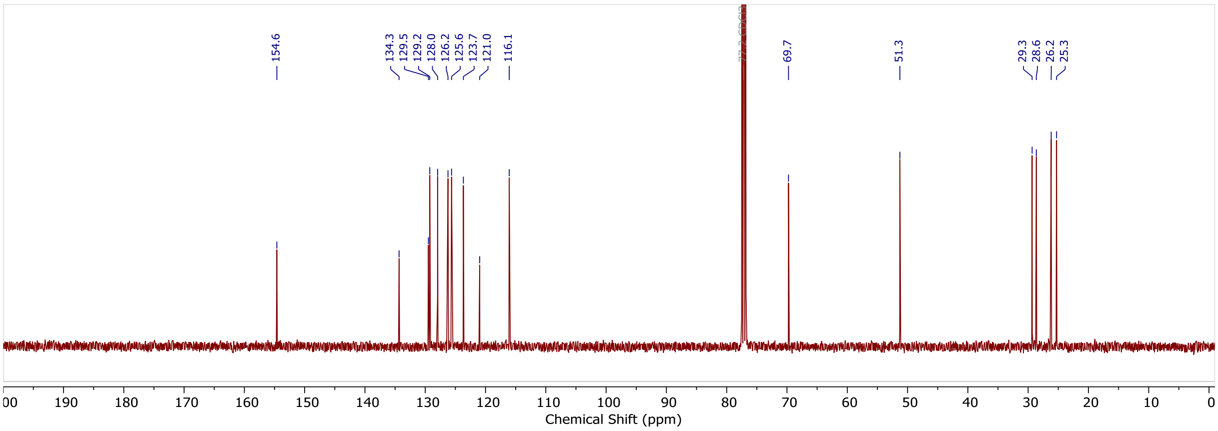

^{13}C NMR spectrum of ***P*-12b** (101 MHz, $CDCl_3$).

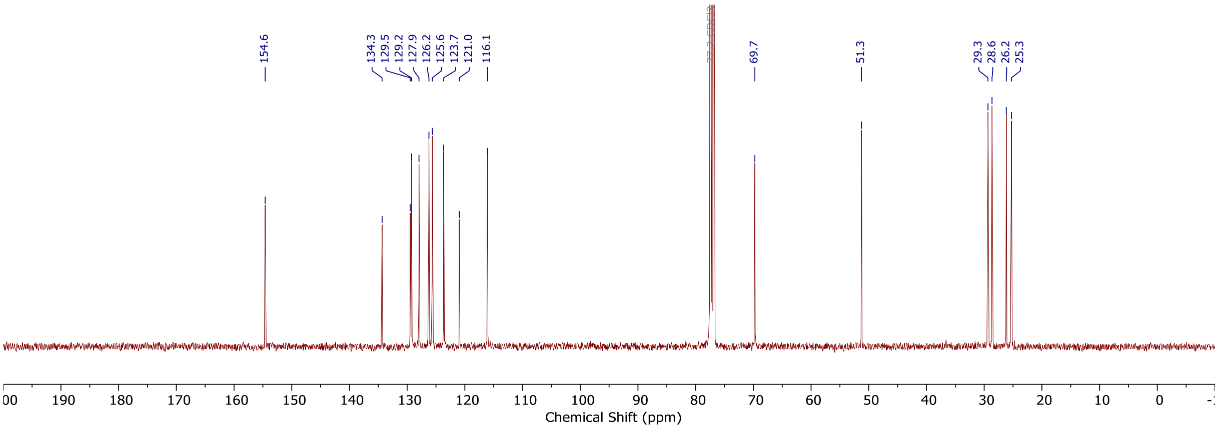

^{13}C NMR spectrum of ***M*-12b** (101 MHz, $CDCl_3$).

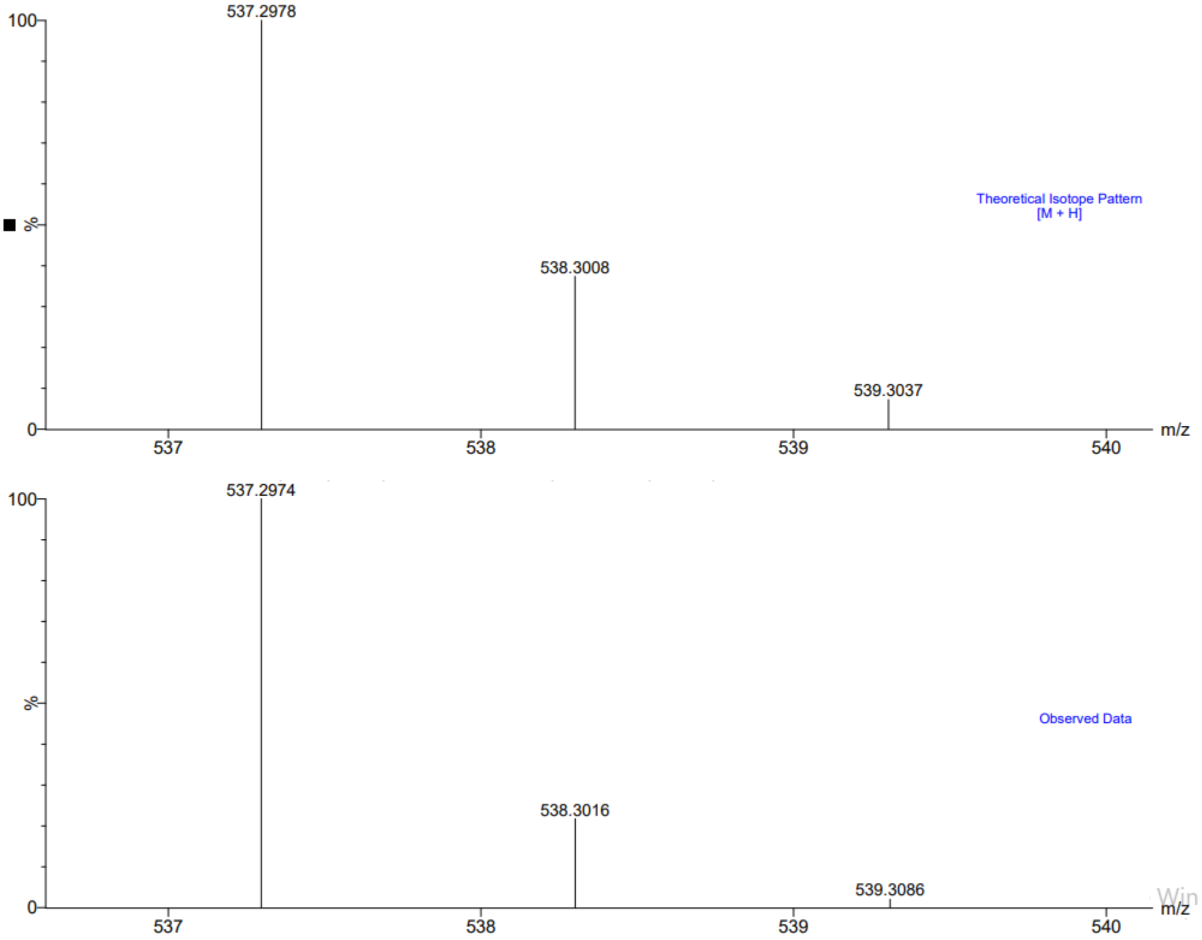

Calculated (top) and observed (bottom) ESI MS data for compound ***P*-12b**.

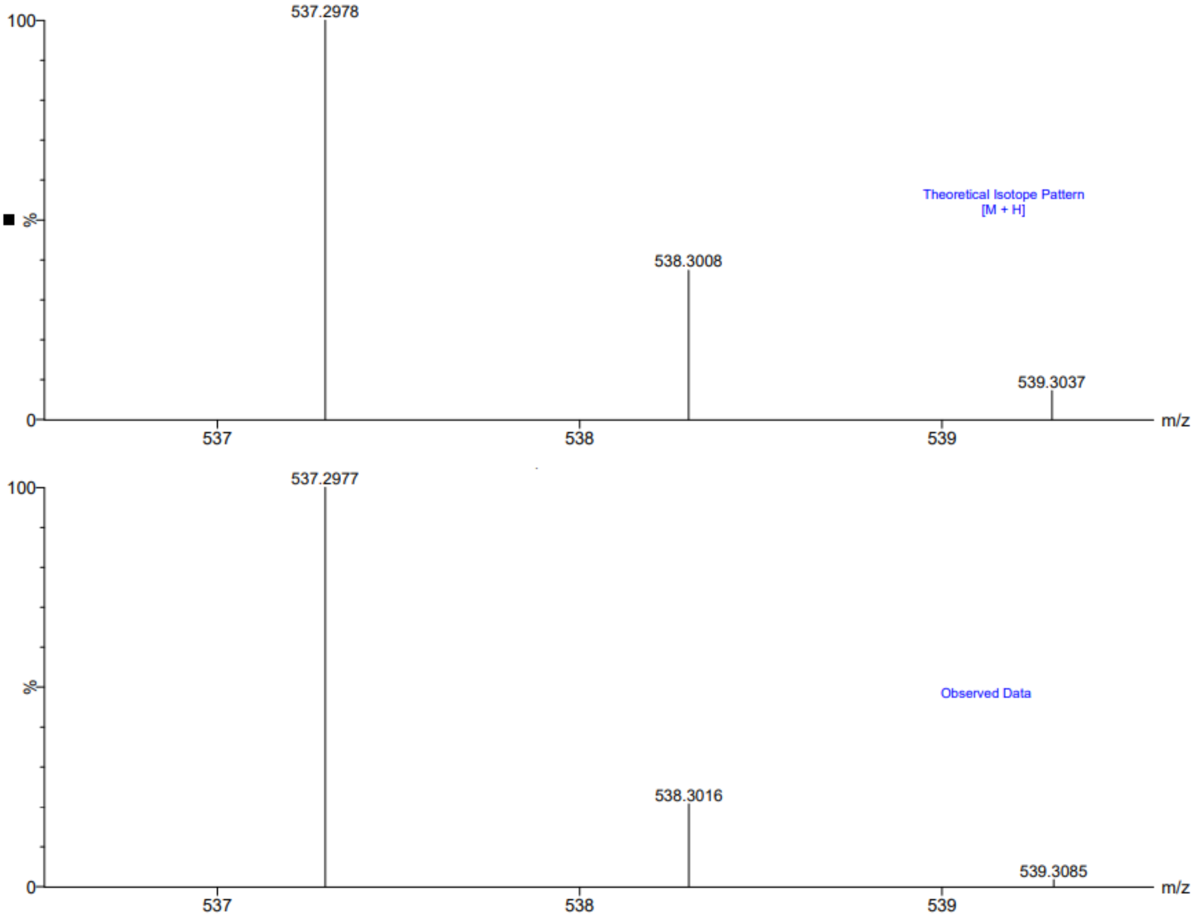

Calculated (top) and observed (bottom) ESI MS data for compound ***M*-12b**.

P-2,2'-bis((8-azidooctyl)oxy)-1,1'-binaphthalene *P*-12c

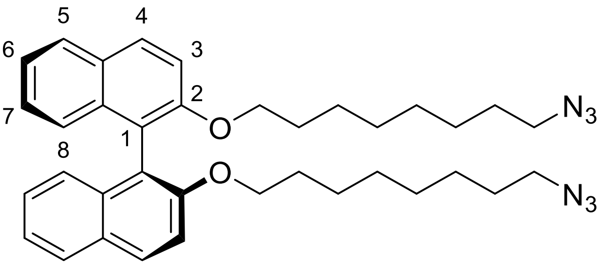

General procedure **A** was followed with ***P***-BINOL (1.00 g, 3.49 mmol, 1 eq), 1,8-dibromooctane (9.7 mL, 52.4 mmol, 15 eq), potassium carbonate (2.90 g, 21.0 mmol, 6 eq) in acetone (10 mL), to yield ***P*-10c** as a colourless oil (2.00 g, 86%). The major side product was identified to be the [1+1] macrocyclic adduct.

General procedure **B** was followed with dibromo ***P*-10c** (2.00 g, 2.99 mmol, 1 eq) and NaN_3 (486 mg, 7.48 mmol, 2.5 eq) in DMF (30 mL) to yield ***P*-12c** as a yellow oil (1.77 g, quant.).

^{1}H NMR (400 MHz, $CDCl_3$) δ 7.92 (d, *J* = 9.0 Hz, 2H, C(4)*H*), 7.85 (dd, *J* = 8.2, 1.3 Hz, 2H, C(5)*H*), 7.40 (d, *J* = 9.0 Hz, 2H, C(3)*H*), 7.30 (ddd, *J* = 8.2, 6.6, 1.4 Hz, 2H, C(6)*H*), 7.20 (ddd, *J* = 8.2, 6.6, 1.3 Hz, 2H, C(7)*H*), 7.15 (dd, *J* = 8.2, 1.4 Hz, 2H, C(8)*H*), 3.97 (dt, *J* = 9.3, 6.2 Hz, 2H, OCH_AH_B), 3.89 (dt, *J* = 9.3, 6.4 Hz, 2H, OCH_AH_B), 3.21 (t, *J* = 7.0 Hz, 4H, N_3CH_2), 1.55 – 1.47 (m, 4H, NCH_2CH_2), 1.44 – 1.32 (m, 4H, OCH_2CH_2), 1.22 – 1.12 (m, 4H, $N(CH_2)_2CH_2$), 1.10 – 0.92 (m, 8H, CH_2), 0.89 (ddd, *J* = 14.2, 7.9, 4.4 Hz, 4H, $O(CH_2)_2CH_2$).

^{13}C NMR (101 MHz, $CDCl_3$) δ 154.7, 134.4, 129.4, 129.2, 127.9, 126.2, 125.7, 123.5, 120.9, 116.0, 69.9, 51.6, 29.5, 29.0, 28.9, 26.6, 25.6.

MS (ASAP): m/z calc. for $C_{36}H_{44}N_6O_2$ $[M+H]^+$: 593.3604; found 593.3614

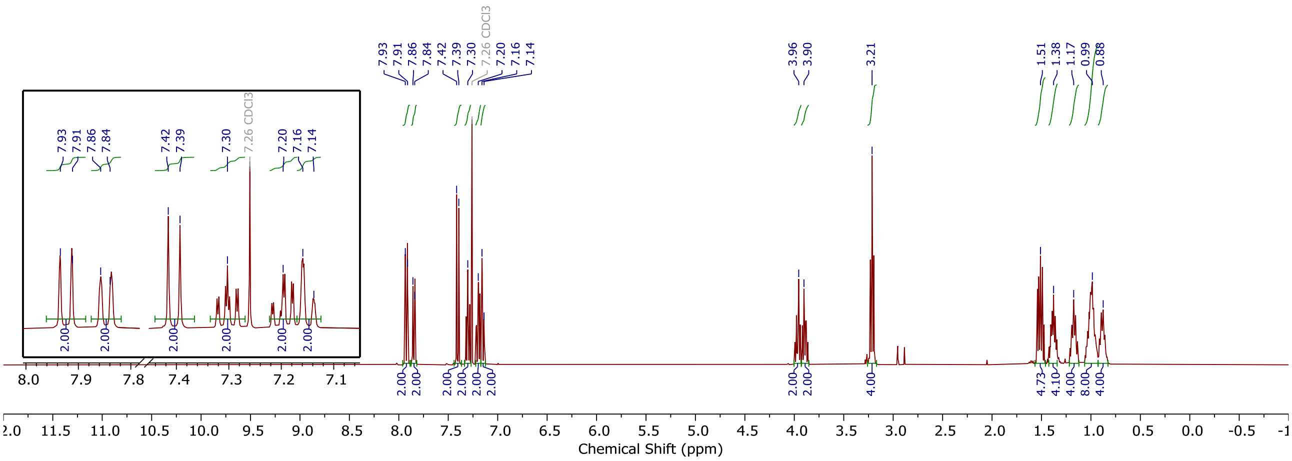

^{1}H NMR spectrum of ***P*-12c** (400 MHz, $CDCl_3$).

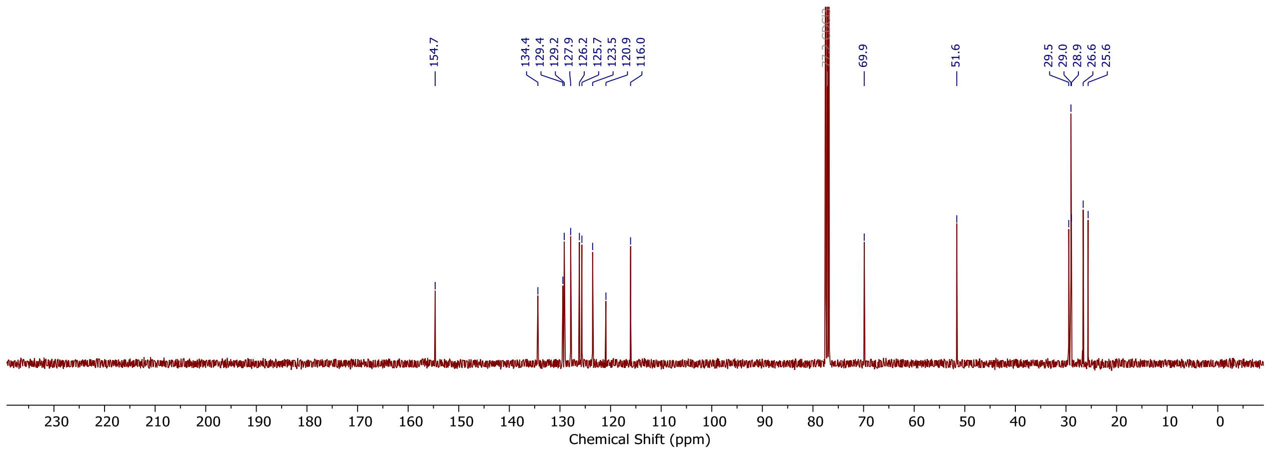

^{13}C NMR spectrum of ***P*-12c** (101 MHz, $CDCl_3$).

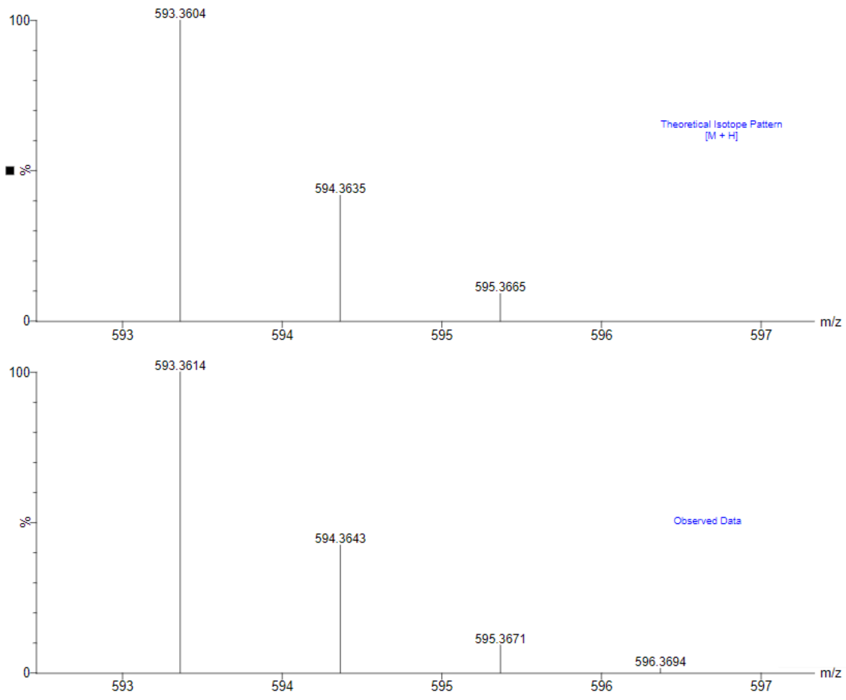

Calculated (top) and observed (bottom) ESI MS data for compound ***P*-12c**.

3. Further NMR Spectroscopy Experiments

3.1. ¹H–¹H NOESY NMR Spectroscopy

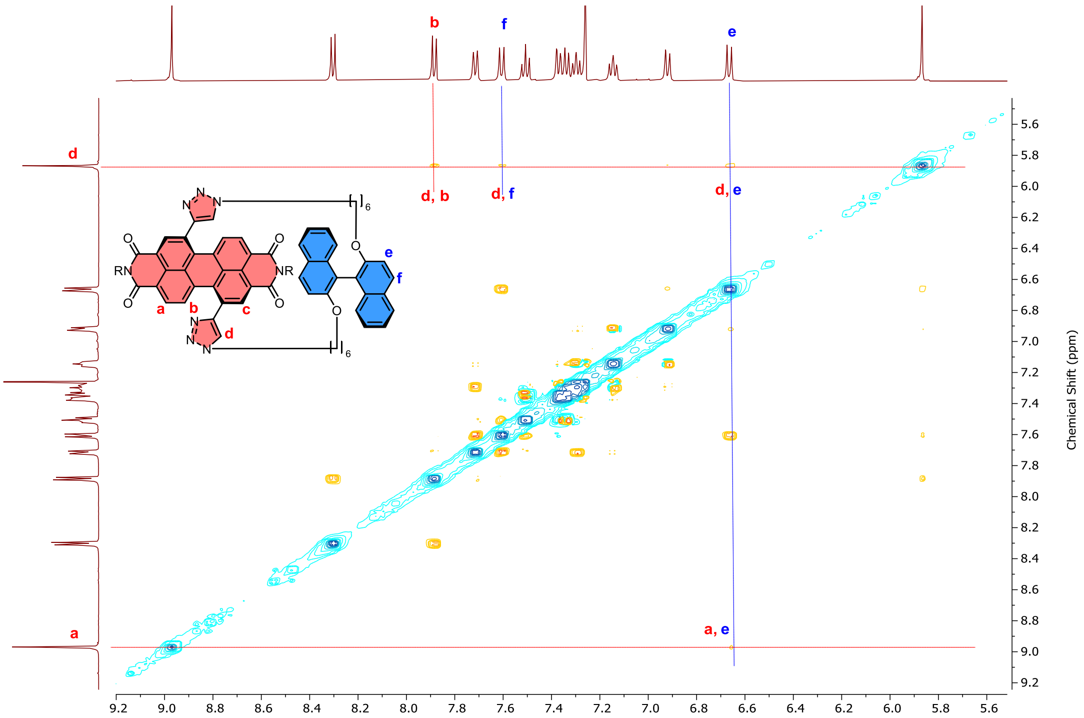

Fig. S1 ¹H–¹H NOESY NMR spectrum of ***PP*-2b** (500 MHz, $CDCl_3$), displaying nOe signals between protons $H_{d,f}$, $H_{d,e}$, and $H_{a,e}$, confirming the proximity of PDI and BINOL units in macrocycle **2b**.

3.2. ¹H NMR Spectra of the Stereoisomers of Macrocycle 2b

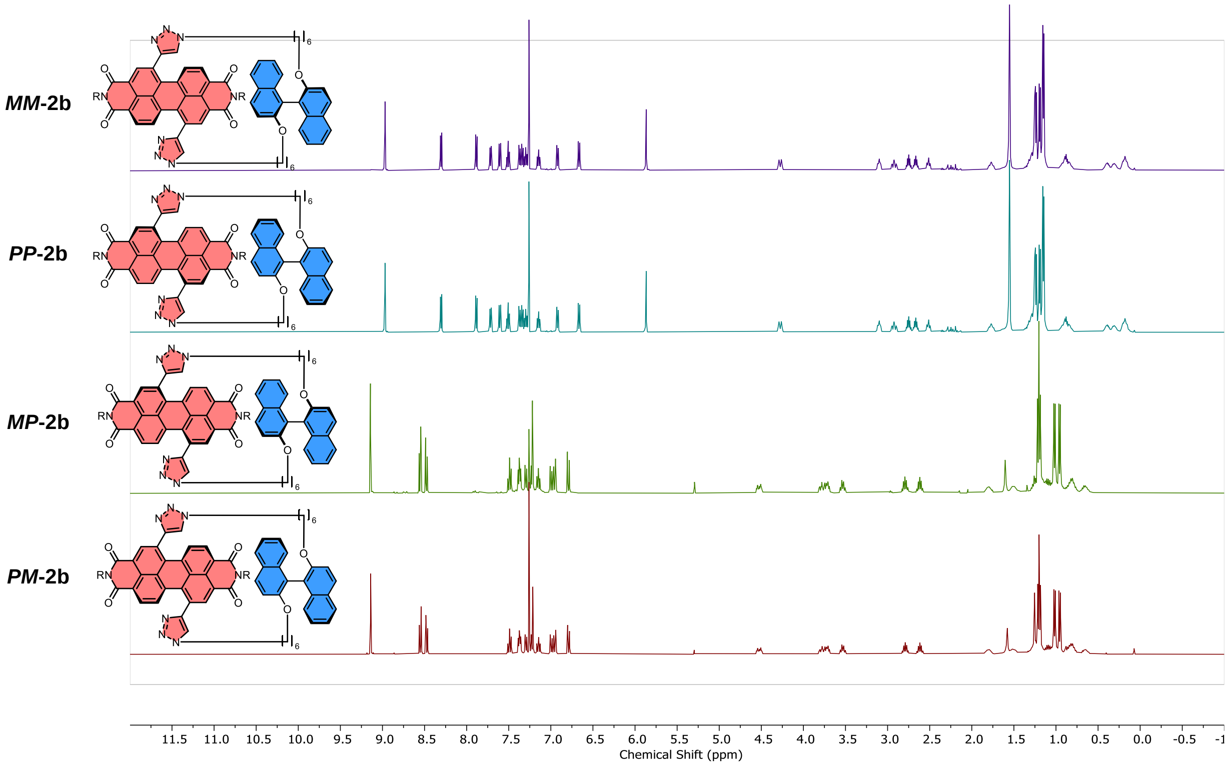

Fig. S2 ¹H NMR of **2b** (400 MHz, $CDCl_3$), displaying the identical spectra of macrocycles that have an enantiomeric relationship (e.g., ***MM*-2b** and ***PP*-2b**).

3.3. PDI Configurational Stability

Both diastereomers of macrocycle **2b** were subjected to heating at 140 °C for 12 hours in 1,1,2,2-tetrachloroethane, with ^{1}H NMR spectra were collected prior to and after heating. The identical ^{1}H NMR spectra indicate that PDI and BINOL atropisomers, and hence all diastereomers, are configurationally stable.

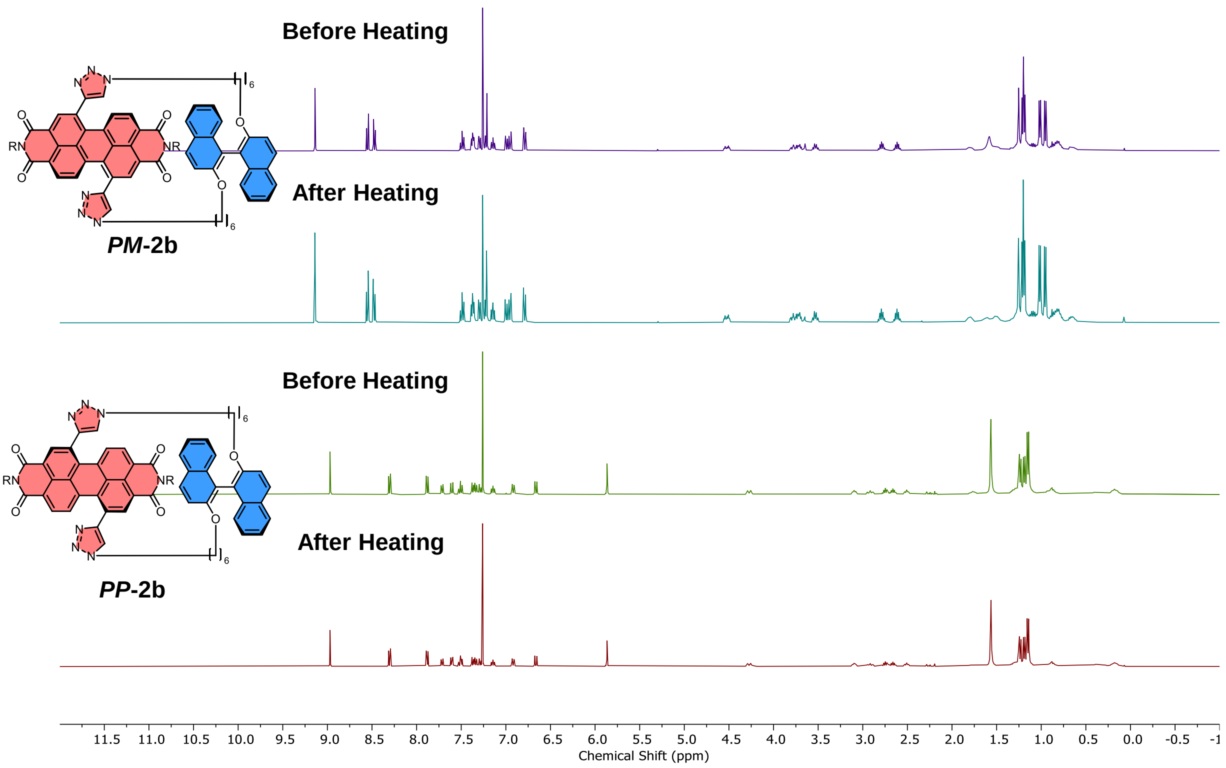

Fig. S3 ^{1}H NMR spectra of macrocycle **2b** (400 MHz, $CDCl_3$) before and after heating.

3.4. Diastereomeric Ratio Calculations by ¹H NMR Spectroscopy

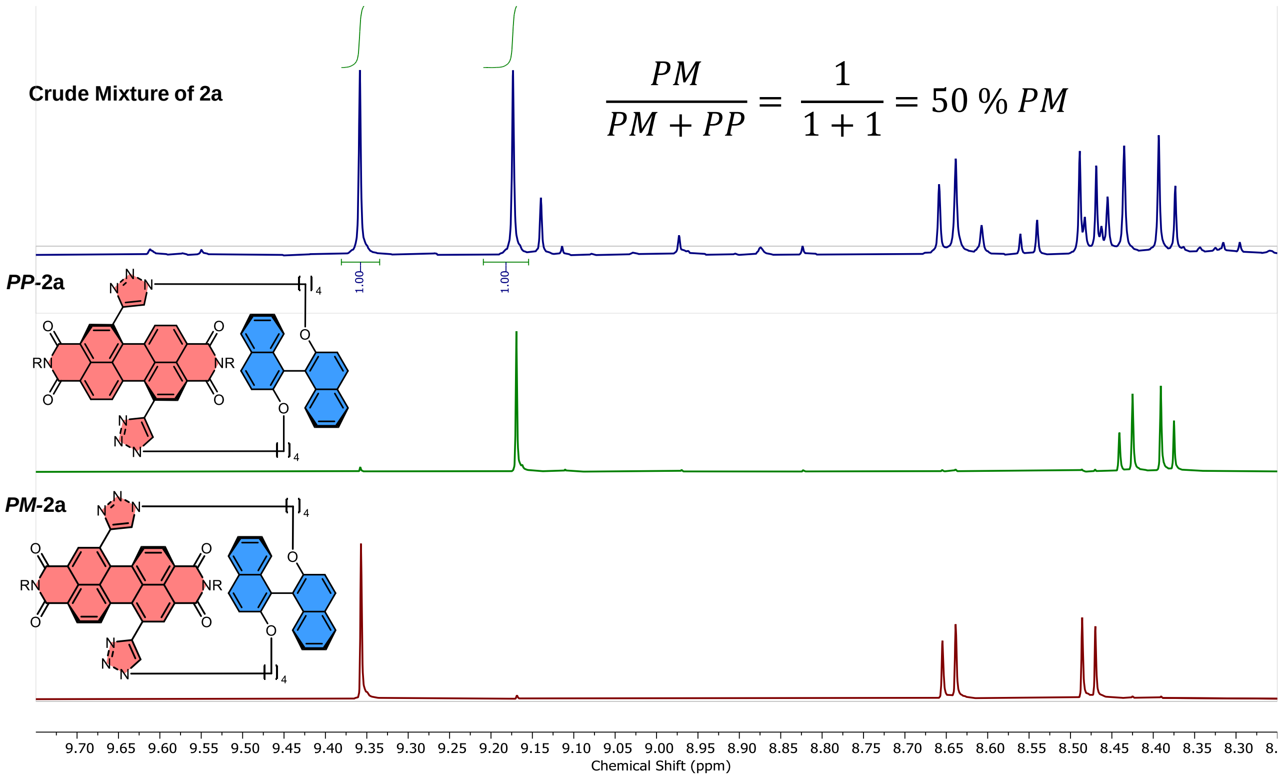

Fig. S4 ¹H NMR of crude reaction mixture of macrocycle **2a** (400 MHz, $CDCl_3$) and purified **2a**, displaying no significant stereoselectivity in the synthesis (i.e., ***PM:PP*** dr = 1:1 at room temperature). Dichloromethane was used as solvent in the synthesis.

$$\frac{PM}{PM+PP} = \frac{1+1.07}{1+1.07+0.26+0.29} = \textbf{\textit{79\% PM}}$$

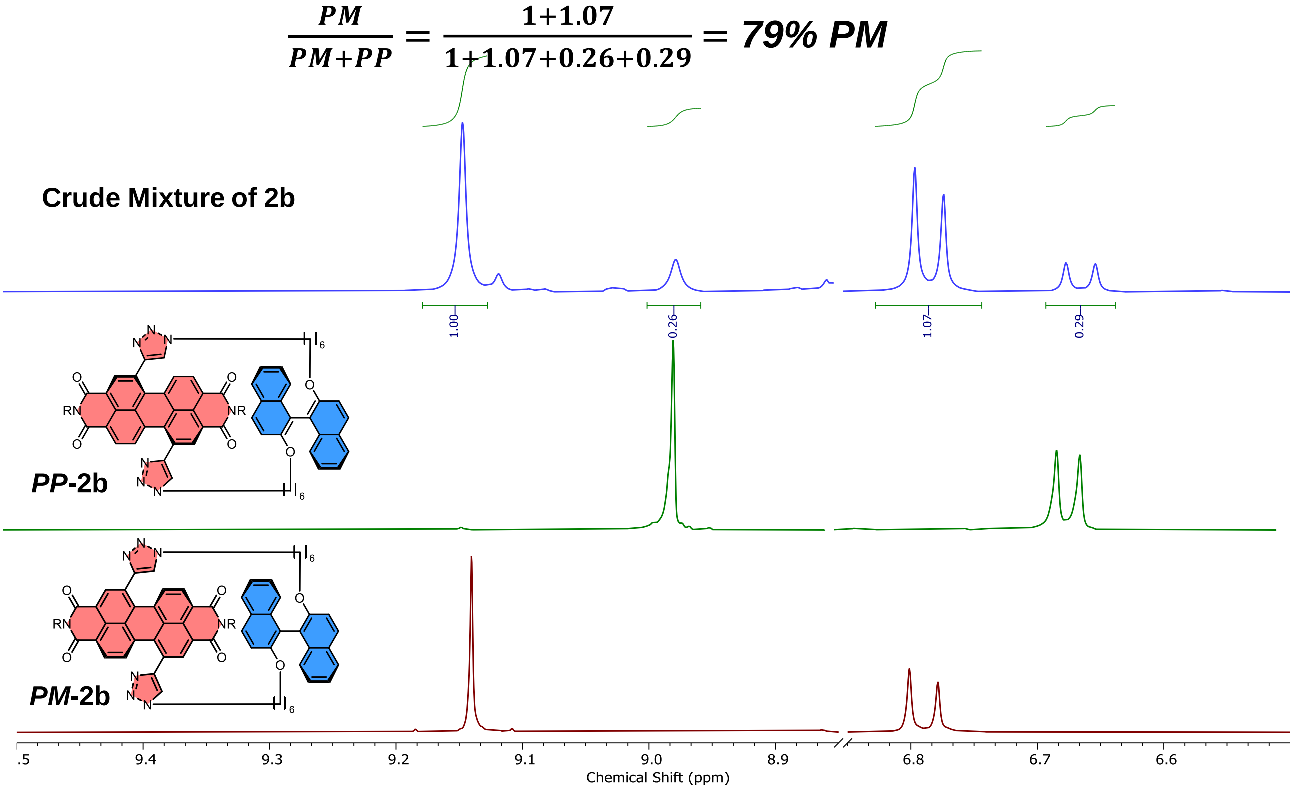

Fig. S5 ^{1}H NMR of crude reaction mixture of macrocycle **2b** (400 MHz, $CDCl_3$), and purified **2b**, displaying stereoselectivity in the synthesis (***PM:PP*** dr = 4:1 at room temperature). CH_2Cl_2 was used as solvent in the synthesis.

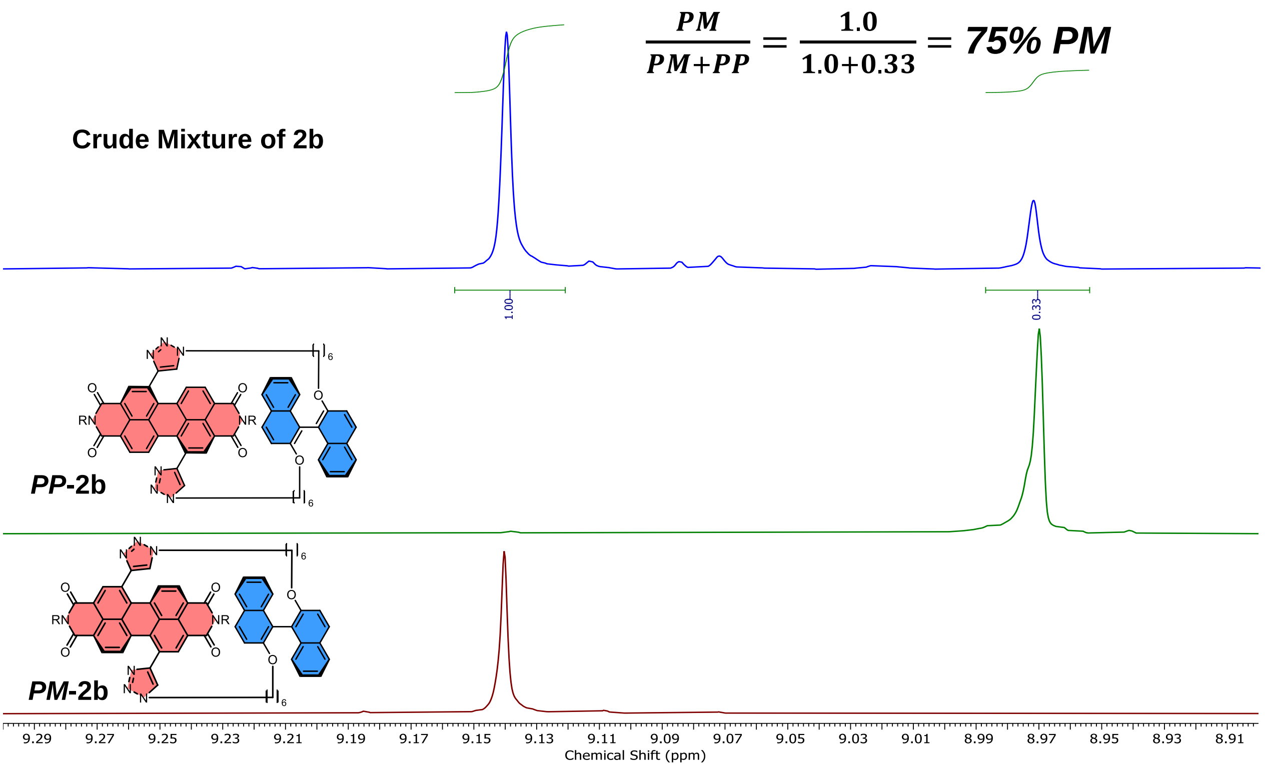

Fig. S6 ^{1}H NMR of crude reaction mixture of macrocycle **2b** (400 MHz, $CDCl_3$), and purified **2b**, displaying stereoselectivity in the synthesis (***PM:PP*** dr = 3:1 at room temperature). A 1:1 mixture of toluene:CH_2Cl_2 (v/v) was used as solvent in the synthesis.

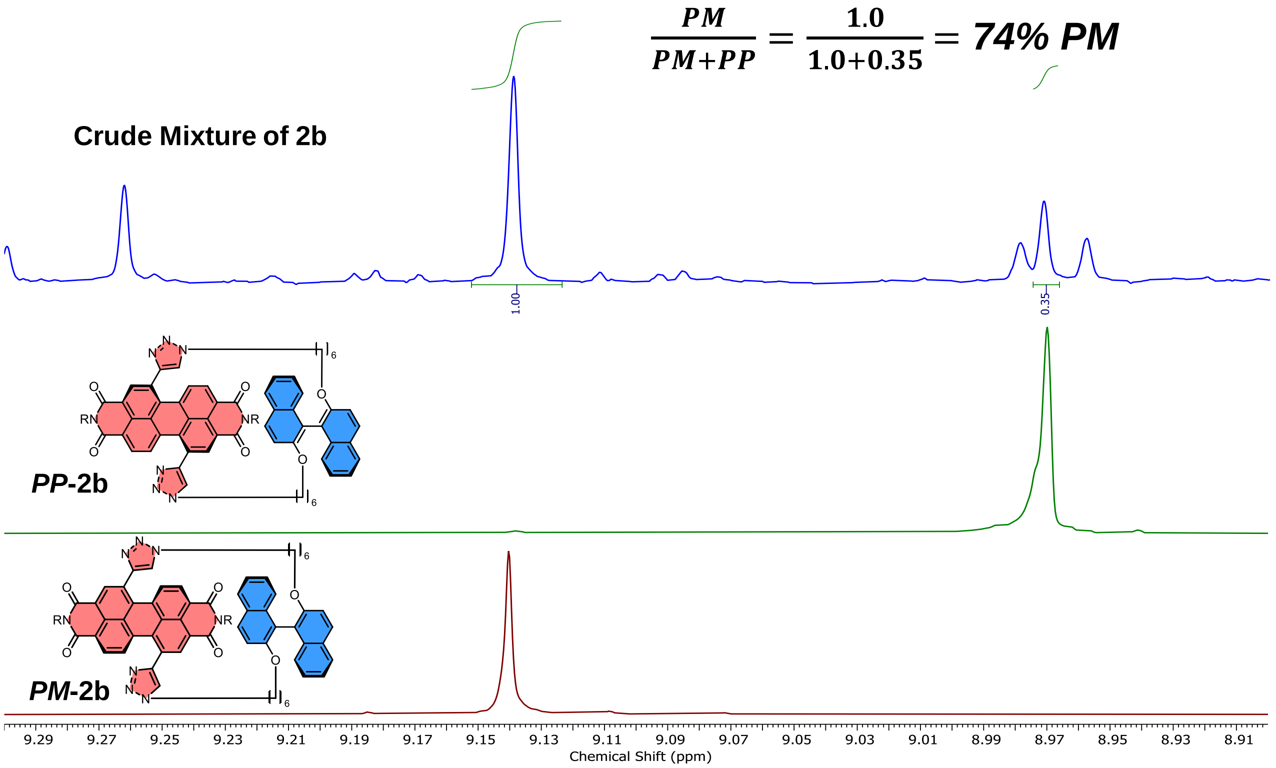

Fig. S7 ^{1}H NMR of crude reaction mixture of macrocycle **2b** (400 MHz, $CDCl_3$), and purified **2b**, displaying stereoselectivity in the synthesis (***PM:PP*** dr = 3:1 at room temperature). *N*,*N*-Dimethylformamide (DMF) was used as solvent in the synthesis.

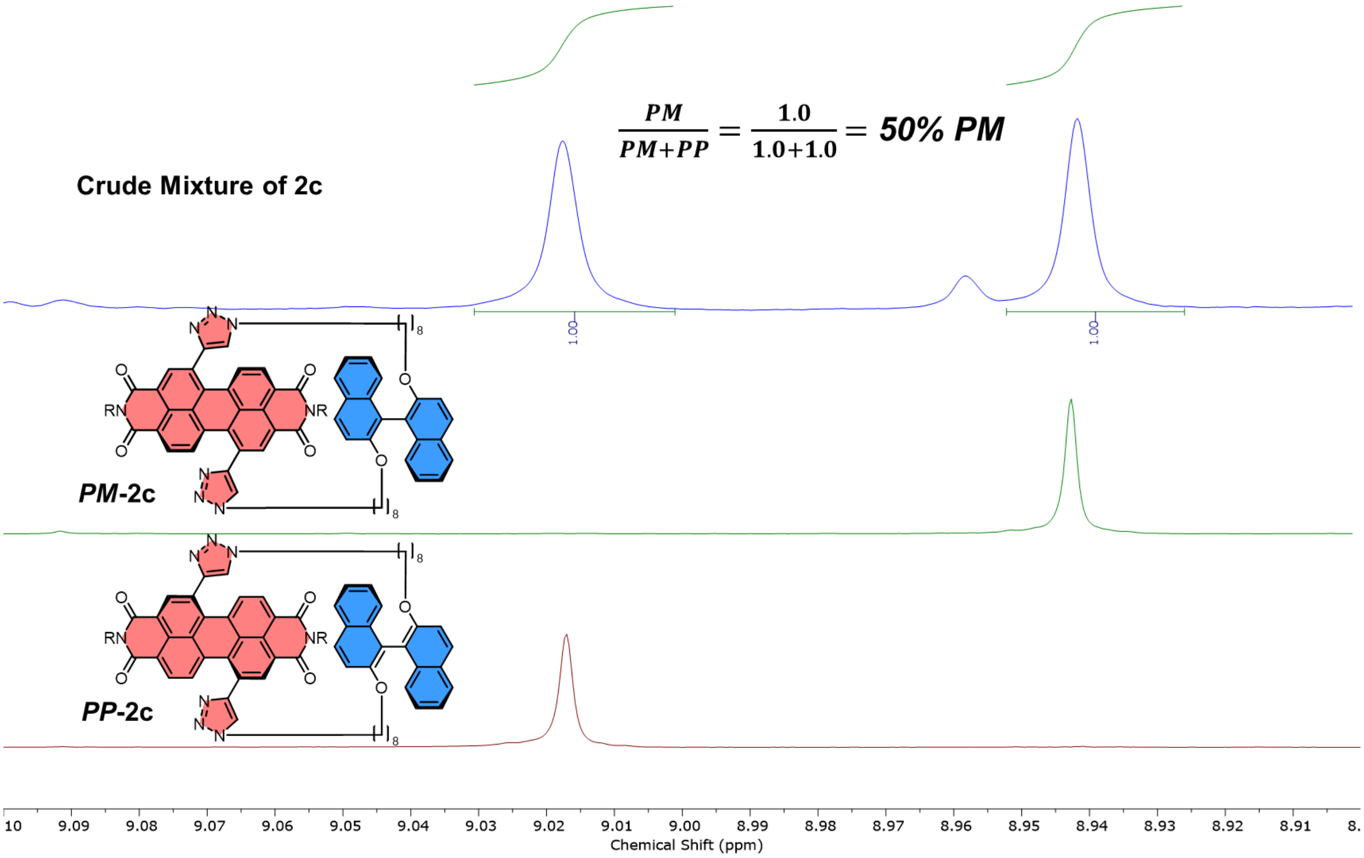

Fig. S8 ^{1}H NMR of crude reaction mixture of macrocycle **2c** (400 MHz, $CDCl_3$) and purified **2c**, displaying no significant stereoselectivity in the synthesis (i.e., ***PM:PP*** dr = 1:1 at room temperature). Dichloromethane was used as solvent in the synthesis.

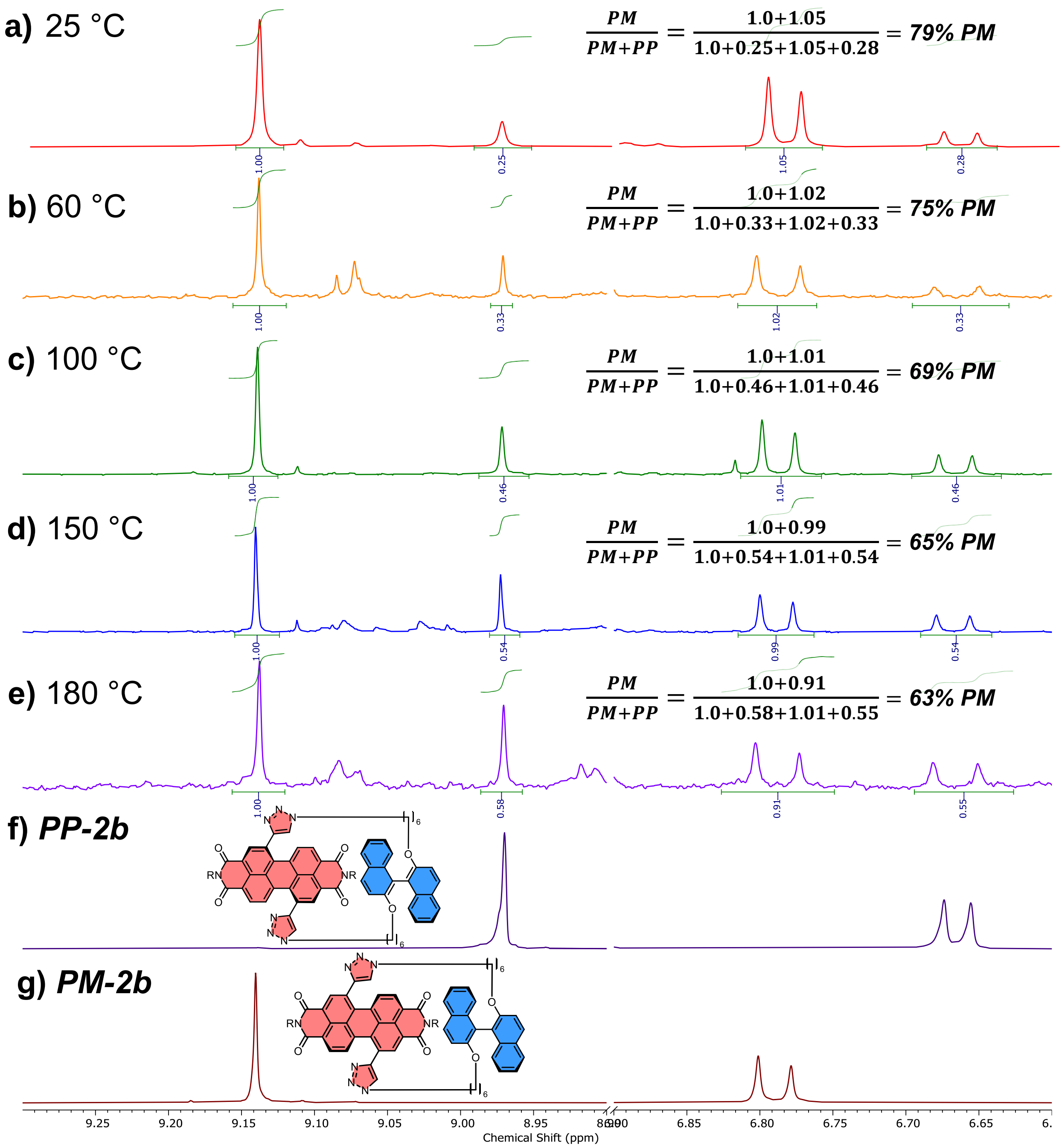

Fig. S9 ^{1}H NMR of: **a)-e)** crude reaction mixture of macrocycle **2b** (400 MHz, $CDCl_3$), reacted at stated temperatures with 1,2-dichlorobenzene as solvent; and **f)-g)** purified **2b**. The ***PM:PP*** diastereomeric ratio are: **a)** 79:21 at room temperature (25 °C), **b)** 75:25 at 60 °C, **c)** 69:31 at 100 °C, **d)** 65:35 at 150 °C, and **e)** 63:37 at 180 °C. These values are used to calculate the difference in standard Gibbs energies between the ***PP*-2b** and ***PM*-2b** transition states ($\Delta\Delta G^{\ddagger}$) following the Curtin-Hammett principle below (Fig. S10).

3.5. Calculation of $\Delta\Delta G^{\ddagger}$ following the Curtin-Hammett principle

The Curtin-Hammett principle[12-14] states that for a rapidly interconverting reactive intermediates or reactants, which respectively irreversibly forms a different product, the product ratio will depend only on the difference in energy between the transition states ($\Delta\Delta G^{\ddagger}$). This can be written as equation (1).

$$Product\ ratio = e^{-\Delta\Delta G\ddagger/\mathrm{RT}} \quad (1)$$

Where, in context:

Product ratio = [***PM*-2b**]/[***PP*-2b**]

$\Delta\Delta G^{\ddagger}$ = free energy difference between transition states of the two products (i.e., ***PM*-2b** and ***PP*-2b**)

R = Ideal gas constant

T = Temperature

Rearranging (1) gives the following equation:

$$\ln(product\ ratio) = \frac{-\Delta\Delta G\ddagger}{RT} \quad (2)$$

Using the product ratio calculated from ^{1}H NMR spectra above (Fig. S9), ln(product ratio) was plotted against 1/T to give a slope of $-\Delta\Delta G^{\ddagger}/R$ (754 ± 53 J mol^{-1}, Fig. S10). This was used to calculate $\Delta\Delta G^{\ddagger}$ (−6.3 ± 0.4 kJ mol^{-1}).

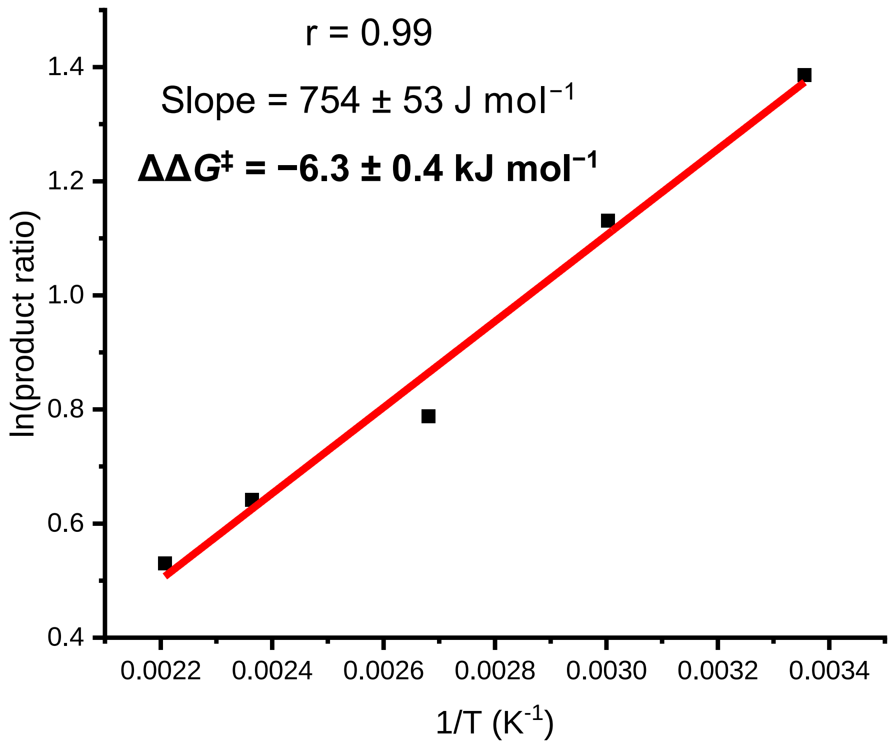

Fig. S10 Curtin-Hammett plot of ln(product ratio) against 1/T.

3.6. Comparisons of ^{1}H NMR Spectra of Macrocyclic and Acyclic Systems

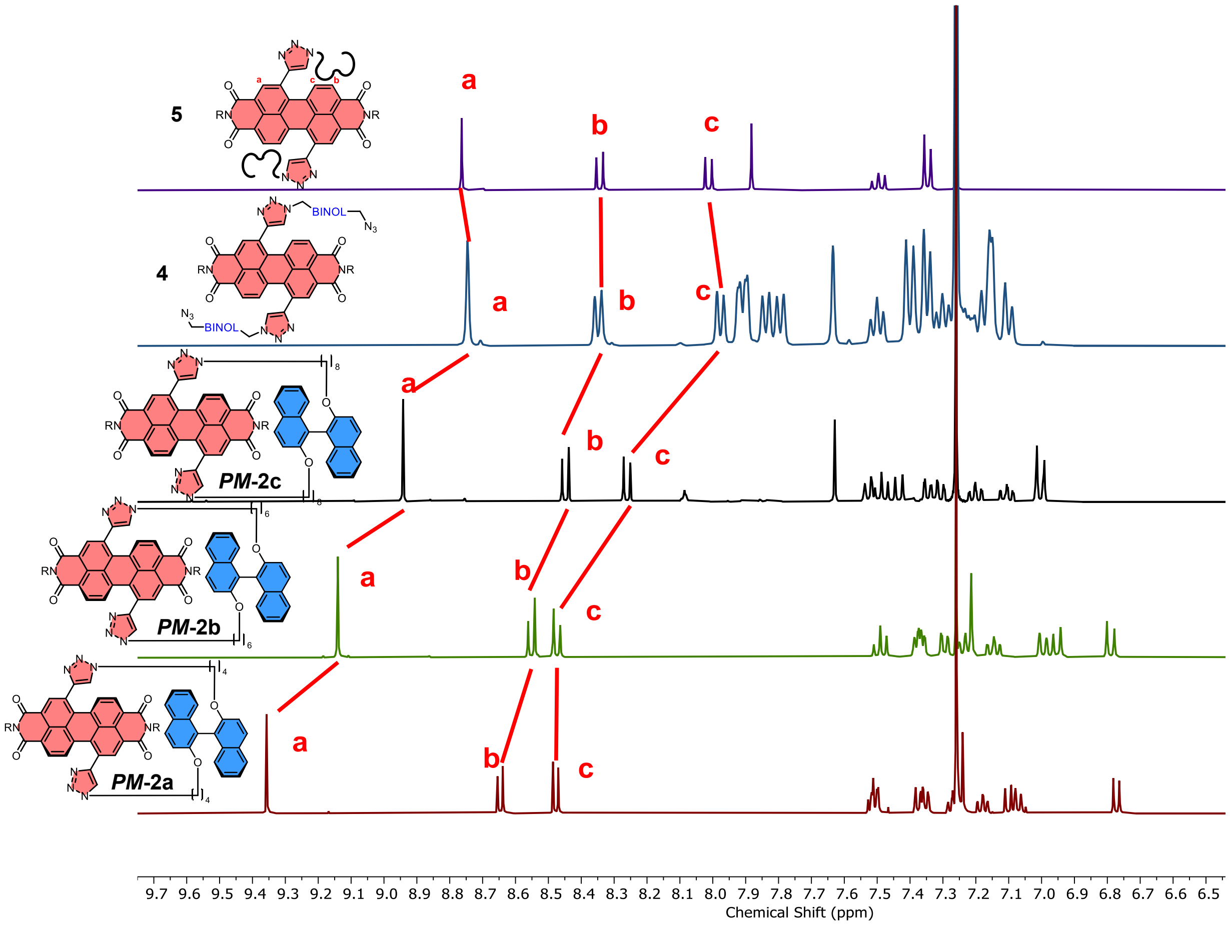

Fig. S11 Stacked ^{1}H NMR spectra of acyclic bis-triazole PDI **5**, acyclic bis-BINOL PDI **4**, BINOL–PDI macrocycles **2a** and **2b** ($CDCl_3$, 298 K, 400 MHz).

3.7 Comparisons of ¹H NMR Spectra of Homo and Heterochiral Macrocycles 2b against BINOL acyclic starting material 12b

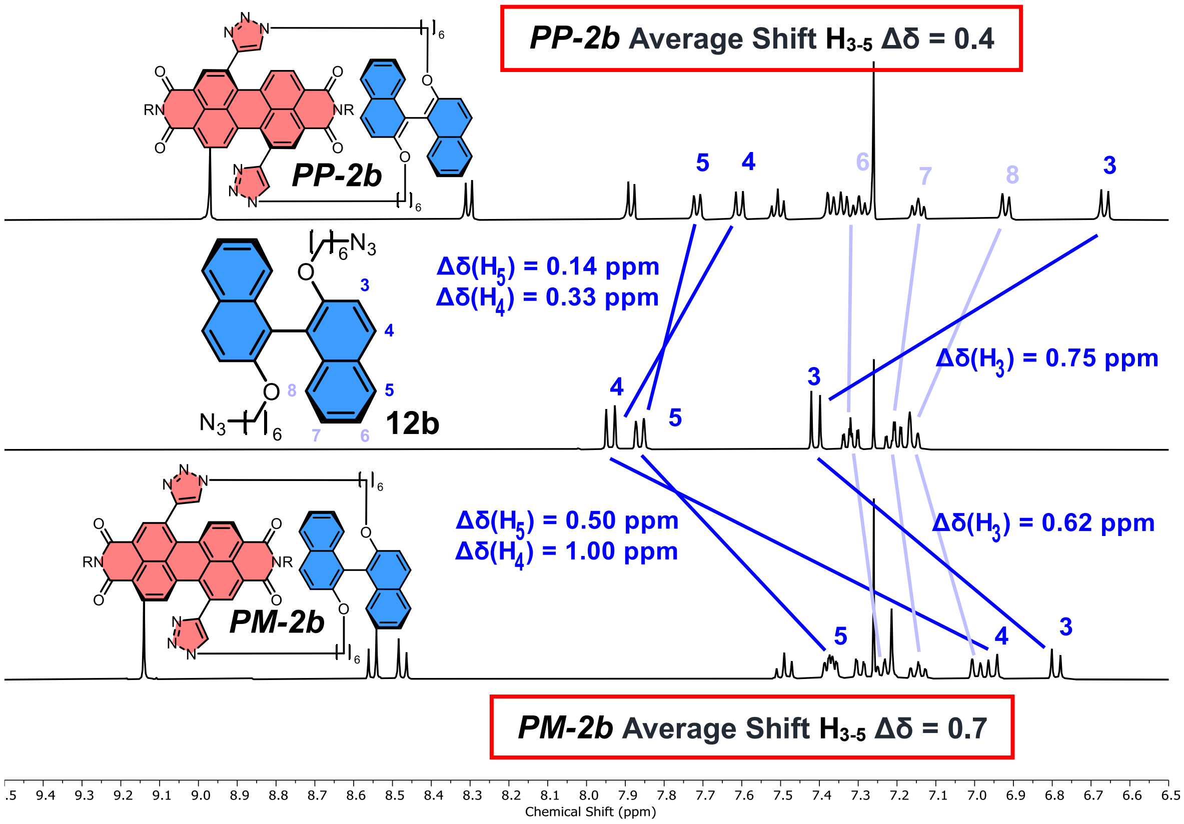

Fig. S12 Stacked ¹H NMR spectra ($CDCl_3$, 298 K, 400 MHz) of acyclic BINOL **12b** against both homo and heterochiral BINOL–PDI macrocycles **2b**, displaying a higher average upfield shift from $H_{3\text{-}5}$ in ***PM*-2b** (0.7 vs 0.4). This suggests a stronger interaction between the BINOL and PDI units in ***PM*-2b** compared to ***PP*-2b**, which is further supported by shorter π–π and CH–π distances and more numerous CH–π interactions seen in calculated structures in Section 8.

3.8 Estimation of $\Delta G_{int}^{\ddagger}$ (upper bound)

The interconversion of PDI atropisomers were found, by Würthner and co-workers,[15] to be dependent on the apparent overlap parameter $\sum r^*$ of the bay substituent (Y) according to equation (1) below:

$$\Delta G^{\ddagger}\ (180\ \mathrm{K}) = 29.0 \sum r^* - 41.8 \qquad (1)$$

Given the C–H bond length and van der Waals radius of hydrogen, $\sum r^*$ can be calculated from equation (2):

$$\sum r^* = 2r_{vdW}(Y) + 2.40\ \text{Å} - 2\sqrt{(2.312\ \text{Å} - 0.5a)^2 + (-0.953\ \text{Å} + 0.866a)^2} \quad (2)$$

Where:

$r_{vdW}(Y) = van\ der\ Waals\ radius\ of\ Y$ (bay substituent)

$a = Bond\ length\ of\ C-Y$

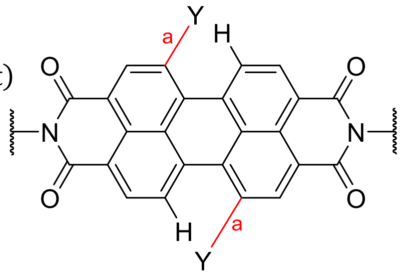

For our diethynyl PDI starting material **8**,

Substituting $r_{vdW}(ethynyl) = 1.78$,[15] and $a = 1.53$,[15] gives:

$$\sum r^* = 2.78\ \text{Å}$$

Substituting into equation (1) gives:

$$\Delta G^{\ddagger}\ (180\ \mathrm{K}) = 38.8\ \mathrm{kJ/mol}$$

This gives an upper bound of $\Delta G^{\ddagger} < 39$ kJ mol^{-1}, and hence rate $k > 6 \times 10^5$ s^{-1}.

For our acyclic bis-triazole PDI control compounds (**3-5**), and the singly 'clicked' CuAAC intermediate (Fig. 5), we assume that the triazole heterocycle must twist away from the plane of the PDI as the PDI interconverts, giving an estimated value $r_{vdW}(triazole) = 1.77$.[15]

Substituting this value into equation (2) and then (1) gives:

$$\sum r^* = 2.76\ \text{Å} \text{ and } \Delta G^{\ddagger}\ (180\ \mathrm{K}) = 38.2\ \mathrm{kJ/mol}$$

This gives an upper bound of $\Delta G^{\ddagger} < 39$ kJ mol^{-1}, and hence rate $k > 6 \times 10^5$ s^{-1}.

The authors also state that for room-temperature stable atropisomers of PDIs, $\Delta G^{\ddagger} > 106$ kJ mol^{-1}, apparent overlap $\sum r^*$ must be greater than 5.1 Å.[15] This is clearly not the case for acyclic PDI compounds **3-5**.

Indeed, ^{1}H NMR spectroscopy of acyclic variants **3-5** (and **6-8**) all show that the diastereotopic methyl protons exhibit only one doublet, and thus are in fast exchange at 25 °C (see below Fig. S13). Therefore, the rate of PDI atropisomer interconversion is faster than the chemical shift timescale of the ^{1}H NMR experiment. However, the ^{1}H NMR spectra of chirally locked PDI macrocycles **1-2** all show the diastereotopic methyl protons are split into four sets of doublets, and thus are in slow exchange at 25 °C (i.e., a high PDI interconversion barrier at 25 °C). Therefore, the rate of PDI atropisomer interconversion is much slower than the chemical shift timescale of the ^{1}H NMR experiment.[16]

3

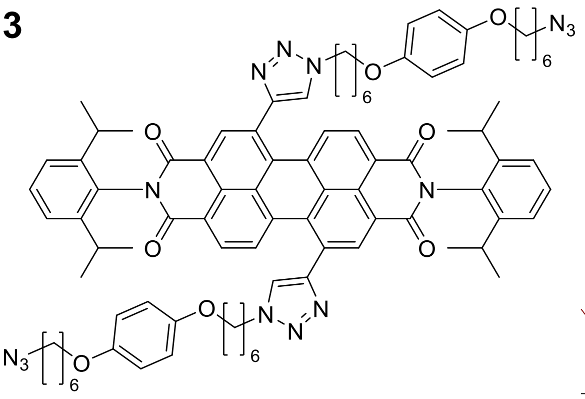

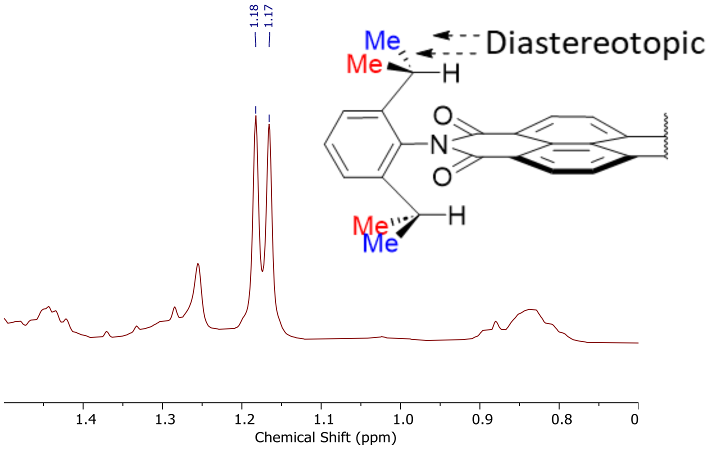
1.18
1.17
Me
Me
Diastereotopic
H
O
N
O
Me
Me
H
1.4
1.3
1.2
1.1
1.0
0.9
0.8
0
Chemical Shift (ppm)

4

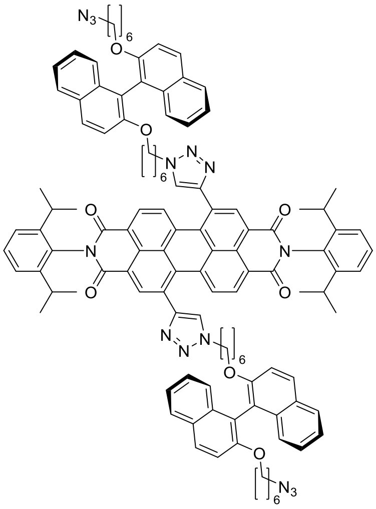

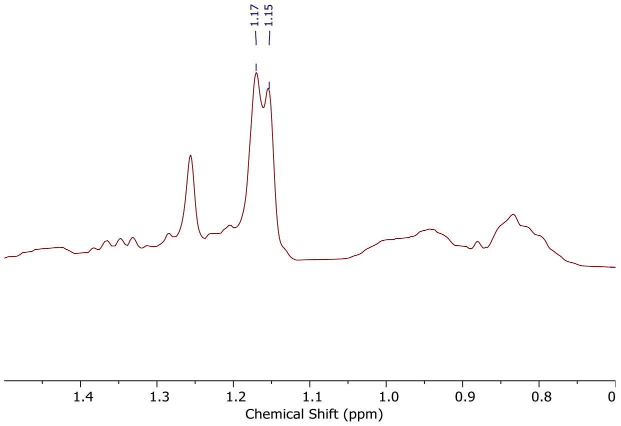
1.17
1.15
1.4
1.3
1.2
1.1
1.0
0.9
0.8
0
Chemical Shift (ppm)

5

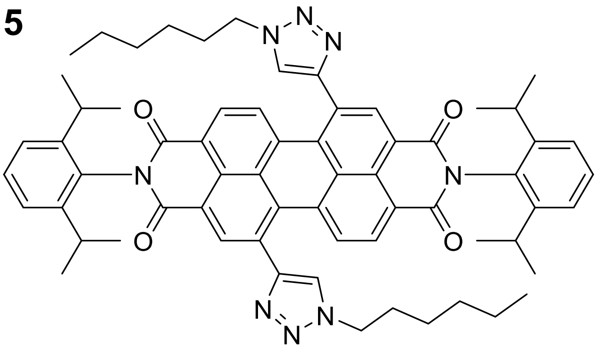

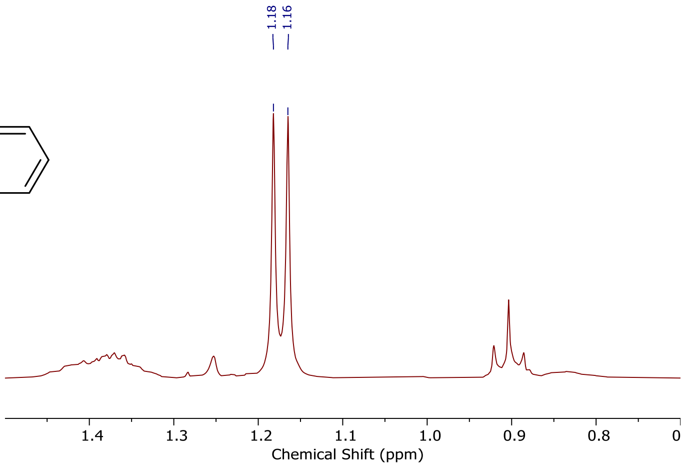
1.18
1.16
1.4
1.3
1.2
1.1
1.0
0.9
0.8
0
Chemical Shift (ppm)

6

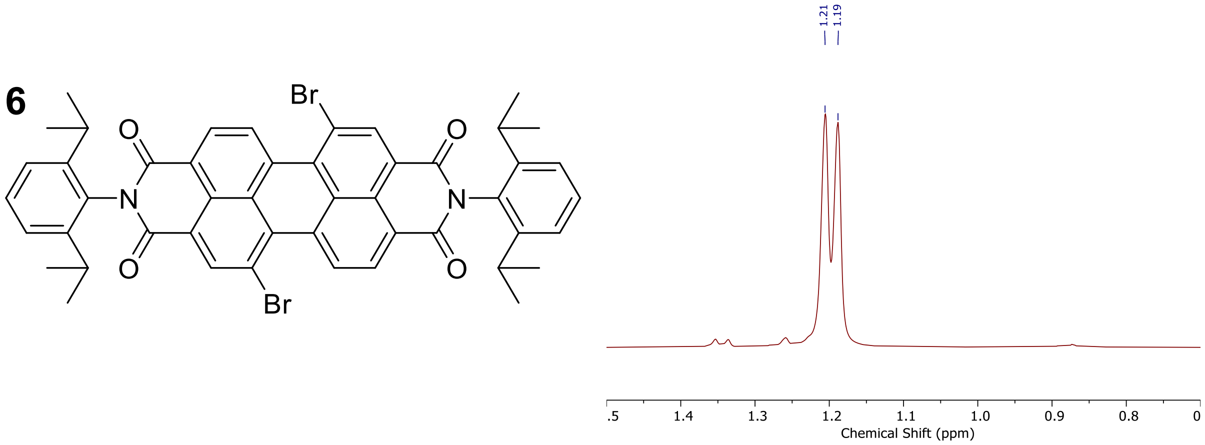

7

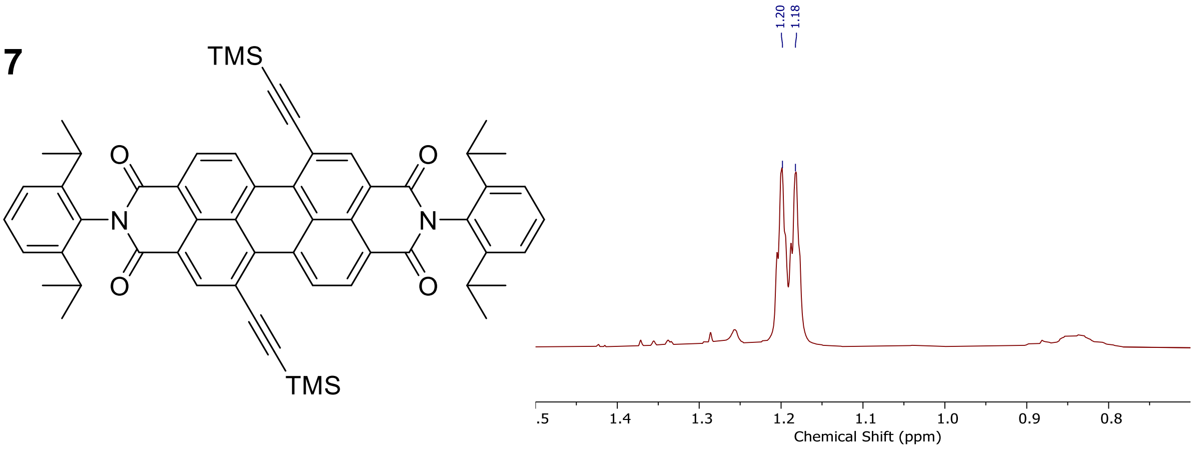

8

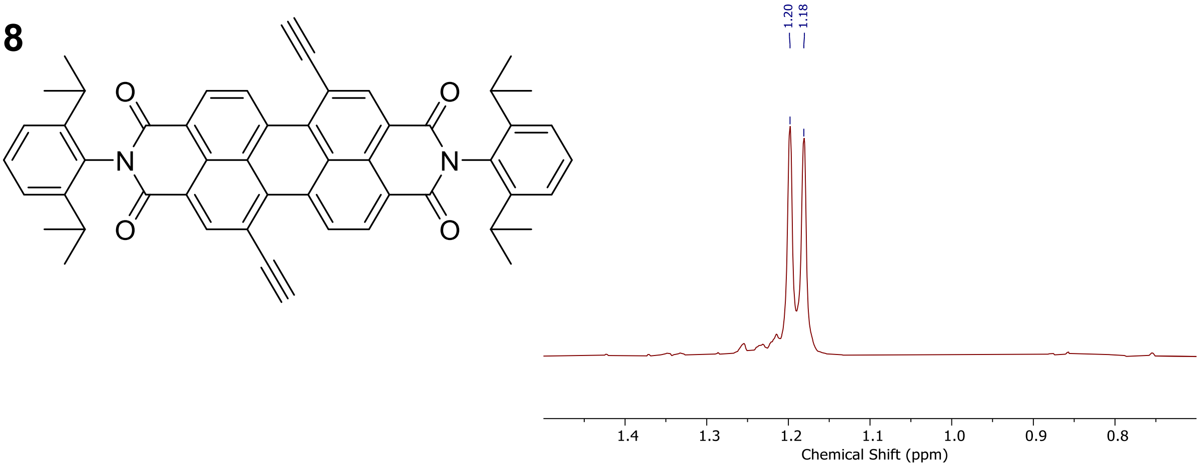

Fig. S13 ^{1}H NMR spectra of acyclic PDIs **3-8**, displaying a single set of doublets for methyl groups, displaying no diastereotopic splitting as PDI atropoisomers are in fast exchange ($CDCl_3$, 298 K, 400 MHz).

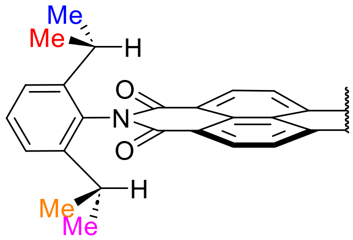
Me
Me
H
O
N
O
H
Me
Me

1a

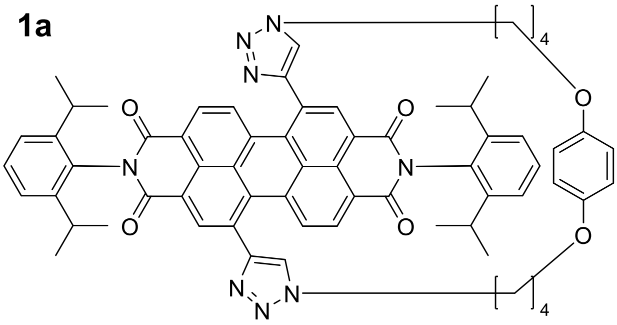
N
N
N
O
N
O
4
4

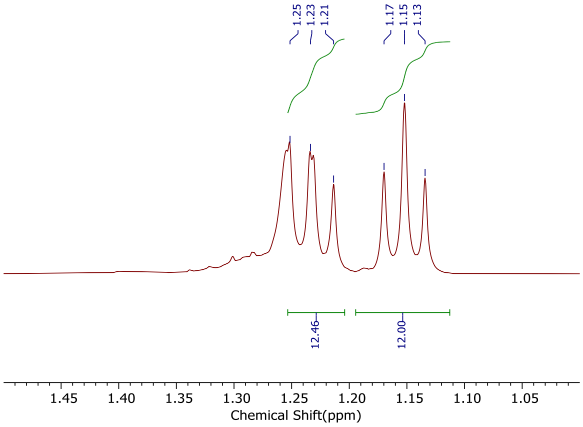
1.25
1.23
1.21
1.17
1.15
1.13
12.46
12.00
1.45 1.40 1.35 1.30 1.25 1.20 1.15 1.10 1.05
Chemical Shift(ppm)

1b

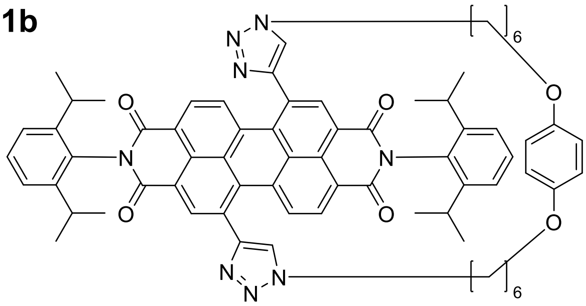
N
N
N
O
N
O
6
6

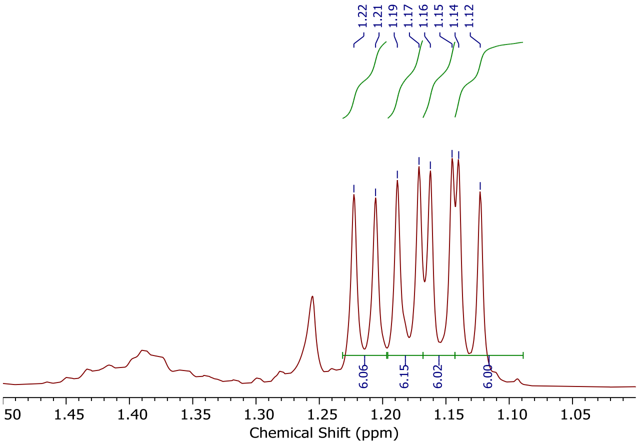
1.22
1.21
1.19
1.17
1.16
1.15
1.14
1.12
6.06
6.15
6.02
6.00
50 1.45 1.40 1.35 1.30 1.25 1.20 1.15 1.10 1.05
Chemical Shift (ppm)

PM-2a

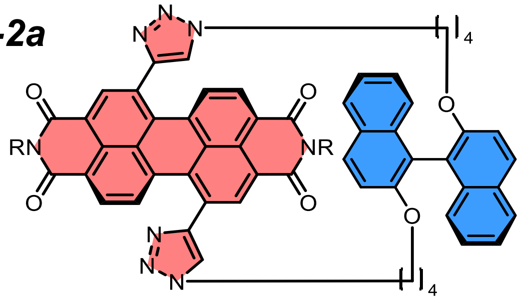
N
N
N
O
RN
NR
O
4
4

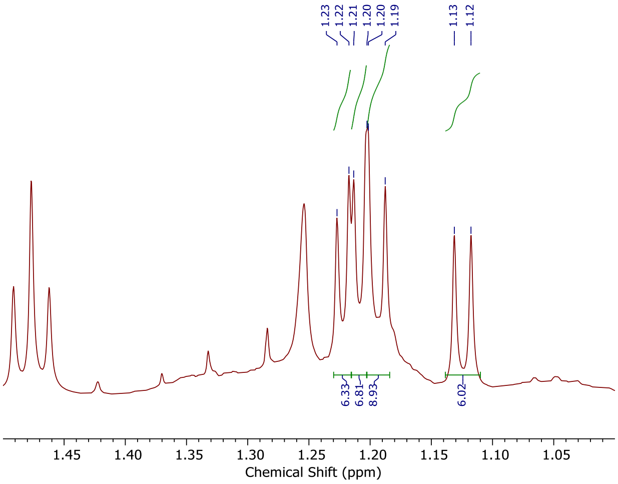
1.23
1.22
1.21
1.20
1.20
1.19
1.13
1.12
6.33
6.81
8.93
6.02
1.45 1.40 1.35 1.30 1.25 1.20 1.15 1.10 1.05
Chemical Shift (ppm)

PP-2a

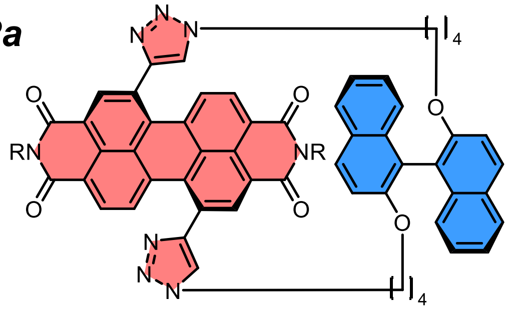

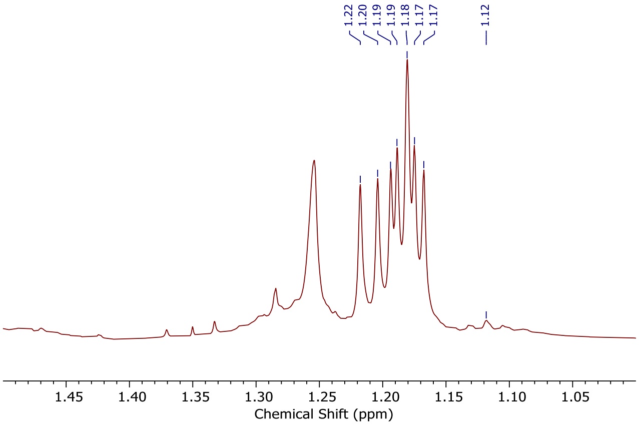

***PM*-2b**

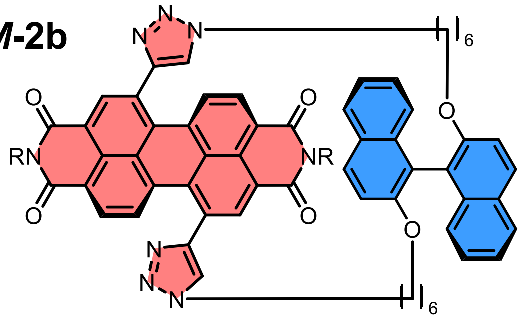

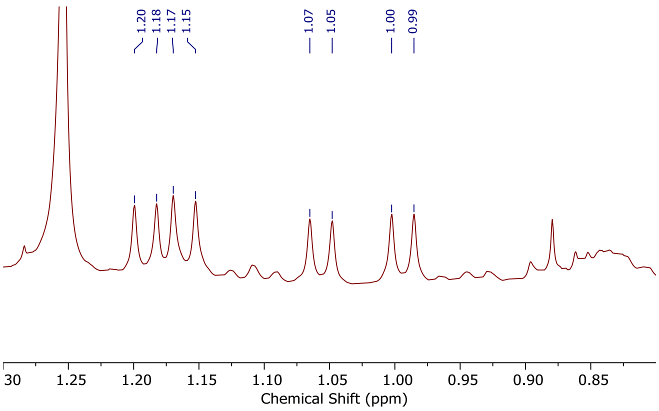

***PP*-2b**

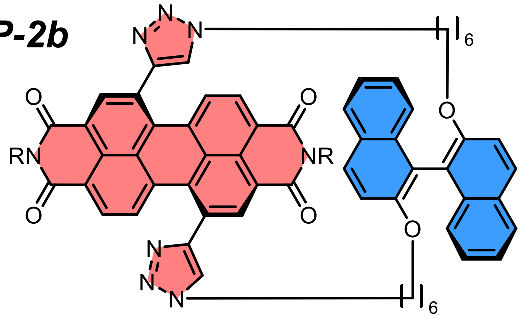

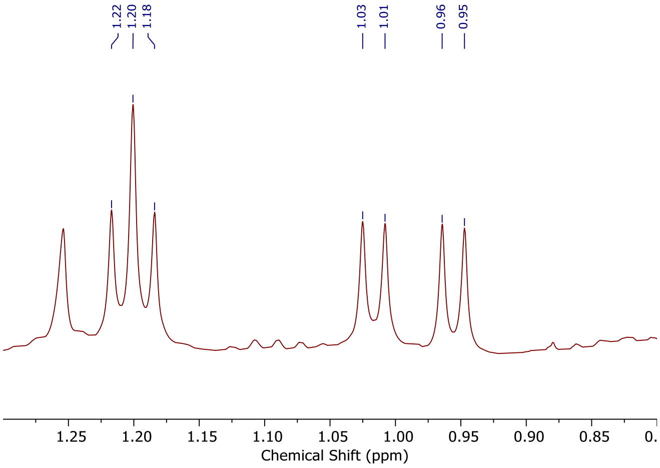

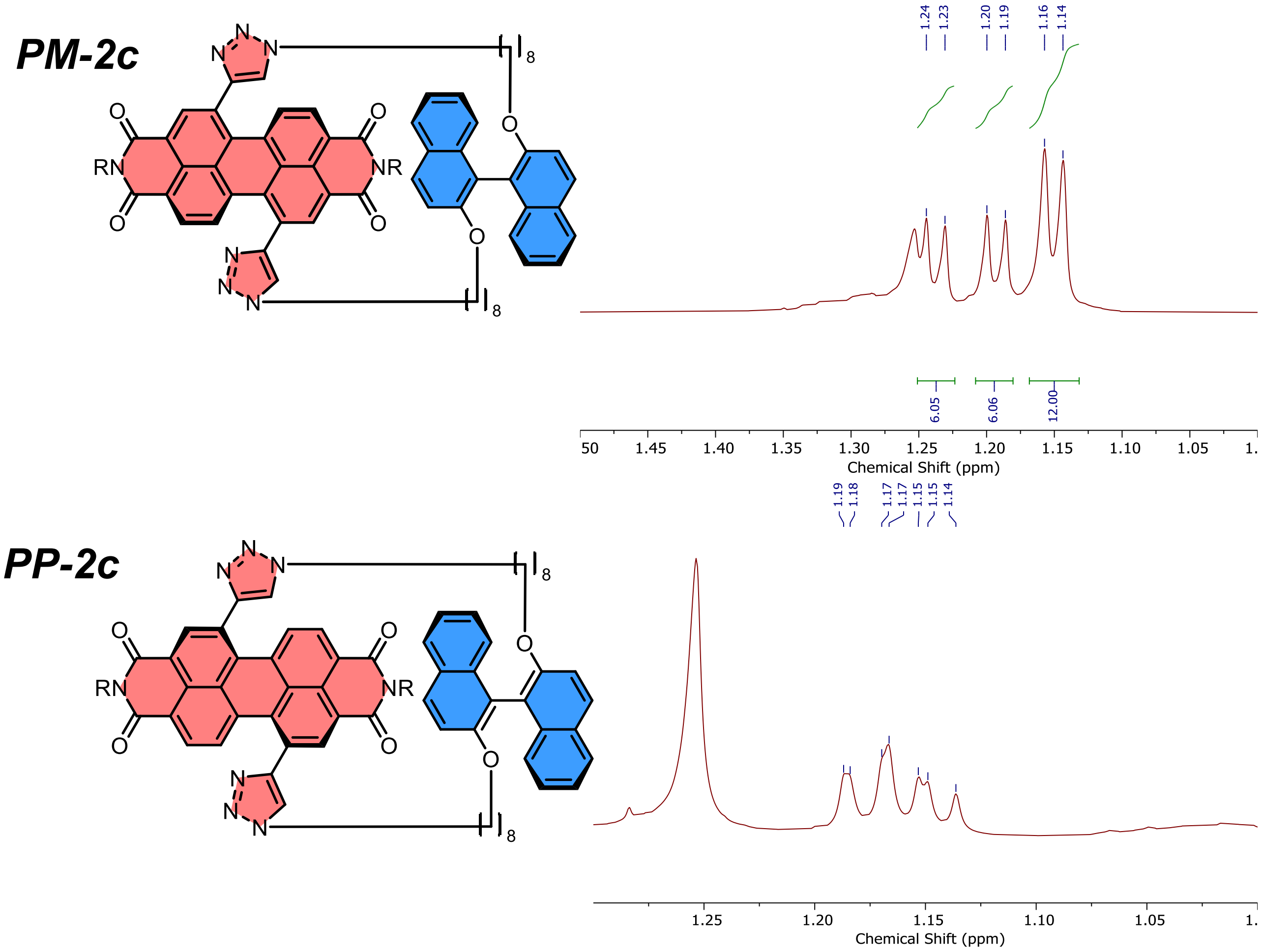

Fig. S14 ^{1}H NMR spectra of PDI macrocycles **1-2**, displaying four sets of doublets for methyl groups due to diastereotopic splitting arising from chiral locking of the PDI ($CDCl_3$, 298 K, 400 MHz).

4. Circular Dichroism Studies

Circular dichroism (CD) spectra were recorded on a Jasco J-1500 CD spectrophotometer with a wavelength accuracy ± 0.2 nm (250 to 500 nm), ± 0.5 nm (500 to 800 nm) and a CD root mean square noise < 0.007 mdeg (500 nm). A quartz cuvette with 1 mm path length was used unless stated otherwise.

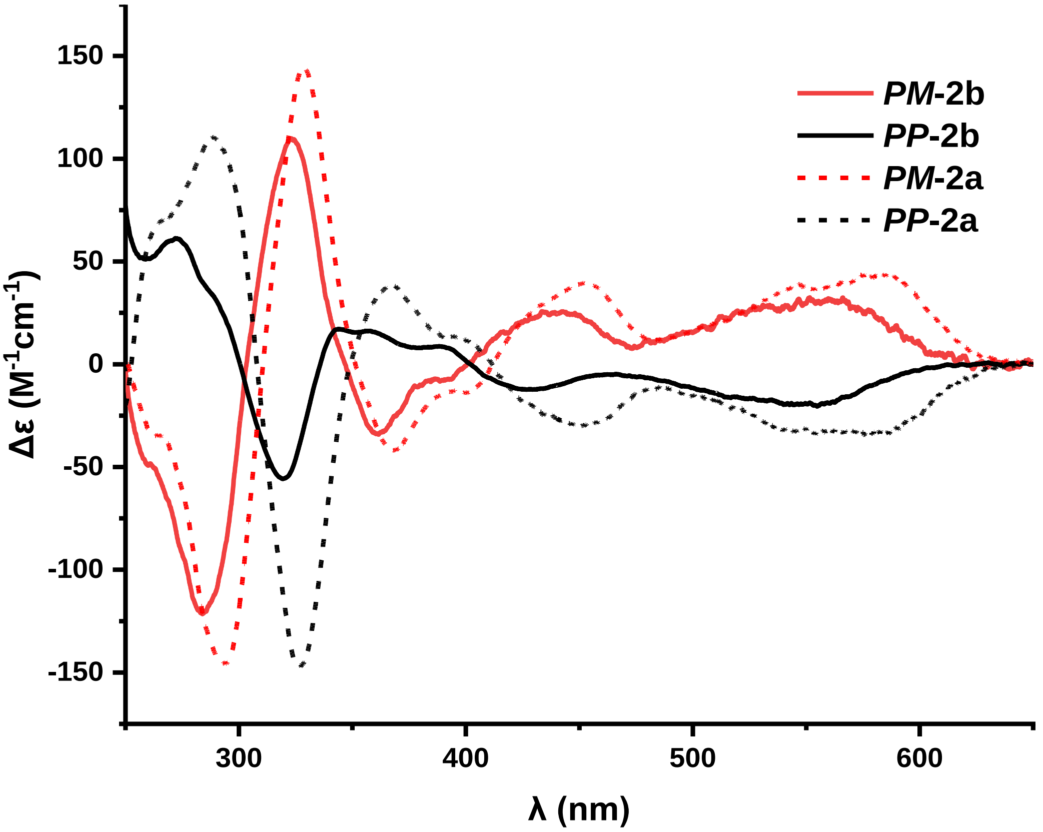

Fig. S15 CD spectra for ***PP*** and ***PM*** diastereomers of macrocycles **2b** and **2a** in $CHCl_3$ at a concentration of 26 μM.

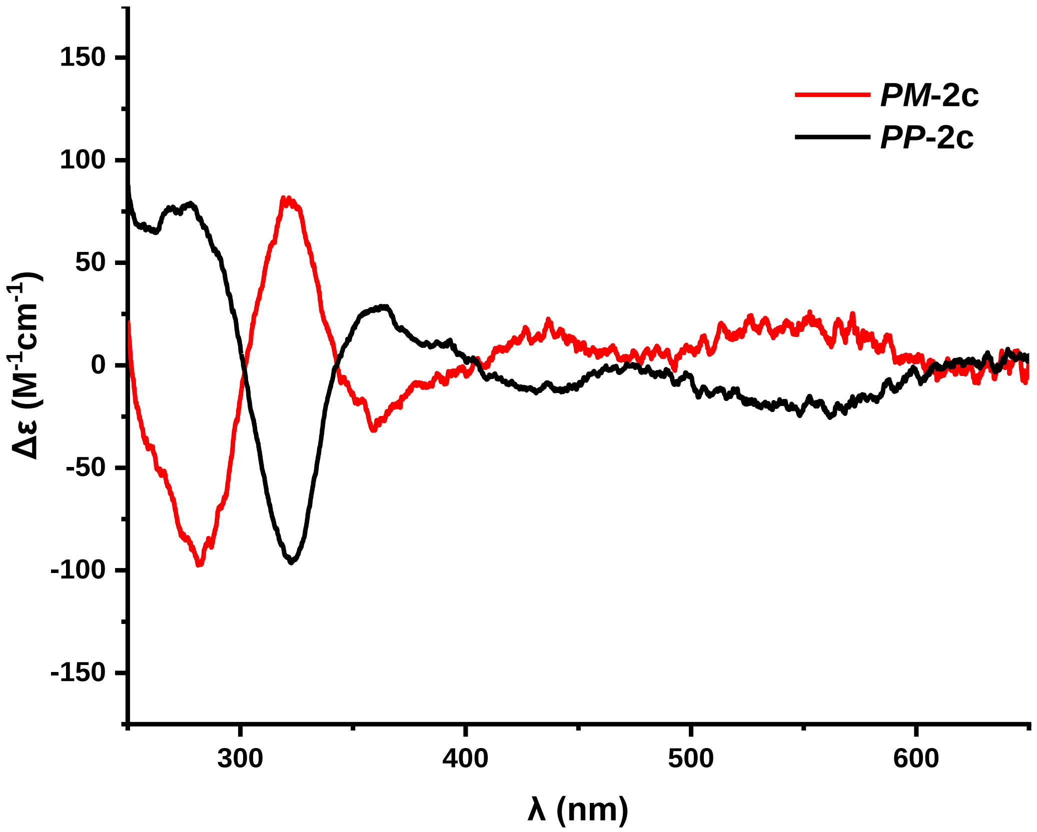

Fig. S16 CD spectra for ***PP*** and ***PM*** diastereomers of macrocycle **2c** in $CHCl_3$ at a concentration of 26 μM.

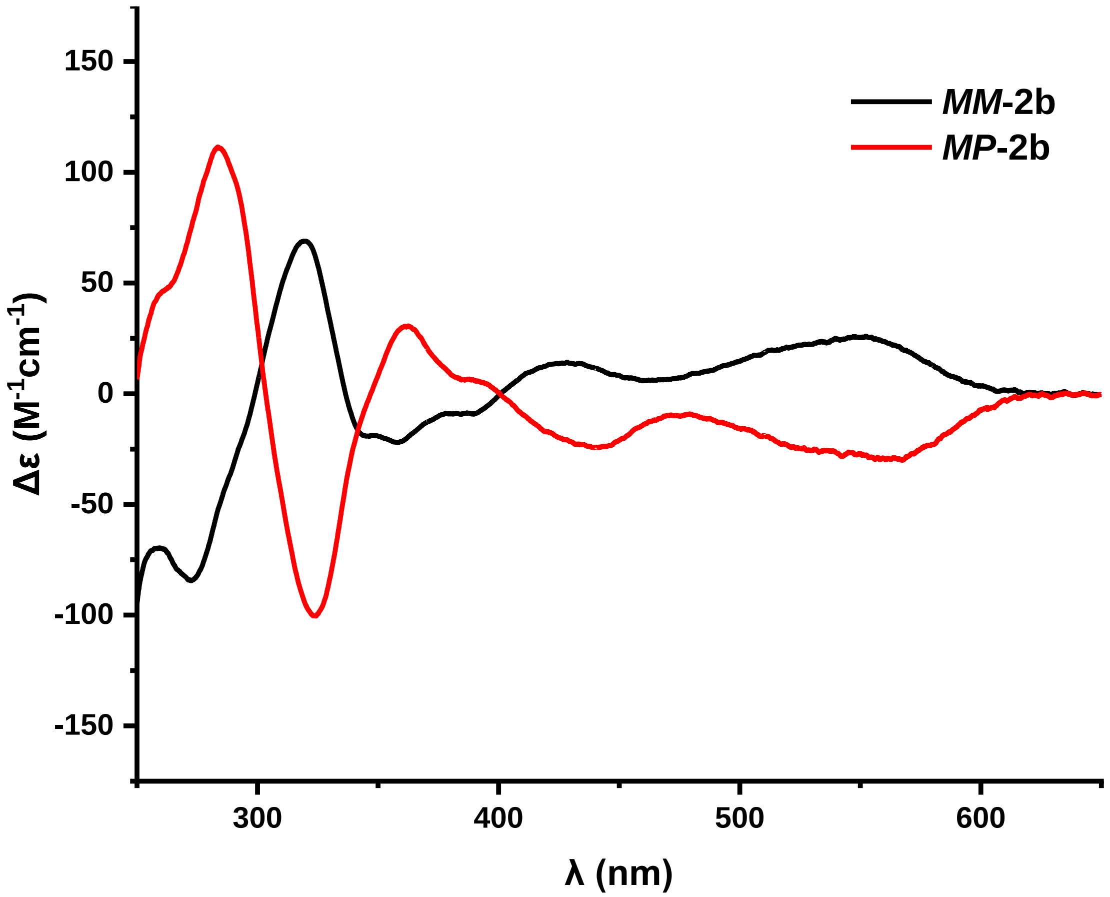

Fig. S17 CD spectra for ***MM*** and ***MP*** diastereomers of macrocycle **2b** in $CHCl_3$ at a concentration of 26 μM.

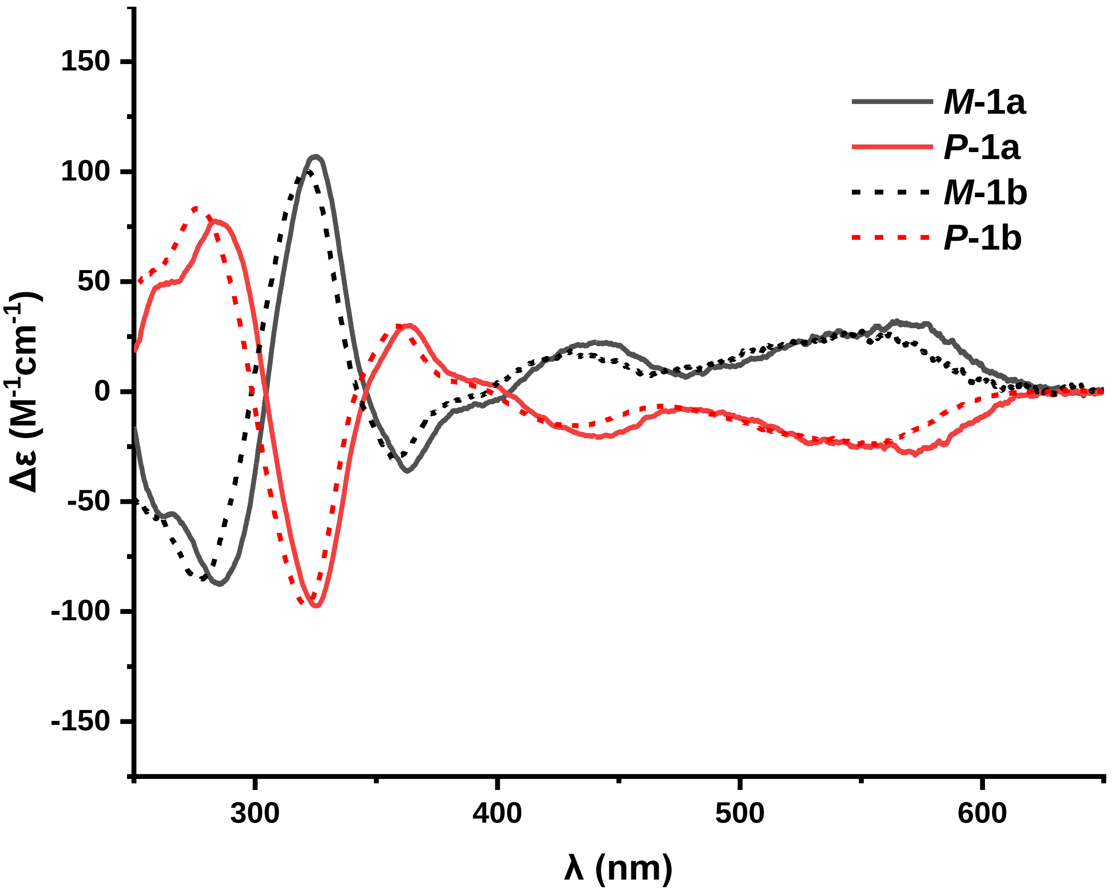

Fig. S18 CD spectra for ***P*** and ***M*** enantiomers of macrocycles **1a** and **1b** in $CHCl_3$ at a concentration of 26 μM, post separation by chiral HPLC (Fig. S24).

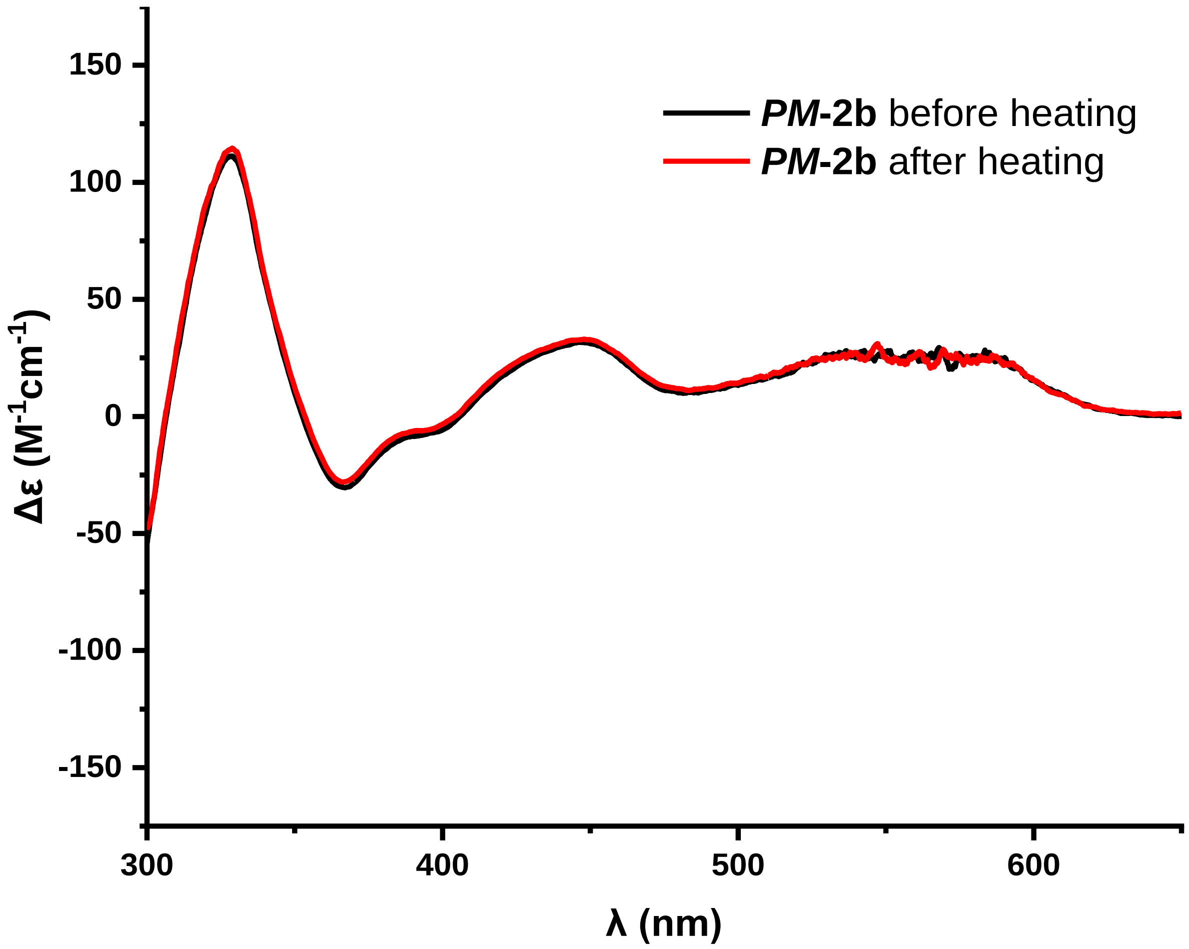

Fig. S19 CD spectra for macrocycle ***PM*-2b** (concentration = 26 μM) in 1,2-dichlorobenzene, prior and after heating to 180 °C for two hours.

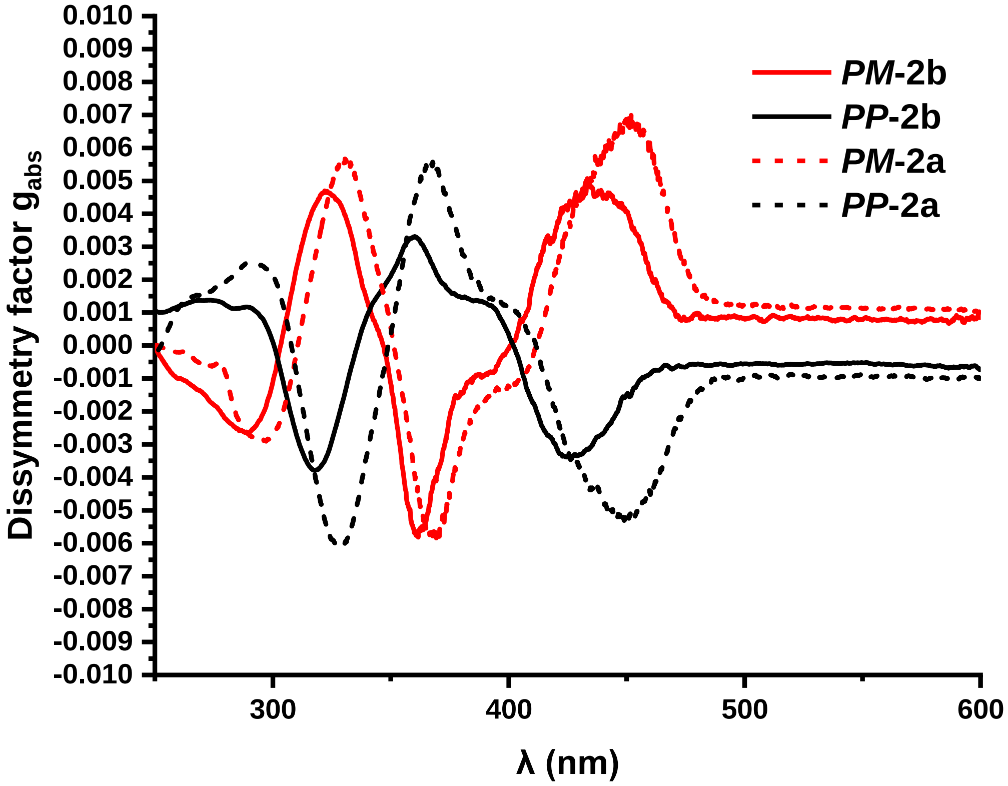

Fig. S20 Full spectra of dissymmetry factor of ***PP*** and ***PM*** diastereomers of macrocycles **2b** and **2a**. The g_{abs} values are: ***PP*-2a** (0.005, 449 nm), ***PM*-2a** (0.007, 453 nm), ***PP*-2b** (0.004, 425 nm), ***PM*-2b** (0.005, 434 nm). The g_{abs} values are comparable to similar compounds in literature.[17-20]

5. UV-vis Spectroscopy

UV-vis spectroscopy was performed on a Cary 5000 spectrophotometer, with a wavelength accuracy ≤ 0.08 nm and absorbance accuracy ≤ 0.01 Abs. A quartz cuvette with 1 cm path length was used.

5.1. Beer-Lambert Plots

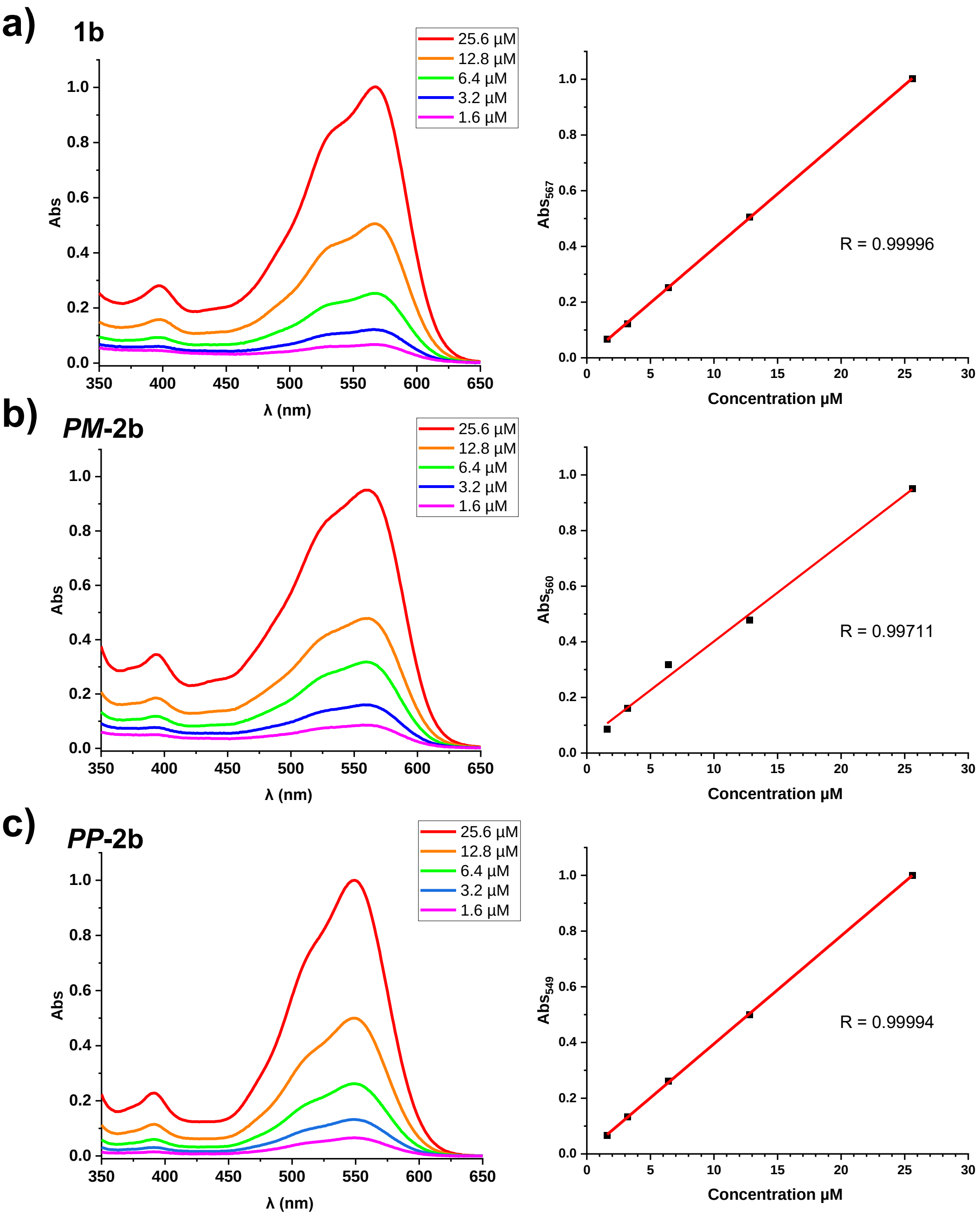

Fig. S21 Left: absorption spectrum of macrocycles: a) **1b**, b) ***PM*-2b** and c) ***PP*-2b** in $CHCl_3$ at different concentrations. Right: the dependence of absorption (λ_{max}) on concentration for these macrocycles: a) **1b** (λ_{max} = 567 nm), b) ***PM*-2b** (λ_{max} = 560 nm), and c) ***PP*-2b** (λ_{max} = 549 nm), in $CHCl_3$ is linear, showing Beer-Lambert behaviour.

5.2. UV-vis Spectra of Macrocycles

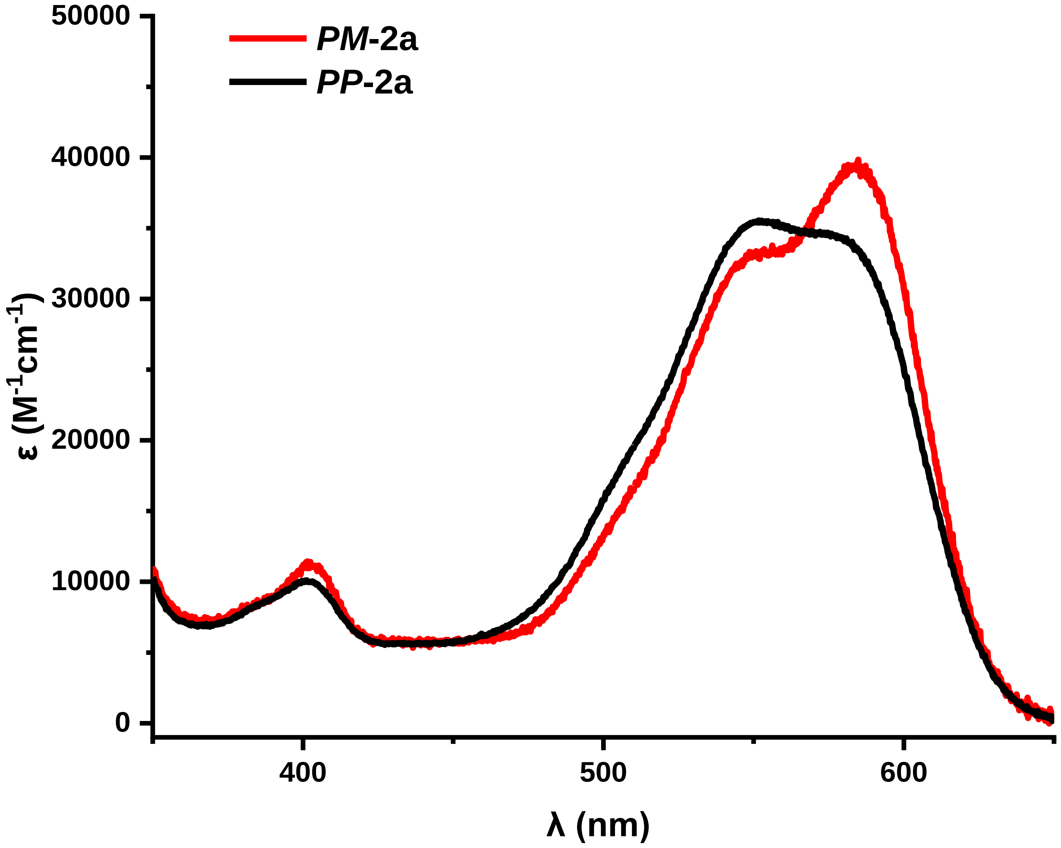

Fig. S22 UV-vis absorption spectra of hetero- and homochiral diastereomers of macrocycle **2a** recorded in $CHCl_3$ at a concentration of 26 μM.

6. Chiral HPLC

Chiral HPLC studies were performed using a Phenomenex i-Amylose-1 chiral column on an Agilent 1290 Infinity analytical HPLC instrument. The flow rate was 1 mL/minute, and the detection wavelength was 500 nm. The eluents for each chromatogram are specified in the figure captions.

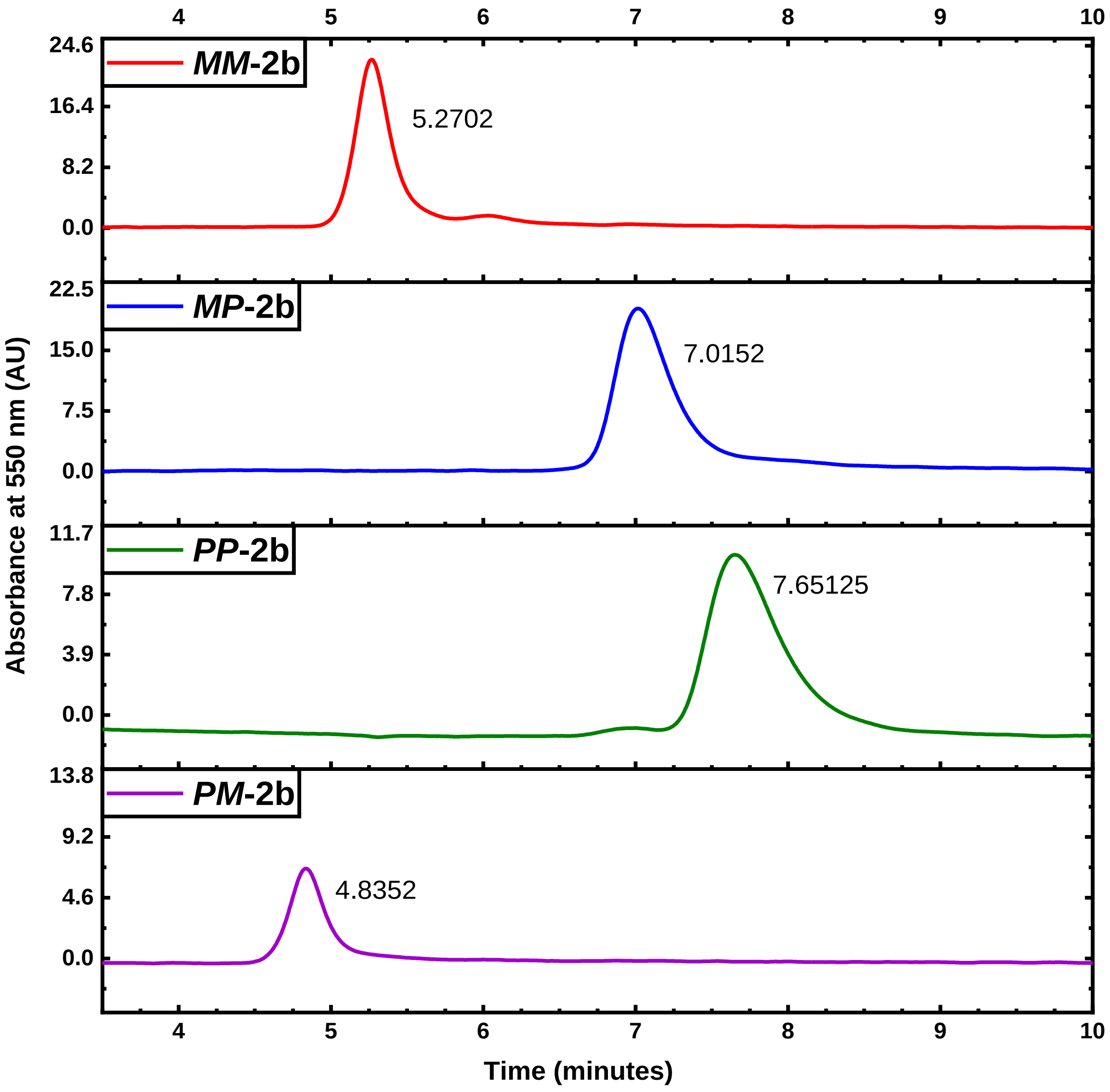

Fig. S23 Chiral HPLC chromatogram of compounds ***MM*-2b** (red), ***MP*-2b** (blue), ***PP*-2b** (green), and ***PM*-2b** (purple), dissolved in DCM and eluted with 0.1:60:39.9 IPA:n-hexane:CH_2Cl_2. Retention times of each compound are shown next to the peaks.

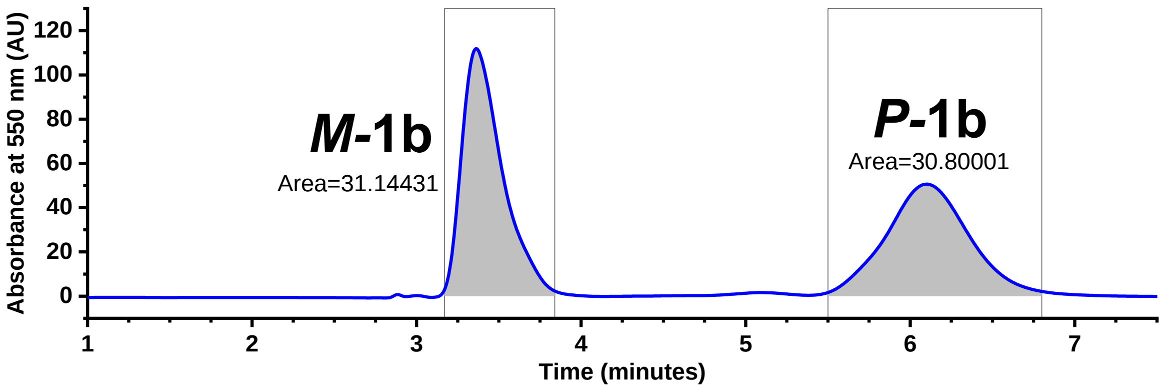

Fig. S24. Chiral HPLC chromatogram of compound **1b** that was isolated as a racemic mixture after column chromatograph (SiO_2), dissolved in DCM and eluted with 2:60:38 IPA:n-hexane:CH_2Cl_2. Using CD spectroscopy (Fig. S18), the first eluted diastereomer at 3.35 min is assigned as the ***M*** enantiomer the other as ***P*** enantiomer. The integration ratio of ***M***:***P*** = 1:1.

7. X-ray Crystallography

Crystals of macrocycle **1a**, suitable for single crystal X-ray diffraction, were grown from a solution of **1a** dissolved in $CHCl_3$, with slow diffusion of n-hexane (antisolvent).

Single Crystal X-Ray Diffraction (SCXRD): Data were collected using a Rigaku Synergy-S dual source with a PhotonJet-S X-ray source and a HyPix-6000 detector. Data were collected at 100 K through the use of an Oxford Cryosystems cryostream device. Data were collected and processed using CrysAlis PRO171.42.49. The structures were solved by direct methods using ShelXT and refined with ShelXL using a least squares method.[21] Olex2 software was used as the solution, refinement and analysis program.[22]

Positional disorder is modelled for one of the straps between the PDI and hydroquinone moieties (carbon atoms C52 – C60 and oxygen atom O5). The occupancies of the two sites were refined and constrained to sum to unity with values of 0.85 and 0.15 respectively. All atoms were refined with anisotropic displacement parameters with the exception of carbon atom C42B and oxygen atom O5B which form part of the low occupancy (part 2) disordered strap which could not be modelled satisfactorily anisotropically. The ADPs of carbons C55B, C56B, C57B, C58B, C59B, C60B of the low occupancy part of the disordered strap were constrained to be equal (EADP) as the shift would not settle if the ADPs of these atoms were only restrained to be similar. Hydrogen atoms were geometrically placed and refined using a riding model.

The occupancy of the chloroform residue (Cl1-3, C67, H67) was allowed to freely refine and settle before being fixed at 0.83. Although evidence for additional molecules of chloroform was seen in the electron density map, these disordered solvent molecules could not be sensibly modelled, so the structure was treated with a Solvent Mask, implemented by Olex. A solvent mask was calculated, and 580 electrons were found in a volume of 1784 Å^3 in 1 void per unit cell. This is consistent with the presence of 2.5 chloroform molecules per Asymmetric Unit which account for 580 electrons per unit cell. Figures were produced using CrystalMakerX.

Crystal Data for macrocycle **1a** $C_{66}H_{62}N_8O_6$**.** 0.83 $CHCl_3$: monoclinic, space group P2/n, a = 14.39140(10) Å, b = 14.94010(10) Å, c = 33.6959(2) Å, α = 90°, β = 91.1850(10)°, γ = 90°, V = 7243.37(8) Å^3, Z = 4, T = 100.00(10) K, μ(Cu Kα) = 1.369 mm^{-1}, *Dcalc* = 1.066 g/cm^3, 134698 reflections measured (5.916° ≤ 2Θ ≤ 156.862°), 14866 unique (R_{int} = 0.0433, R_{sigma} = 0.0229) which were used in all calculations. The final R_1 was 0.0829 (I > 2σ(I)) and wR_2 was 0.2357. Deposited cif number: 2249179.

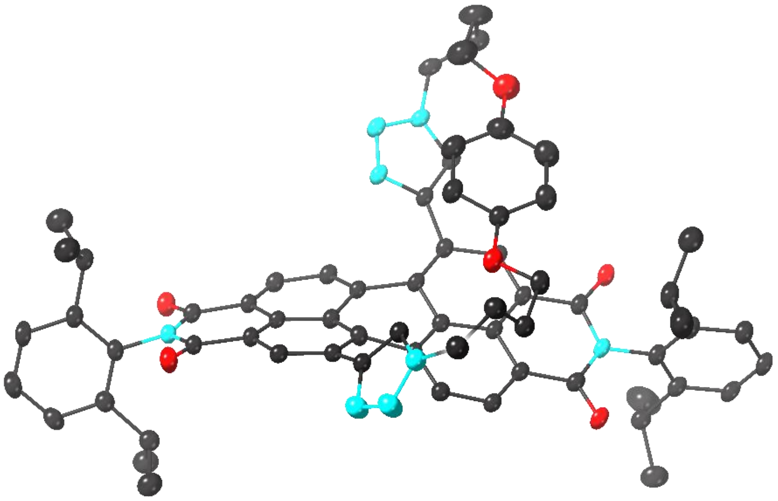

Fig. S25 Asymmetric unit of macrocycle **1a** with all non-hydrogen atoms represented by ellipsoids at the 50% probability level. Hydrogen atoms omitted for clarity (C, dark grey; O, red; N, blue).

8. Density Functional Theory Calculations

Conformer searches for macrocycles **1a**, **2a** and **2b** were performed using the combination of the CREST code[23] and the GFN2-xTB semiempirical tight-binding DFT-method.[24] The lowest energy conformers found using CREST were subsequently reoptimized by means of full DTF using the B97-3c composite scheme.[25] Solvation effects in the DFT calculations were described using the COSMO (toluene, chloroform) implicit solvation models.[26] Vertical excitation and circular dichroism spectra of the DFT optimised conformers were calculated by single point calculations using the combination of the ωB97x[27] density functional and the def2-SVP basis-set.[28] Finally, harmonic frequencies were calculated for both the GFN2-xTB (lowest conformers only) and B973c optimised structures to verify that these optimised structures have no imaginary frequencies and, as expected, correspond to minima on their respective potential energy surfaces. All DFT calculations are performed using Turbomole 7.5.[29-30]

8.1. Conformer Searches

The lowest energy conformer for macrocycle ***M*-1a** as found in the GFN2-xTB CREST conformer search is shown below (Fig. S26). This conformer agrees with the X-ray crystal structure of macrocycle **1a** shown in Fig. 2b (***M*** atropisomer), thereby supporting this method and thus our predictions for macrocycles **2a,b** below (Fig. S27). The conformers of ***M*-1a** and ***P*-1a** are provided as .xyz files in the 'Macrocycle Structures' folder.

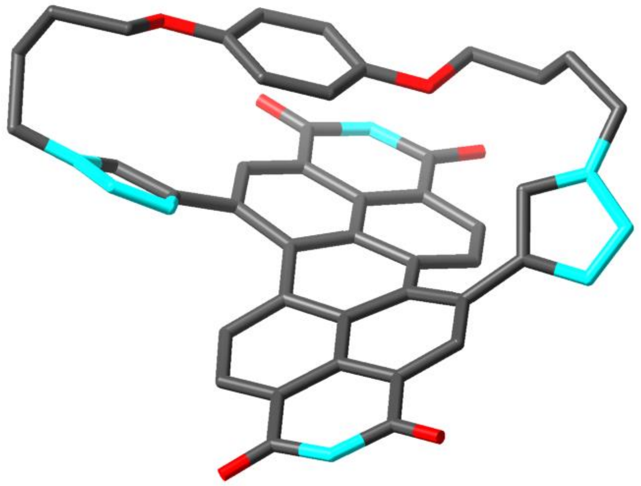

***M*-1a**

Fig. S26 Lowest energy conformer for macrocycle ***M*-1a**, as found in the GFN2-xTB conformer search. For clarity, hydrogen atoms and the 2,6-diisopropylphenyl imide substituents have been removed from the structure.

The lowest energy conformer for macrocycles **2a** and **2b**, found using the same GFN2-xTB CREST conformer search as for ***M*-1a**, are shown below. The conformer search returned in all cases only homochiral conformers (i.e., ***PP*/*MM*-2a** and ***PP*/*MM*-2b**), even when a full conformer search was performed using the heterochiral structure as input, such that heterochiral conformers must lie at least of the order of 20-30 kJ mol^{-1} higher in energy. These conformers are provided as .xyz files in the 'Macrocycle Structures' folder.

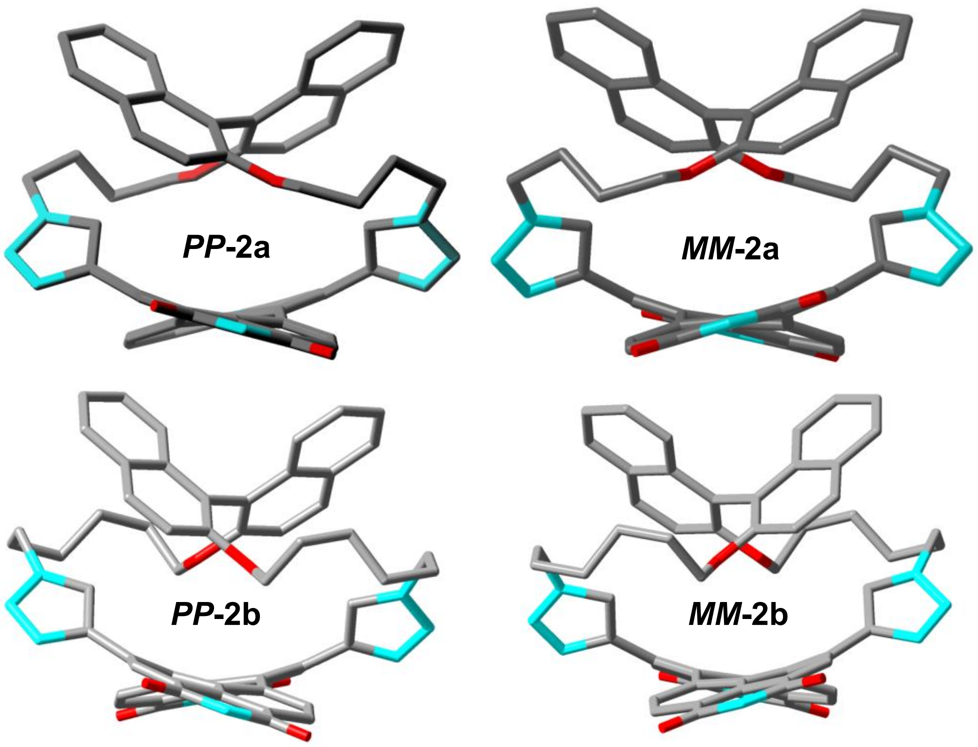

Fig. S27 Lowest energy conformer for macrocycles **2a** and **2b**, as found in the GFN2-xTB conformer search. For clarity, hydrogen atoms and the 2,6-diisopropylphenyl imide substituents have been removed from these structures.

Using the lowest energy conformer ***PP*-2b** as a starting point, a conformer for ***PM*-2b** was manually constructed (***PM*-2b***). Then, a constrained conformer search starting from ***PM*-2b*** was used to obtain our best attempt in achieving the lowest energy conformer of ***PM*-2b** (Fig S28). These conformers, which are not guaranteed to be the lowest energy ***PM*-2b** conformer, are predicted to lie 79 and 65 kJ mol^{-1} respectively above the ***MM*/*PP*-2b** lowest energy structure (B97-3c/$CHCl_3$). Thus, the lowest energy heterochiral conformer must inclusively lie in the energy window between 20 and 65 kJ mol^{-1} above the homochiral conformer. These conformers are provided as .xyz files in the 'Macrocycle Structures' folder.

8.2. Predicted Circular Dichroism Spectra

The predicted circular dichroism spectra of the lowest energy conformer of macrocycles ***MM*-2a** and ***PP*-2a** (Table S1) and ***MM*-2b** and ***PP*-2b** (Table S2). The lowest energy transitions are given since they are exclusive to the PDI chromophore. These predicted CD spectra were used to assign the experimental CD spectra of macrocycles **2a** and **2b** (Supporting Information, Section 4). We note that in all cases the predicted CD spectrum is blue-shifted relative to the experimental spectra, which is a side-effect of the range-separated functional used.

	**Excitation energy / eV (wavelength / nm)**	**Rotary strength / 10^{-40} erg cm^3**	
		***MM*-2b**	***PP*-2b**
1	2.57 (482)	+137	−136
2	3.63 (341)	+81	−81
3	3.71 (334)	+26	-26
4	3.88 (319)	−107	+107
5	3.89 (318)	-99	+99
6	4.14 (300)	-6	+6

Table S1: CD spectra precited for ***MM*-2a** and ***PP*-2a** using ϖB97x/def2-SVP.

	**Excitation energy / eV (wavelength / nm)**	**Rotary strength / 10^{-40} erg cm^3**	
		***MM*-2b**	***PP*-2b**
1	2.53 (490)	+186	−186
2	3.61 (343)	+123	−123
3	3.75 (331)	−71	+71
4	3.87 (321)	−163	+163
5	3.90 (318)	-38	+38
6	3.95 (314)	+8	-8

Table S2: CD spectra precited for ***MM*-2b** and ***PP*-2b** using ϖB97x/def2-SVP.

Tables S3 and S4 give the predicted CD spectra for ***PM*-2b*** and ***PM*-2b**, which show in line with experiment that the sign of the ***PM*** structure (PDI-only region) is opposite to that of the ***PP*** structure, while the magnitude is reduced. The conformers of ***PM*-2b*** and ***PM*-2b** are provided as a .xyz file in the 'Macrocycle Structures' folder.

	**Excitation energy / eV (wavelength / nm)**	**Rotary strength / 10^{-40} erg cm^3**
		PM*-2b
1	2.51 (493)	+162
2	3.62 (342)	+90
3	3.87 (321)	−104
4	3.90 (318)	−95
5	4.08 (304)	-3
6	4.20 (295)	-4

Table S3: CD spectrum predicted for ***PM*-2b*** using ϖB97x/def2-SVP.

	**Excitation energy / eV (wavelength / nm)**	**Rotary strength / 10^{-40} erg cm^3**
		***PM*-2b**
1	2.56 (484)	+76
2	3.55 (349)	+7
3	3.66 (338)	+50
4	3.85 (322)	−11
5	3.95 (314)	−83
6	4.05 (306)	−7

Table S4: CD spectrum predicted for ***PM*-2b** using ϖB97x/def2-SVP.

8.3. Analysis of Structures

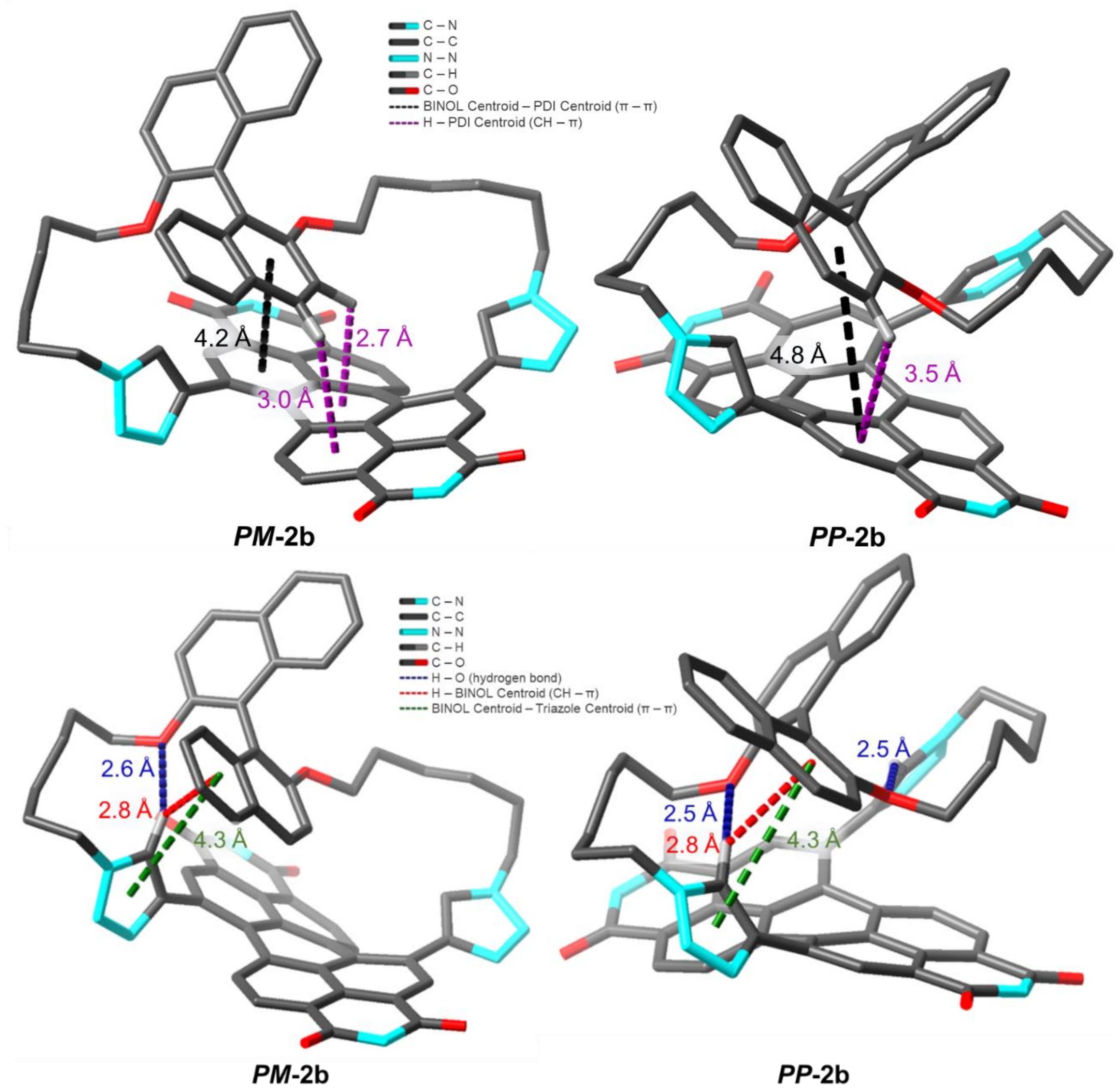

Fig. S28: DFT optimised structures of the lowest energy conformers of ***PM*-2b** and ***PP*-2b**, using in the case of ***PM*-2b** the structure obtained in the constrained conformer search discussed above, showing relevant non-covalent interactions (π–π, CH–π and CH–O hydrogen bonding) between BINOL and PDI (top) and between BINOL and triazole (bottom). Note, the CH–π interactions are represented by close contacts between H and a PDI/BINOL centroid and π–π interactions are represented by close contacts between BINOL/PDI/triazole centroids. For clarity, hydrogen atoms and the 2,6-diisopropylphenyl imide substituents have been removed from these structures.

When considering the energy difference between homo- and heterochiral macrocycle transition states, potential non-covalent interactions involving the triazole moieties (Fig. S28, bottom) were discounted since these heterocycles are formed during the ring-closing of the macrocycle and it is not clear how a triazole intermediate may interact with the BINOL unit. Moreover, if the potential interactions with a *single* triazole heterocycle are considered, since the rate determining step is likely to be the second ring-closing CuAAC ‘click’, then the DFT optimised structures indicate that the triazole–BINOL interactions (Fig. S28, bottom), namely π–π, CH–π, and CH–O hydrogen bonding interactions, are very similar in the homo- and heterochiral macrocyclic structures.

The PDI twist angle (dihedral) increases on decreasing the linker length from hexyl (**2b**) to butyl (**2a**). This angle is also larger in ***PM*-2b** compared to ***PP*-2b**.

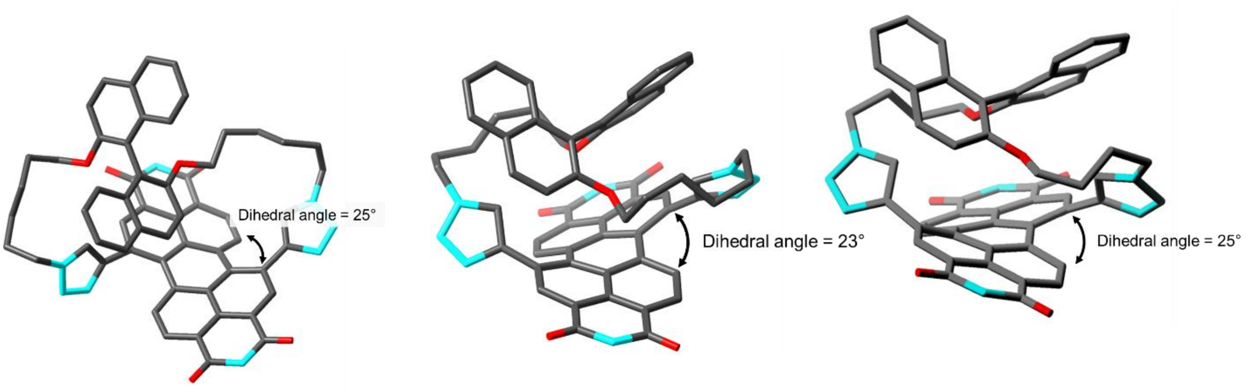

***PM*-2b** ***PP*-2b** ***PP*-2a**

Fig. S29: DFT optimised structures of the lowest energy conformers of ***PM*-2b**, ***PP*-2b**, and ***PP*-2a**, using in the case of ***PM*-2b** the structure obtained in the constrained conformer search discussed above, displaying their PDI core twists of 25°, 23° and 25° respectively. For clarity, hydrogen atoms and the 2,6-diisopropylphenyl imide substituents have been removed from these structures.

8.4. Conformation of BINOL

BINOL may exist in either a transoid (θ < 90°) or cisoid conformer (θ > 90°) as shown in Figure S30a.[31] For our macrocycles, only transoid conformers of BINOL were found in the tight-binding DFT conformer search, i.e., θ = 97° and 102° for ***PP*-2b** and ***PM*-2b** respectively. These dihedral angles are in line with what is typical for a transoid conformer of BINOL[32] (θ ~ 90 - 100°) and indicates that macrocycles with cisoid conformers of BINOL (θ ~ 70°) lie considerably higher in energy. This is also consistent with our ^{1}H NMR spectroscopy analysis since, when transoid BINOL is relatively co-planar with PDI (Fig. S30b), this conformer is able to maximise the BINOL–PDI π–π and CH–π interactions identified from both computational and experimental studies. By contrast, a cisoid BINOL unit interacting with PDI (Fig. S30c) would not only greatly increase ring strain, but would cause desymmetrization, which is not observed by ^{1}H NMR spectroscopy. An alternative cisoid conformer (Fig. S30d) would require the BINOL unit to be positioned away from the PDI, where it would have reduced π–π and CH–π non-covalent interactions, which is not consistent with the significant proton shifts observed by ^{1}H NMR spectroscopy.

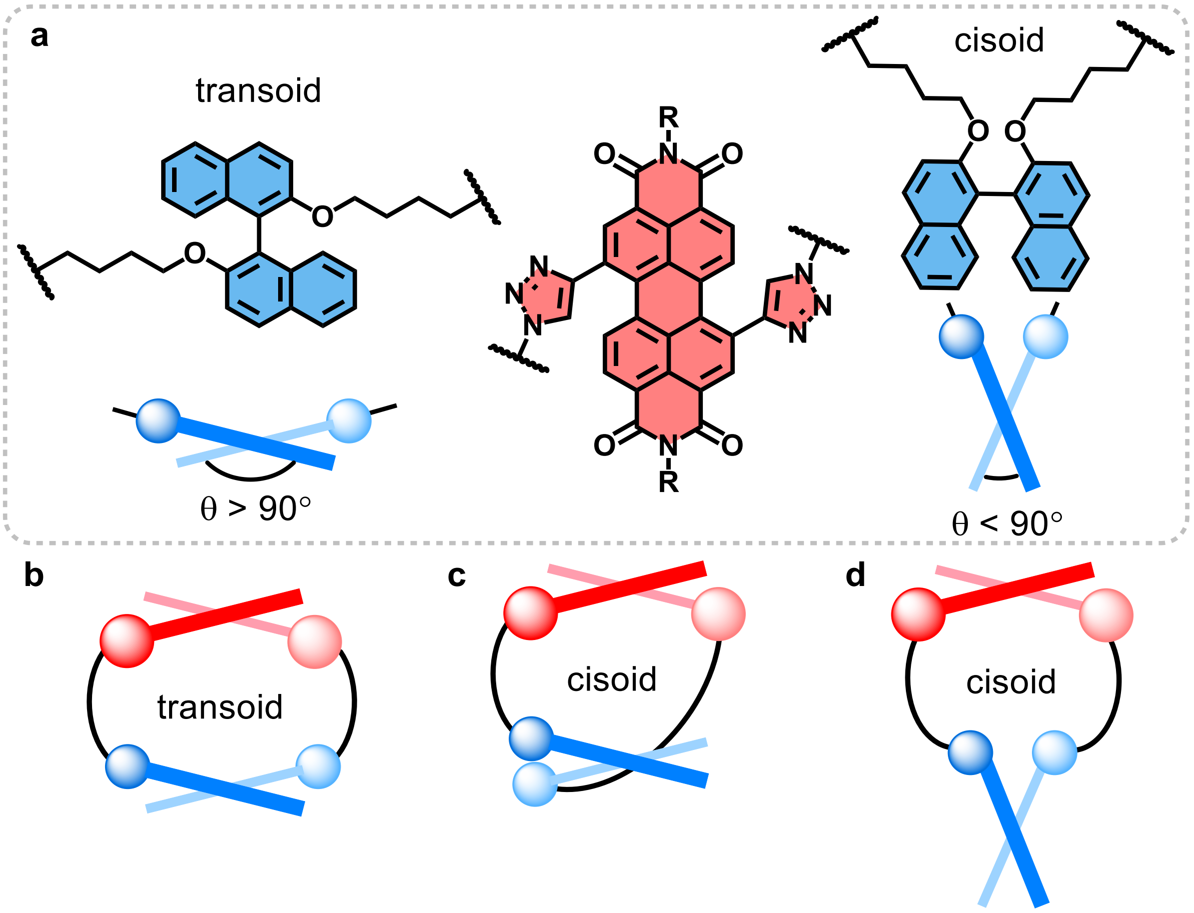

Fig. S30 a) The transoid and cisoid conformers of BINOL; schematics of BINOL–PDI macrocycles where BINOL adopts either, b) a transoid conformer, or c,d) a cisoid conformer.

9. References

1. M. H. Ngai, P. Y. Yang, K. Liu, Y. Shen, M. R. Wenk, S. Q. Yao and M. J. Lear, *Chem. Commun.*, 2010, **46**, 8335-8337.
2. T. R. Chan, R. Hilgraf, K. B. Sharpless and V. V. Fokin, *Org. Lett.*, 2004, **6**, 2853-2855.
3. P. S. Ghosh and A. D. Hamilton, *Chem. Eur. J.*, 2012, **18**, 2361-2365.
4. F. Würthner, V. Stepanenko, Z. Chen, C. R. Saha-Moller, N. Kocher and D. Stalke, *J. Org. Chem.*, 2004, **69**, 7933-7939.
5. A. H. Endres, M. Schaffroth, F. Paulus, H. Reiss, H. Wadepohl, F. Rominger, R. Kramer and U. H. Bunz, *J. Am. Chem. Soc.*, 2016, **138**, 1792-1795.
6. M. Bagui, T. Dutta, H. Zhong, S. Li, S. Chakraborty, A. Keightley and Z. Peng, *Tetrahedron*, 2012, **68**, 2806-2818.
7. H. Kang, W. Jiang and Z. Wang, *Dyes Pigm.*, 2013, **97**, 244-249.
8. Q. Lin, Y. Q. Fan, P. P. Mao, L. Liu, J. Liu, Y. M. Zhang, H. Yao and T. B. Wei, *Chem. Eur. J.*, 2018, **24**, 777-783.
9. C. Gao, S. Silvi, X. Ma, H. Tian, A. Credi and M. Venturi, *Chem. Eur. J.*, 2012, **18**, 16911-16921.
10. Y. Zhou, D. Zhang, L. Zhu, Z. Shuai and D. Zhu, *J. Org. Chem.*, 2006, **71**, 2123-2130.
11. A. Qin, C. K. W. Jim, W. Lu, J. W. Y. Lam, M. Häussler, Y. Dong, H. H. Y. Sung, I. D. Williams, G. K. L. Wong and B. Z. Tang, *Macromolecules*, 2007, **40**, 2308-2317.
12. D. Y. Curtin, *Rec. Chem. Prog.*, 1954, **15**, 111-128.
13. J. I. Seeman, *J. Chem. Educ.*, 1986, **63**, 42.
14. J. I. Seeman, *Chem. Rev.*, 2002, **83**, 83-134.
15. P. Osswald and F. Würthner, *J. Am. Chem. Soc.*, 2007, **129**, 14319-14326.
16. P. Osswald and F. Würthner, *Chem. Eur. J.*, 2007, **13**, 7395-7409.
17. M. Weh, K. Shoyama and F. Würthner, *Nat. Commun.*, 2023, **14**, 243.
18. B. Liu, M. Bockmann, W. Jiang, N. L. Doltsinis and Z. Wang, *J. Am. Chem. Soc.*, 2020, **142**, 7092-7099.
19. S. T. Bao, H. Jiang, C. Schaack, S. Louie, M. L. Steigerwald, C. Nuckolls and Z. Jin, *J. Am. Chem. Soc.*, 2022, **144**, 18772-18777.
20. A. Li, X. Zhang, S. Wang, K. Wei and P. Du, *Org. Lett.*, 2023, **25**, 1183-1187.
21. G. M. Sheldrick, *Acta Crystallogr. A*, 2015, **71**, 3-8.
22. O. V. Dolomanov, L. J. Bourhis, R. J. Gildea, J. A. K. Howard and H. Puschmann, *J. Appl. Crystallogr.*, 2009, **42**, 339-341.
23. P. Pracht, F. Bohle and S. Grimme, *Phys. Chem. Chem. Phys.*, 2020, **22**, 7169-7192.
24. C. Bannwarth, S. Ehlert and S. Grimme, *J. Chem. Theory Comput.*, 2019, **15**, 1652-1671.
25. J. G. Brandenburg, C. Bannwarth, A. Hansen and S. Grimme, *J. Chem. Phys.*, 2018, **148**, 064104.
26. A. Klamt and G. Schüürmann, *J. Chem. Soc., Perkin Trans. 2*, 1993, DOI: 10.1039/p29930000799, 799-805.
27. J. D. Chai and M. Head-Gordon, *J. Chem. Phys.*, 2008, **128**, 084106.
28. F. Weigend and R. Ahlrichs, *Phys. Chem. Chem. Phys.*, 2005, **7**, 3297-3305.
29. F. Furche, R. Ahlrichs, C. Hättig, W. Klopper, M. Sierka and F. Weigend, *Wiley Interdiscip. Rev. Comput. Mol. Sci.*, 2013, **4**, 91-100.
30. S. G. Balasubramani, G. P. Chen, S. Coriani, M. Diedenhofen, M. S. Frank, Y. J. Franzke, F. Furche, R. Grotjahn, M. E. Harding, C. Hattig, A. Hellweg, B. Helmich-Paris, C. Holzer, U. Huniar, M. Kaupp, A. Marefat Khah, S. Karbalaei Khani, T. Muller, F. Mack, B. D. Nguyen, S. M. Parker, E. Perlt, D. Rappoport, K. Reiter, S. Roy, M. Ruckert, G. Schmitz, M. Sierka, E. Tapavicza, D. P. Tew, C. van Wullen, V. K. Voora, F. Weigend, A. Wodynski and J. M. Yu, *J. Chem. Phys.*, 2020, **152**, 184107.
31. H. Hayasaka, T. Miyashita, M. Nakayama, K. Kuwada and K. Akagi, *J. Am. Chem. Soc.*, 2012, **134**, 3758-3765.
32. L. Pu, *Chem. Rev.*, 1998, **98**, 2405-2494.
